# The impact of platelet indices on ischemic stroke: a Mendelian randomization study and mediation analysis

**DOI:** 10.3389/fneur.2023.1302008

**Published:** 2023-12-08

**Authors:** Yang Li, Wenping Xiang, Hui Xue, Tianyu Meng, Tianyou Zhang, Jinfeng Zhang, Jingbo Wang, Jili Zhao, Baojun Wang

**Affiliations:** ^1^Department of Neurology, Inner Mongolia Institute of Cerebrovascular Diseases, Baotou Center Hospital, Baotou, Inner Mongolia, China; ^2^Graduate School, Chongqing Medical University, Chongqing, China; ^3^Graduate School, Baotou Medical College, Baotou, Inner Mongolia, China

**Keywords:** platelet, platelet indices, stroke, blood pressure, Mendelian randomization

## Abstract

**Introduction:**

Platelet indices (PIs) are hematological parameters that indicate the number, morphology, and activation of platelets. Although some clinical trials suggest an association between PIs and the risk of stroke, the lack of robust evidence is attributed to confounding effects and reverse causation.

**Objective:**

This study aimed to evaluate the association between PIs and stroke risk through Mendelian randomization (MR) while exploring the mediating effect of blood pressure in this association.

**Methods:**

We identified genetic variants associated with PIs, including platelet count (PLT), platelet distribution width (PDW), mean platelet volume (MPV), and platelet crit (PCT), in the UK Biobank (*n* = 350,474). Relevant genome-wide association studies were utilized to gather summary statistics pertaining to the traits of interest. We primarily used the inverse-variance weighted analysis to obtain estimates for individual causal power.

**Result:**

We observed a positive correlation between genetically predicted increases in PCT levels with the stroke onset [PCT: OR (95%CI) = 1.113(1.047, 1.183), *p* < 0.001]. However, no significant causal relationship was found between PLT, PDW, and MPV and the risk of stroke [PLT: OR (95%CI) = 1.037(0.979, 1.098), *p* = 0.221; PDW: OR (95%CI) = 0.973(0.923, 1.024), *p* = 0.294; MPV: OR (95%CI) = 0.990(0.945, 1.038), *p* = 0.675]. Multivariable MR analyses and mediation analysis found that the proportion mediated by systolic blood pressure (SBP) is 23.71% [95%CI (10.85–33.31%)] and the proportion mediated by diastolic blood pressure (DBP) is 28.09% [95%CI (12.92–39.63%)].

**Conclusion:**

This large MR study presents evidence for the potential causal relationship between the PCT level and the risk of ischemic stroke, which might be mediated by blood pressure.

## 1 Introduction

According to the 2019 Global Burden of Disease data, stroke remains the second most common cause of mortality globally (1, 2). Acute ischemic stroke (AIS) is among the two main subcategories of stroke (3), which occurs due to focal cerebral hypoperfusion commonly caused by embolism and atherosclerotic disease (4, 5). The onset of arterial thrombosis results from endothelial damage due to vascular injury or pathological atherosclerotic plaque rupture induced by excessive shear stress. The subendothelial tissue is exposed to type I, III, and VI collagen due to this injury, and it binds to platelets through the von Willebrand factor (VWF) (6). The VWF-collagen complex interacts with the platelet glycoprotein receptor Ib-V-IX, facilitating the movement of platelets along the blood vessel wall and allowing the receptors glycoprotein VI (GPVI) and integrin α2β1 to connect with collagen fibers beneath the endothelium (6, 7). After firm adherence and aggregation, platelets produce and release soluble agonists including adenosine diphosphate (ADP), thromboxane A2 (TXA2), and thrombin, which then continuously activate nearby platelets to aggregate and eventually create a clot (8).

Platelets have long been the central focus of therapeutic interventions aimed at preventing thrombotic events. Parameters such as platelet count (PLT), platelet distribution width (PDW), mean platelet volume (MPV), and platelet crit (PCT) are crucial indicators for assessing platelet morphology and proliferation kinetics (9). Heterogeneity in platelet volume within the bloodstream may result in variations in their structures and metabolic functions (9). Previous studies have indicated that MPV may serve as an independent risk factor for the onset of stroke (10, 11). Additionally, some studies have also revealed a potential correlation between cerebral infarction and reduced PLT levels as well as elevated PDW levels (12–14). However, the current research on the impact of PCT on stroke has been scarcely reported.

Hypertension can induce thromboembolism and atherosclerosis, leading to damage to target organs (15). The activation and aggregation of platelets are potential factors contributing to the increased risk of thrombosis in patients with hypertension (16–18). Some studies have indicated a potential correlation between MPV and blood pressure (19, 20). However, despite these investigations conducted, the current clinical evidence regarding the association between PIs and stroke remains inconclusive and lacks longitudinal studies. In addition, the precise role of systolic blood pressure (SBP) and diastolic blood pressure (DBP) in mediating the association between PIs and stroke onset remains uncertain.

The utilization of Mendelian randomization (MR) analysis in evaluating potential causal connections between exposure and outcome has gained widespread popularity. When randomized clinical trials are not available, the utilization of MR analysis, which uses single nucleotide polymorphisms as unconfounded proxies for exposures, becomes an essential approach for establishing causal relationships. The random assortment of genetic variations during meiosis effectively mitigates residual confounding and reverse causality commonly encountered in conventional observational studies.

Therefore, we employed MR analysis utilizing summary statistics from genome-wide association studies (GWAS) to confirm the potential causal relationship between PIs and stroke. Additionally, we assessed the mediating effect of blood pressure in this relationship.

## 2 Methods

### 2.1 Strategy

**Figure 1:**
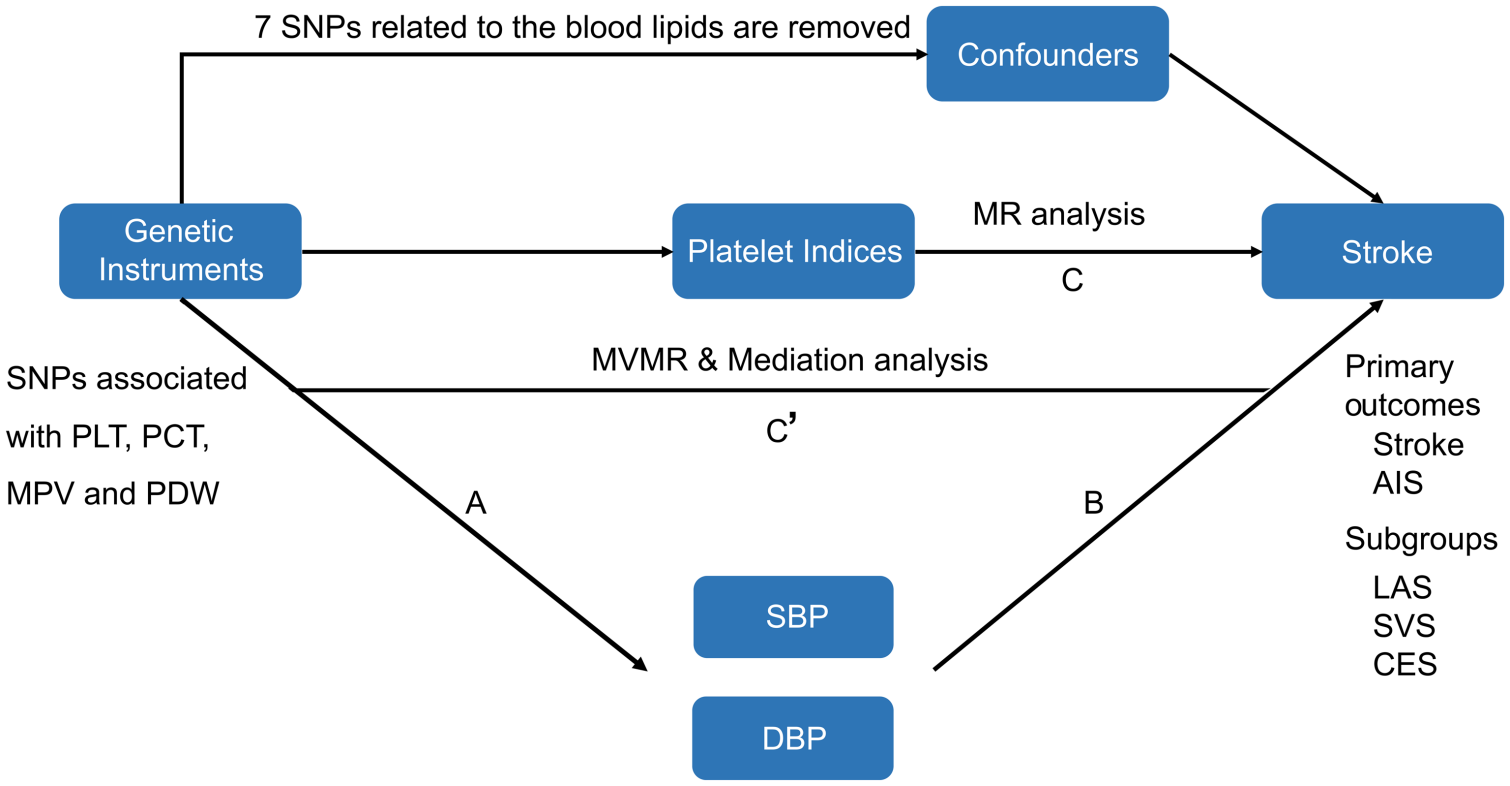
Schematic diagram of the study design. The total effect was decomposed into indirect effect using a two-step approach (“A” is the total effect of PIs on SBP or DBP). “B” is the effect of SBP or DBP on platelet adjusting for stroke. “C” is the total effect of PIs on stroke. “C’ ” the effect of PIs on SBP or DBP adjusting for stroke. Mediation effect = A * B, Proportion mediated was the mediation effect divided by the total effect. MR, Mendelian randomization; MVMR, multivariable Mendelian randomization.

The primary objective was to associate the PIs with stroke by two-sample MR analysis. To achieve this, instrumental variables (IVs) were constructed based on single-nucleotide polymorphisms (SNPs) directly linked to various PIs and strokes. Moreover, we conducted multivariable Mendelian randomization analysis (MVMR) and mediation analysis to examine the possible mediating role of blood pressure in the association between PIs and the risk of stroke. Figure 1 illustrates the study design and assumptions of the MR study.

### 2.2 Data source

The UK Biobank conducted research on PIs in a population of 350,474 individuals with European ancestry using GWAS. Furthermore, a combined total of 757,601 individuals were included in the data provided by the International Consortium of Blood Pressure (ICBP) and UK Biobank, encompassing information on SBP and DBP (21).

The primary outcomes were focused on stroke and ischemic stroke, while the secondary outcomes consisted of three subgroups related to ischemic stroke: large artery atherosclerosis stroke (LAS), small vessel occlusion stroke (SVS), and cardioembolic stroke (CES). The stroke data came from the European population cohort of the MEGASTROKE consortium (22), specifically from individuals of European ancestry. The primary outcomes under assessment were stroke, with a comparison of 40,585 cases and 406,111 controls, and ischemic stroke, with a comparison of 34,217 cases and 406,111 controls.

### 2.3 Selection of instrumental variables

Univariable MR analyses identified independent SNPs achieving genome-wide significance (*p* < 5 × 10^–10^) as IVs for each exposure. SNPs with linkage disequilibrium R2 > 0.0001 were removed using European ancestry reference data acquired from the 10,000 genomes initiative. We thoroughly investigated each IV and its corresponding proxy features in the PhenoScannerV2 database to identify any SNPs associated with potential confounding factors through cross-referencing. To reduce possible bias caused by IVs, we calculated the F-statistic for every SNP and removed those with inadequate instrument strength (*F* < 10). Supplementary Table S1 presents the summarized attributes of the genetic IVs.

### 2.4 Data analysis

This study utilizes a range of MR methods to analyze the causal relationship between PIs and stroke. The inverse variance weighted (IVW) approach was preferred for analysis. Furthermore, the reliability of the findings and potential pleiotropy were assessed using the MR-Egger regression method and weighted median method. A leave-one-out analysis and MR-PRESSO analyses were performed to identify and eliminate any instrumental outliers. A comparable approach was utilized when choosing the IVs for conducting MVMR investigations. The odds ratio (OR) and 95% confidence interval (CI) were presented as estimates of relative risk associated with the disease of interest. To minimize the possible influence of horizontal pleiotropy, an examination was performed to detect horizontal pleiotropy through the MR-Egger analysis or heterogeneity test. To mitigate the potential impact of horizontal pleiotropy, a test for horizontal pleiotropy was conducted using the MR-Egger analysis or heterogeneity test. To evaluate the existence of reverse causation, the Steiger test was employed to determine the existence of a reverse causal connection with a significance level of a *p*-value of <0.05. The result achieved statistical significance with a p-value of less than 0.01 (0.05/5) after conducting the Bonferroni adjustment. A p-value ranging from 0.01 to 0.05 suggests a potential statistical significance, The statistical analyses mentioned were performed using the R (version 4.3.0) packages including two-sample MR and MR.

The two-step multivariable MR and mediation analysis were utilized to estimate the mediated effect of blood pressure on the association between PCT strokes. As depicted in Figure 1, the total effect is the combination of direct and indirect effects. The total effect is the impact of PIs on the stroke without any adjustment. The indirect effect or mediation effect refers to the influence of PIs on stroke that is mediated by blood pressure, and the proportion of mediation was calculated by dividing the mediation effect by the total effect.

## 3 Results

### 3.1 Instrumental variables

In the stroke group, we screened a combined total of 93 SNPs related to PLT, 86 SNPs related to PCT, 94 SNPs related to MVP, and 80 SNPs related to PDW. Seven blood lipid-related SNPs (rs1260326, rs964184, rs10761731, rs780093, rs5130, rs631106, and rs7896518) were identified and excluded through cross-referencing the PhenoScannerV2 database. Figure 1 and Supplementary Table S1 display the quantity of instrumental variables and their phenotypic variances considered in different subgroups. No potential weak instrument bias was indicated as all of these SNPs had F statistics exceeding 10.

### 3.2 Causal relationship between platelet indices and stroke

**Figure 2:**
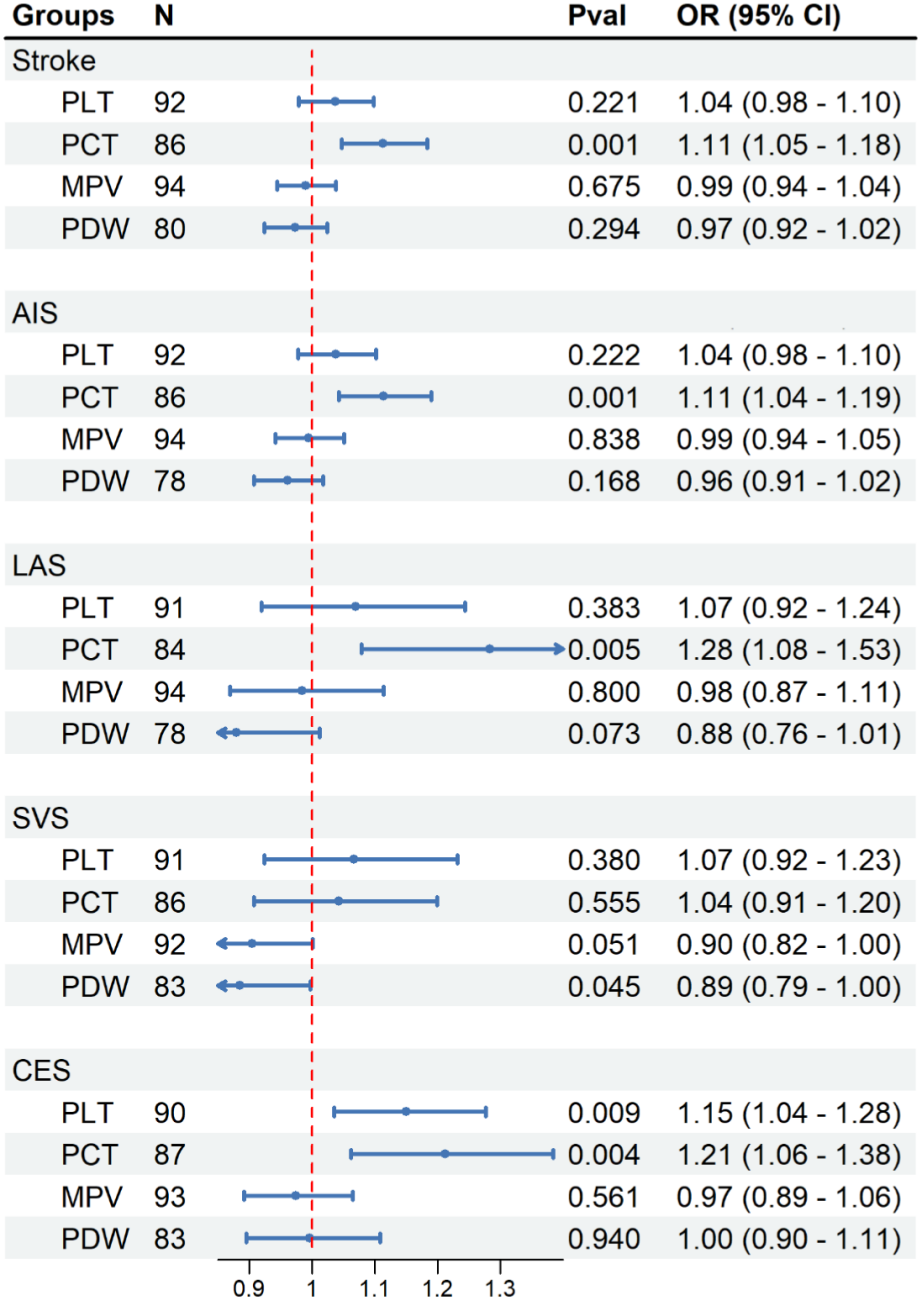
Causal effect of platelet indices on stroke. Methods: Inverse-variance weighted (IVW). N, number of SNPs; N, number of SNP, Pval, *p*-value; OR, odds ratio; CI, confidence interval.

The correlation between PIs and various types of strokes is depicted in Figure 2. By employing the IVW technique, we noticed a significant positive correlation between the PCT level with the occurrence of stroke [PCT OR (95%CI) = 1.113(1.047, 1.183), *p* < 0.001]. There were no associations between PLT, MPV, and PDW and the stroke onset [PLT OR (95%CI) = 1.037(0.979, 1.098), *p* = 0.221; MPV OR (95%CI) = 0.990(0.945, 1.038), *p* = 0.675; PDW OR (95%CI) = 0.973(0.923, 1.024)].

We have observed a direct correlation between PCT levels and the occurrence of developing AIS, LAS, and CES in different stroke subtypes [AIS: OR (95%CI) = 1.114 (1.043, 1.190), *p* < 0.001; LAS: OR (95%CI) = 1.128 (1.079, 1.527), *p* < 0.01; CES: OR (95%CI) = 1.212 (1.062, 1.384), *p* < 0.01; Figure 2]. Similar findings were observed when examined with alternative supplementary methods. However, there was no significant correlation found between PCT and the SVS [OR (95% CI) = 1.043 (0.907–1.199), *p* = 0.555; Figure 2].

### 3.3 Causal relationship between PCT and blood pressure

**Figure 3:**
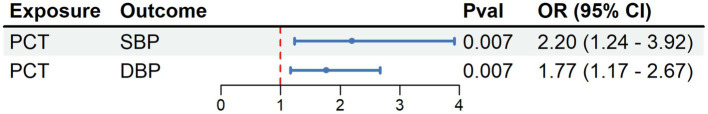
Causal effect of platelet crit on SBP and DBP. N, number of SNPs.

The MR analysis revealed a significant association between PCT levels and both SBP and DBP. For each additional SD increase in the PCT level, the odds ratios for SBP and DBP were 2.202 (95% CI = 1.238, 3.917; *p* < 0.01) and 1.769 (95% CI = 1.170–2.674; *p* < 0.01; Figure 3).

### 3.4 MVMR and mediation analysis result

As previously mentioned, we have observed significant positive associations between the PCT index and both SBP and DBP levels (Figure 3). As presented in Table 1, it is not unexpected to observe the causal association between elevated blood pressure and all types of stroke onset. To further explore the underlying mechanism between the PCT level and the development of stroke, we constructed three predictive models: the SBP-PCT model, the DBP-PCT model, and the SBP-DBP-PCT model. To assess these models, we employed mediation analysis methods and two-step multivariable Mendelian randomization.

**Table 1:** MR analyses for the associations between SBP and DBP and stroke.

Exposure	Outcome	*N*	B	Pval	OR (95%CI)
SBP	Stroke	95	0.035	<0.001	1.036 (1.029, 1.042)
	LAS	95	0.062	<0.001	1.064 (1.048, 1.081)
	AIS	95	0.038	<0.001	1.039 (1.032, 1.046)
	SVS	95	0.041	<0.001	1.042 (1.027, 1.057)
	CES	95	0.027	<0.001	1.028 (1.015, 1.041)
DBP	Stroke	93	0.05	<0.001	1.051 (1.038, 1.065)
	LAS	93	0.064	<0.001	1.066 (1.038, 1.095)
	AIS	93	0.055	<0.001	1.057 (1.043, 1.071)
	SVS	93	0.071	<0.001	1.074 (1.046, 1.102)
	CES	93	0.044	<0.001	1.045 (1.022, 1.069)

MR analysis for the associations between SBP and DBP and stroke; the method is inverse-variance weighted. MR, Mendelian randomization; N, number of SNP; Pval, P-value; OR, odds ratio; CI, confidence interval.

**Figure 4:**
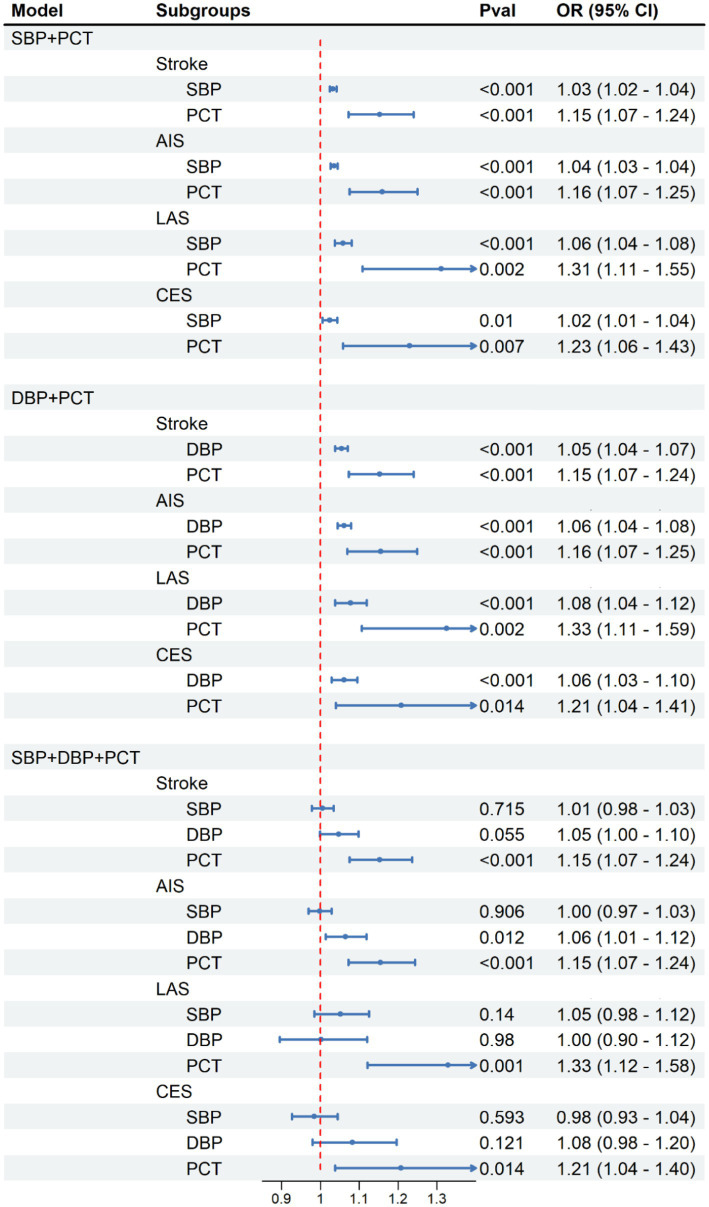
Causal effect of platelet crit on SBP and DBP in MVMR analysis. Methods: Inverse-variance weighted (IVW).

The positive associations between elevated PCT levels and the risk of stroke, AIS, LAS, and CES remained statistically significant even after accounting for the mediating effects of SBP or DBP (Figure 4). The results remained consistent when considering both blood SBP and DBP simultaneously.

When excluding the impact of PCT, there exists a positive correlation between elevated levels of SBP or DBP and an increased risk of stroke onset. Due to the significant association between SBP and DBP, it becomes difficult to isolate the impact of DBP or SBP on stroke individually. Consequently, after accounting for the impacts of PCT and SBP in the SBP-DBP-PCT model, there was no notable association observed between DBP and the occurrence of stroke (Figure 4). Similarly, there was no significant association between SBP and stroke when accounting for the impacts of PCT and DBP.

### 3.5 The proportion mediated by SBP and DBP

**Figure 5:**
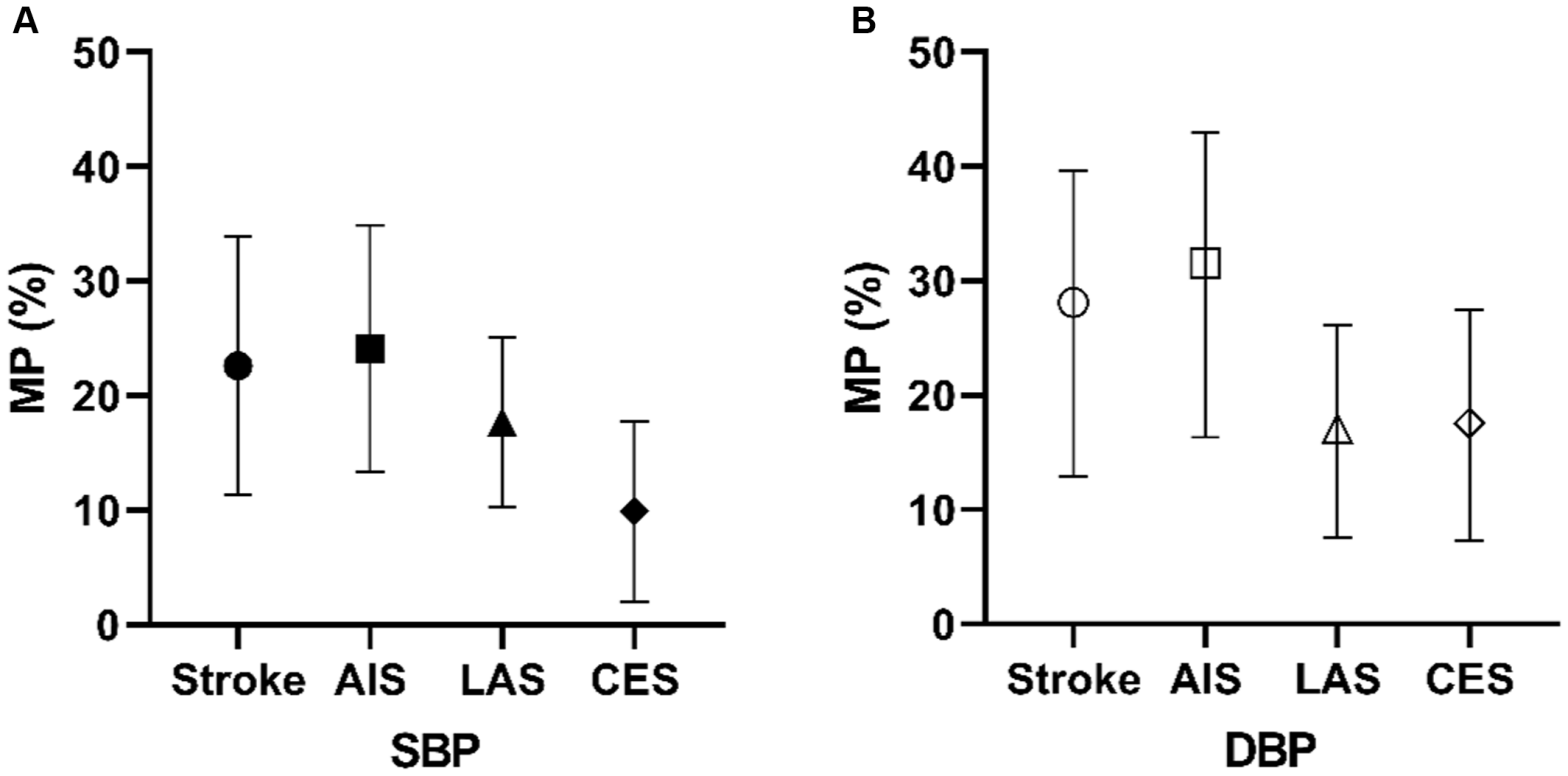
Mediating proportion of SBP **(A)** and DBP **(B)** in PCT and different types of stroke models. MP, mediation proportion.

We examined the proportion mediated by different combinations of mediating variables. As shown in Figure 5A, in the MVMR analysis of the PCT-SBP model, the direct effect of PCT on stroke was 0.082 after accounting for SBP. The proportion mediated by SBP is 23.71% [95%CI (10.85–33.31%)]. In the subgroups of AIS, LAS, and CES, the proportions mediated by SBP are 25.2% [95%CI (12.82–34.32%)], 17.98% [95%CI (10.19–24.98%)], and 9.84% [95%CI (2.01–17.79%)], respectively.

In the MVMR analysis of the PCT-DBP model (Figure 5B), the proportion mediated by DBP is 28.09% [95%CI (12.92–39.63%)]. The direct effect of PCT on stroke was 0.077 after accounting for DBP. In the subgroups of AIS, LAS, and CES, the proportions mediated by DBP are 31.57% [95%CI (16.29–42.96%)], 17.06% [95%CI (7.61–26.16%)], and 17.56% [95%CI (7.29–27.46%)], respectively.

### 3.6 Sensitivity analyses

In the stroke groups, significant heterogeneity in the SNPs effect estimates of PLT (Q = 120.86, *p* = 0.02), MPV (Q = 131.1, *p* < 0.001), PDW (Q = 102.77, *p* = 0.04) were observed according to the Cochran’s Q test (Supplementary Table S2). More importantly, we found no heterogeneity of PCT in the stroke group. Furthermore, the evaluation of horizontal pleiotropy using the MR-Egger regression intercept did not identify any substantial evidence of pleiotropy (Supplementary Table S3).

## 4 Discussion

Platelets have a prominent role in initiating thrombus formation, which could lead to the occurrence of ischemic stroke (23). This study demonstrated a significant positive causal association between PCT and the onset of stroke. However, in contrast to previous studies, we did not observe the causal relationship between levels of PLT, MPV, PDW, and the onset of stroke. Furthermore, the present study suggests that the level of PCT is causally linked to SBP and DBP. We also found that blood pressure may be a mediating factor between PCT and stroke onset.

Previous studies have shown reduced PLT levels in patients with ischemic stroke and coronary artery disease (14). In addition, MPV/PLT has been utilized as a platelet ratio to predict the stroke prognosis (24). However, our research did not establish a causal relationship between PLT and stroke. In addition, MPV serves as an indicator of the average platelet size, with elevated levels indicating increased platelet activity and aggregation in patients (11, 16). Although no significant correlation between PLT and MPV and stroke was found in this study, the potential causal relationship between PCT and stroke may partly explain the inconsistencies between the results of MPV and PLT and the conclusion of previous studies.

PCT represents the platelet volume fraction in the blood. Its measurement is calculated by multiplying the PLT with MPV and expressing it as a percentage (25). When analyzed solely based on the PCT formula, a positive correlation between PCT and MPV is observed, suggesting that PCT may serve as a crude indicator of MPV. Similar to Eric’s findings (26), we found a significant association between PCT and stroke onset. Therefore, MPV or PLT alone may not possess sufficient strength to establish a causal relationship with stroke.

Previous studies have shown that an increase in the PDW level may enhance the adhesive strength of platelet aggregation during white thrombus formation, while it has also been found that oxidative stress can lead to a decrease in platelet deformability and subsequently elevate PDW levels (12). However, this study did not find any significant association between PDW and stroke risk through MR analysis. The observed discrepancy may be ascribed to previous investigations exploring the association between low-density lipoprotein cholesterol (LDL-c) and PDW, which suggests that LDL-c potentially influences PDW levels in stroke patients (12). To ensure the validity of core assumption 2 of MR analysis, this study excluded IVs related to the confounding factor of blood lipid, which might have potentially influenced the observed discrepancies in outcomes.

In addition to the activation and aggregation of platelets, the involvement of PCT level in the pathogenesis of atherosclerosis is also worth considering. For one thing, the development of atherosclerosis is significantly influenced by persistent inflammation (27). Monocytes, also known as myelocytes, are precursors to macrophages, meaning a common ancestral cell lineage shared by leukocytes and platelets (28). If inflammatory pathways are not strictly confined, platelets could be regarded as an extension of the cellular immune system (29). Platelet activation could release a variety of inflammatory mediators locally, promoting leukocyte chemotaxis, adhesion, and translocation to the site of inflammation. Consequently, platelets could be involved in the inflammatory response that could impact the progression of atherosclerosis and the remodeling of blood vessels (30). Barrett (31) found that the suppressor of cytokine signaling 3 (SOCS3) is a protein that triggers inflammatory reactions in atherosclerotic plaques. Platelet-induced expression of SOCS 3 could enhance the production of inflammatory cytokines (TNF-α, IL1b, and IL-6), leading to a phenotypic switch toward inflammation in plaque macrophages and ultimately resulting in sustained inflammation and plaque growth (30). Numerous research studies have indicated that measuring PCT levels could serve as a biomarker for assessing the inflammatory response in some diseases, such as Crohn’s disease, tuberculosis, and sepsis (32). Additionally, the PCT level exhibits a positive correlation with some inflammatory markers such as C-reactive protein (CRP) (33). Therefore, it is speculated that PCT may serve as an indicator for the progression of the inflammatory response associated with atherosclerosis, thereby providing potential explanations for the association between PCT and LAS. Moreover, the causal relationship between the PCT and SVS is not found in this research. Arterioles are integral components of the microvascular bed, primarily consisting of smooth muscle cells that possess the ability to regulate tissue perfusion (34). Pathological alterations commonly observed in the small vessel system encompass arteriolosclerosis and fibrinoid necrosis, leading to luminal narrowing and thickening (35). Arteriosclerosis is predominantly attributed to hypertension, while atherosclerosis is recognized as a chronic inflammatory ailment involving both innate and adaptive immunity (36, 37). This distinction may elucidate the absence of a causal association between PCT and the onset of SVS, while a causal relationship between PCT and LAS is established.

Non-rheumatic atrial fibrillation is a prominent etiological factor for cerebrovascular embolic stroke (CES), with anticoagulation being the preferred treatment modality over antiplatelet therapy (38). However, our research revealed a potential causal correlation between PLT and PCT levels and the onset of CES. The underlying reason for this seemingly paradoxical finding remains elusive. We speculate that this might be due to the mechanisms of influence of genetic variation and clinical therapeutic interventions on exposure tend to differ. Consequently, genetic exposure and clinical intervention may exert disparate physiological or pathological influences on outcomes. Several studies found that systemic inflammatory processes also promote incident and recurrent atrial fibrillation (AF) (39). Thus, the causal relationship between the PCT and CES may be primarily through the inflammatory response pathway of AF, rather than platelet aggregation or activation. However, the interpretation of this result warrants caution as further investigation is needed in future.

Hypertension remains a primary cause of both atherosclerosis and strokes. A 10 mm Hg reduction in SBP is associated with a 33% decrease in the risk of experiencing a stroke (40). The present study suggests that the level of PCT is causally linked to the SBP and DBP, which are in line with Xu’s findings (16). We also found that blood pressure may be a mediating factor between PCT and stroke onset. Subgroup analysis found that the blood pressure mediating effect accounted for the highest proportion of AIS. A study by Alpsoy et al. (18) indicated that patients with hypertension frequently experience increased sympathetic activation during nighttime, which can lead to platelet activation and subsequently affect platelet indices. Hence, this could also elucidate the function of PCT in stroke. However, further research is required to ascertain the possible influence of antiplatelet treatment on blood pressure. This could potentially create a new aspect for future interventions in antiplatelet therapy.

The present study is the first to attempt to investigate the causal relationship between platelet indication and stroke through Mendelian randomization analysis. However, it is important to recognize a few limitations of this study. The generalizability of our findings to other populations may be limited due to the inclusion of only patients of European descent. Furthermore, the potential for sample duplication leading to bias and the presence of heterogeneity are critical factors that restrict the reliability of research findings.

Importantly, hematology analyses employ impedance counting or optical light scatter counting methods for quantifying PIs. Therefore, platelet data from different analytical methods may interfere with the results (9). In addition, EDTA is used as an anticoagulant during blood sample collection, which may lead to platelet swelling and elevated MPV values, but this confounding factor cannot be controlled in this MR study (16). Therefore, although platelet indices were not directly measured in this experiment, standard specimen collection procedures are required in future clinical trials to ensure consistency, such as the use of standardized specimen tubes and analysis of samples 30 min after blood collection. In addition, calibration procedures may be required to guarantee the accuracy of the analytical results.

Finally, although antiplatelet treatment is essential in preventing and treating stroke, it would be worthwhile to investigate the potential of PIs as an indicator for assessing the effectiveness of antiplatelet therapy. In addition, it is important to note that this analysis solely considers one possible intermediate factor, but risk factors such as diabetes, lipid metabolism, body mass index (BMI), and other risk factors for cerebrovascular disease were not included in the study. Previous studies have also found that in patients with diabetes, platelet indices such as PCT and MPV could be used as markers to evaluate diabetic vascular complications (41, 42). Consequently, PCT might serve as a promising aspect for future research in this field.

## 5 Conclusion

In summary, this Mendelian randomization analysis provides evidence for establishing a potential causal relationship between PCT levels and increased stroke risk, which may be mediated by blood pressure. Moreover, there might exist a possible association between PCT levels and blood pressure. Therefore, PCT might serve as an indicator for stroke prevention and evaluation of the effect of antiplatelet treatment; however, further clinical validation is required.

## Data availability statement

The original contributions presented in the study are included in the article/Supplementary material, further inquiries can be directed to the corresponding author.

## Ethics statement

The studies involving humans were approved by the Ethics Committee of Baotou Central Hospital (approval number: KYLL2023073). The studies were conducted in accordance with the local legislation and institutional requirements. Written informed consent for participation was not required from the participants or the participants' legal guardians/next of kin in accordance with the national legislation and institutional requirements.

## Author contributions

YL: Formal analysis, Investigation, Software, Writing – original draft. WX: Conceptualization, Methodology, Writing – original draft. HX: Formal analysis, Investigation, Writing – original draft. TM: Data curation, Software, Writing – original draft. TZ: Data curation, Software, Writing – original draft. JinZ: Project administration, Supervision, Validation, Writing – review & editing. JW: Funding acquisition, Supervision, Writing – review & editing. JilZ: Project administration, Validation, Writing – review & editing. BW: Conceptualization, Funding acquisition, Methodology, Writing – review & editing.
