# Supplementary material for: The impact of platelet indices on ischemic stroke: a Mendelian randomization study and mediation analysis
Source: Front Neurol. 2023 Dec 8;14:1302008. doi: 10.3389/fneur.2023.1302008 (PMC10741650; doi:10.3389/fneur.2023.1302008)
Supplement: Supplementary file 1 [file Table_1.pdf]

Table S1. SNPs information of platelet indices (PLT, PDW, MPV and PCT) with Stroke and stroke subtypes group (ALS, LAS, SVS and CES)

Exposure	Outcome	SNP	Effect allele	Other allele	Beta	SE	Eaf	N	*P* val	Fval	R2
PLT	Stroke	rs10840160	C	T	-0.023	0.002	0.462	350474	6.361E-23	91.500	0.0003
PLT	Stroke	rs11071720	C	T	0.038	0.003	0.702	350474	1.46E-51	213.858	0.0006
PLT	Stroke	rs11082304	T	G	-0.052	0.002	0.514	350474	9.77E-113	475.721	0.0014
PLT	Stroke	rs114694170	C	T	0.144	0.005	0.059	350474	1.11E-186	811.444	0.0023
PLT	Stroke	rs115365432	G	T	-0.029	0.004	0.102	350474	6.425E-14	52.881	0.0002
PLT	Stroke	rs11553699	G	A	-0.110	0.004	0.135	350474	1E-200	991.239	0.0028
PLT	Stroke	rs11604127	T	C	0.092	0.003	0.232	350474	1E-200	1055.154	0.003
PLT	Stroke	rs11731274	G	T	0.052	0.003	0.176	350474	1.504E-65	274.024	0.0008
PLT	Stroke	rs11841319	T	C	-0.061	0.004	0.103	350474	1.136E-57	241.375	0.0007
PLT	Stroke	rs12065863	C	T	-0.037	0.003	0.251	350474	1.947E-43	179.545	0.0005
PLT	Stroke	rs12569998	G	T	0.020	0.003	0.158	350474	1.608E-10	38.279	0.0001
PLT	Stroke	rs12666044	A	T	0.028	0.002	0.665	350474	2.234E-29	123.126	0.0004
PLT	Stroke	rs12748176	C	T	0.056	0.005	0.056	350474	1.107E-28	115.285	0.0003
PLT	Stroke	rs148762611	G	A	-0.019	0.002	0.347	350474	7.63E-15	56.840	0.0002
PLT	Stroke	rs151234	C	G	0.065	0.003	0.129	350474	3.843E-78	328.655	0.0009
PLT	Stroke	rs1558328	T	C	-0.052	0.002	0.497	350474	1.94E-111	473.834	0.0014
PLT	Stroke	rs16978075	C	T	0.025	0.004	0.110	350474	1.269E-11	43.154	0.0001
PLT	Stroke	rs17652304	G	C	-0.087	0.005	0.058	350474	1.49E-67	286.960	0.0008
PLT	Stroke	rs1768584	G	A	0.051	0.002	0.386	350474	1.36E-102	432.078	0.0012
PLT	Stroke	rs2027939	A	G	-0.031	0.003	0.142	350474	3.767E-20	79.915	0.0002
PLT	Stroke	rs2075672	G	A	0.027	0.002	0.624	350474	2.083E-30	122.654	0.0003
PLT	Stroke	rs2155380	G	A	0.054	0.003	0.271	350474	6.234E-94	397.056	0.0011
PLT	Stroke	rs2236055	G	A	-0.038	0.002	0.468	350474	5.254E-61	253.695	0.0007
PLT	Stroke	rs2297066	G	C	0.052	0.003	0.242	350474	2.568E-82	345.542	0.001
PLT	Stroke	rs2535403	C	T	-0.021	0.002	0.555	350474	5.131E-19	74.731	0.0002
PLT	Stroke	rs2631360	A	G	0.027	0.002	0.519	350474	1.398E-32	132.239	0.0004
PLT	Stroke	rs2699425	C	T	0.021	0.002	0.373	350474	4.435E-19	75.673	0.0002
PLT	Stroke	rs2724564	T	G	0.022	0.002	0.561	350474	7.695E-21	82.102	0.0002
PLT	Stroke	rs2841102	C	T	0.019	0.002	0.439	350474	1.336E-16	64.074	0.0002
PLT	Stroke	rs301369	G	A	0.041	0.002	0.410	350474	2.275E-67	280.804	0.0008
PLT	Stroke	rs3205136	A	C	-0.036	0.004	0.094	350474	3.739E-19	76.858	0.0002
PLT	Stroke	rs342293	G	C	-0.076	0.002	0.459	350474	1E-200	999.347	0.0028
PLT	Stroke	rs346738	C	T	0.033	0.002	0.655	350474	5.378E-42	175.581	0.0005
PLT	Stroke	rs346744	A	G	-0.022	0.002	0.582	350474	9.406E-21	82.809	0.0002
PLT	Stroke	rs4148445	T	C	0.064	0.004	0.918	350474	3.74E-53	220.278	0.0006
PLT	Stroke	rs4257458	G	A	0.023	0.002	0.390	350474	6.693E-22	87.597	0.0002
PLT	Stroke	rs4272720	G	A	-0.031	0.003	0.237	350474	1.879E-30	124.514	0.0004
PLT	Stroke	rs4309234	T	C	-0.035	0.003	0.781	350474	2.54E-35	145.072	0.0004
PLT	Stroke	rs4592028	C	T	0.017	0.002	0.330	350474	6.121E-12	44.585	0.0001
PLT	Stroke	rs479404	C	T	-0.018	0.003	0.270	350474	1.494E-11	42.930	0.0001
PLT	Stroke	rs4818031	G	A	-0.018	0.002	0.321	350474	6.742E-13	48.980	0.0001
PLT	Stroke	rs4820268	A	G	-0.025	0.002	0.537	350474	7.114E-27	107.544	0.0003
PLT	Stroke	rs4871590	G	A	0.016	0.002	0.578	350474	2.112E-11	42.552	0.0001
PLT	Stroke	rs4905994	C	T	0.036	0.002	0.683	350474	7.003E-48	199.107	0.0006
PLT	Stroke	rs4906254	G	A	-0.031	0.002	0.347	350474	9.986E-37	150.438	0.0004
PLT	Stroke	rs4935968	T	C	-0.026	0.002	0.517	350474	6.992E-30	120.641	0.0003

Table S1. SNPs information of platelet indices (PLT, PDW, MPV and PCT) with Stroke and stroke subtypes group (ALS, LAS, SVS and CES)

Exposure	Outcome	SNP	Effect allele	Other allele	Beta	SE	Eaf	N	*P* val	Fval	R2
PLT	Stroke	rs511515	G	A	0.098	0.003	0.699	350474	1E-200	1422.493	0.004
PLT	Stroke	rs55660207	G	C	0.058	0.005	0.071	350474	4.307E-38	156.098	0.0004
PLT	Stroke	rs55941898	T	C	0.025	0.003	0.140	350474	5.4E-14	53.365	0.0002
PLT	Stroke	rs56043070	A	G	-0.127	0.004	0.072	350474	3.24E-178	756.500	0.0022
PLT	Stroke	rs57843631	T	C	-0.225	0.009	0.019	350474	1.44E-147	672.478	0.0019
PLT	Stroke	rs6070696	G	A	0.051	0.003	0.186	350474	4.576E-66	277.495	0.0008
PLT	Stroke	rs60757417	G	C	-0.081	0.005	0.060	350474	2.384E-61	260.984	0.0007
PLT	Stroke	rs6119728	A	G	-0.030	0.003	0.291	350474	2.681E-31	126.551	0.0004
PLT	Stroke	rs6141	T	C	0.056	0.002	0.530	350474	2.78E-126	542.840	0.0015
PLT	Stroke	rs61869602	C	T	0.016	0.002	0.407	350474	3.015E-11	41.608	0.0001
PLT	Stroke	rs61963261	T	C	0.022	0.003	0.159	350474	4.079E-12	44.979	0.0001
PLT	Stroke	rs62048972	T	C	0.039	0.003	0.164	350474	1.221E-36	150.076	0.0004
PLT	Stroke	rs62175229	T	C	-0.023	0.002	0.565	350474	1.916E-22	88.939	0.0003
PLT	Stroke	rs62252691	C	G	-0.036	0.002	0.373	350474	2.452E-47	209.229	0.0006
PLT	Stroke	rs6445826	C	T	0.104	0.002	0.499	350474	1E-200	1920.079	0.0054
PLT	Stroke	rs6505129	A	G	-0.068	0.002	0.517	350474	9.64E-192	817.864	0.0023
PLT	Stroke	rs6510152	T	C	0.019	0.002	0.474	350474	7.645E-17	65.468	0.0002
PLT	Stroke	rs655029	A	G	0.074	0.003	0.708	350474	1.83E-183	799.783	0.0023
PLT	Stroke	rs692502	A	G	0.092	0.002	0.558	350474	1E-200	1458.425	0.0041
PLT	Stroke	rs6993770	T	A	-0.066	0.003	0.286	350474	1.3E-147	626.335	0.0018
PLT	Stroke	rs7029729	A	C	-0.032	0.003	0.255	350474	4.089E-34	138.968	0.0004
PLT	Stroke	rs7032197	T	A	-0.029	0.003	0.652	350474	3.299E-29	133.985	0.0004
PLT	Stroke	rs7111926	A	C	-0.025	0.002	0.640	350474	7.471E-25	101.683	0.0003
PLT	Stroke	rs718515	A	G	-0.017	0.002	0.552	350474	1.069E-12	47.493	0.0001
PLT	Stroke	rs721412	G	A	-0.027	0.002	0.319	350474	5.605E-27	109.659	0.0003
PLT	Stroke	rs72798043	G	C	-0.032	0.004	0.086	350474	1.309E-14	55.920	0.0002
PLT	Stroke	rs72807421	T	C	-0.053	0.008	0.026	350474	2.283E-12	48.785	0.0001
PLT	Stroke	rs72828251	G	T	0.018	0.002	0.410	350474	9.273E-14	54.488	0.0002
PLT	Stroke	rs73078594	T	A	0.028	0.004	0.102	350474	1.33E-13	51.342	0.0001
PLT	Stroke	rs73185736	A	G	0.105	0.009	0.018	350474	1.541E-32	134.877	0.0004
PLT	Stroke	rs738390	T	A	-0.034	0.002	0.312	350474	7.525E-42	172.344	0.0005
PLT	Stroke	rs74505413	G	T	0.094	0.006	0.043	350474	1.101E-59	250.607	0.0007
PLT	Stroke	rs74821293	C	T	-0.046	0.004	0.110	350474	3.458E-35	144.016	0.0004
PLT	Stroke	rs75107793	A	G	0.107	0.004	0.073	350474	1.12E-127	548.037	0.0016
PLT	Stroke	rs754709	A	G	-0.016	0.002	0.403	350474	5.014E-11	40.913	0.0001
PLT	Stroke	rs7705526	A	C	0.038	0.002	0.325	350474	2.517E-52	222.193	0.0006
PLT	Stroke	rs7833924	G	A	0.041	0.002	0.430	350474	5.142E-69	288.458	0.0008
PLT	Stroke	rs7865719	G	A	-0.033	0.002	0.488	350474	1.301E-46	193.064	0.0006
PLT	Stroke	rs78909033	A	G	0.064	0.003	0.135	350474	7.285E-80	334.747	0.001
PLT	Stroke	rs7940646	C	T	0.024	0.002	0.690	350474	2.258E-21	83.918	0.0002
PLT	Stroke	rs79548680	C	G	-0.030	0.003	0.135	350474	2.557E-18	72.290	0.0002
PLT	Stroke	rs881586	C	T	0.046	0.003	0.140	350474	1.035E-42	175.873	0.0005
PLT	Stroke	rs9307477	A	G	-0.016	0.002	0.516	350474	4.07E-12	45.684	0.0001
PLT	Stroke	rs9399136	C	T	0.108	0.003	0.259	350474	1E-200	1575.678	0.0045
PLT	Stroke	rs9402633	T	C	0.033	0.003	0.213	350474	2.776E-31	127.003	0.0004
PLT	Stroke	rs9704457	G	A	-0.017	0.002	0.466	350474	1.215E-12	49.799	0.0001

Table S1. SNPs information of platelet indices (PLT, PDW, MPV and PCT) with Stroke and stroke subtypes group (ALS, LAS, SVS and CES)

Exposure	Outcome	SNP	Effect allele	Other allele	Beta	SE	Eaf	N	*P* val	Fval	R2
PLT	Stroke	rs9860749	A	G	-0.024	0.003	0.750	350474	8.335E-19	73.556	0.0002
PLT	AIS	rs10840160	C	T	-0.023	0.002	0.462	350474	6.361E-23	91.500	0.0003
PLT	AIS	rs11071720	C	T	0.038	0.003	0.702	350474	1.46E-51	213.858	0.0006
PLT	AIS	rs11082304	T	G	-0.052	0.002	0.514	350474	9.77E-113	475.721	0.0014
PLT	AIS	rs114694170	C	T	0.144	0.005	0.059	350474	1.11E-186	811.444	0.0023
PLT	AIS	rs115365432	G	T	-0.029	0.004	0.102	350474	6.425E-14	52.881	0.0002
PLT	AIS	rs11553699	G	A	-0.110	0.004	0.135	350474	1E-200	991.239	0.0028
PLT	AIS	rs11604127	T	C	0.092	0.003	0.232	350474	1E-200	1055.154	0.003
PLT	AIS	rs11731274	G	T	0.052	0.003	0.176	350474	1.504E-65	274.024	0.0008
PLT	AIS	rs11841319	T	C	-0.061	0.004	0.103	350474	1.136E-57	241.375	0.0007
PLT	AIS	rs12065863	C	T	-0.037	0.003	0.251	350474	1.947E-43	179.545	0.0005
PLT	AIS	rs12569998	G	T	0.020	0.003	0.158	350474	1.608E-10	38.279	0.0001
PLT	AIS	rs12666044	A	T	0.028	0.002	0.665	350474	2.234E-29	123.126	0.0004
PLT	AIS	rs12748176	C	T	0.056	0.005	0.056	350474	1.107E-28	115.285	0.0003
PLT	AIS	rs148762611	G	A	-0.019	0.002	0.347	350474	7.63E-15	56.840	0.0002
PLT	AIS	rs151234	C	G	0.065	0.003	0.129	350474	3.843E-78	328.655	0.0009
PLT	AIS	rs1558328	T	C	-0.052	0.002	0.497	350474	1.94E-111	473.834	0.0014
PLT	AIS	rs16978075	C	T	0.025	0.004	0.110	350474	1.269E-11	43.154	0.0001
PLT	AIS	rs17652304	G	C	-0.087	0.005	0.058	350474	1.49E-67	286.960	0.0008
PLT	AIS	rs1768584	G	A	0.051	0.002	0.386	350474	1.36E-102	432.078	0.0012
PLT	AIS	rs2027939	A	G	-0.031	0.003	0.142	350474	3.767E-20	79.915	0.0002
PLT	AIS	rs2075672	G	A	0.027	0.002	0.624	350474	2.083E-30	122.654	0.0003
PLT	AIS	rs2155380	G	A	0.054	0.003	0.271	350474	6.234E-94	397.056	0.0011
PLT	AIS	rs2236055	G	A	-0.038	0.002	0.468	350474	5.254E-61	253.695	0.0007
PLT	AIS	rs2297066	G	C	0.052	0.003	0.242	350474	2.568E-82	345.542	0.001
PLT	AIS	rs2535403	C	T	-0.021	0.002	0.555	350474	5.131E-19	74.731	0.0002
PLT	AIS	rs2631360	A	G	0.027	0.002	0.519	350474	1.398E-32	132.239	0.0004
PLT	AIS	rs2699425	C	T	0.021	0.002	0.373	350474	4.435E-19	75.673	0.0002
PLT	AIS	rs2724564	T	G	0.022	0.002	0.561	350474	7.695E-21	82.102	0.0002
PLT	AIS	rs2841102	C	T	0.019	0.002	0.439	350474	1.336E-16	64.074	0.0002
PLT	AIS	rs301369	G	A	0.041	0.002	0.410	350474	2.275E-67	280.804	0.0008
PLT	AIS	rs3205136	A	C	-0.036	0.004	0.094	350474	3.739E-19	76.858	0.0002
PLT	AIS	rs342293	G	C	-0.076	0.002	0.459	350474	1E-200	999.347	0.0028
PLT	AIS	rs346738	C	T	0.033	0.002	0.655	350474	5.378E-42	175.581	0.0005
PLT	AIS	rs346744	A	G	-0.022	0.002	0.582	350474	9.406E-21	82.809	0.0002
PLT	AIS	rs4148445	T	C	0.064	0.004	0.918	350474	3.74E-53	220.278	0.0006
PLT	AIS	rs4257458	G	A	0.023	0.002	0.390	350474	6.693E-22	87.597	0.0002
PLT	AIS	rs4272720	G	A	-0.031	0.003	0.237	350474	1.879E-30	124.514	0.0004
PLT	AIS	rs4309234	T	C	-0.035	0.003	0.781	350474	2.54E-35	145.072	0.0004
PLT	AIS	rs4592028	C	T	0.017	0.002	0.330	350474	6.121E-12	44.585	0.0001
PLT	AIS	rs479404	C	T	-0.018	0.003	0.270	350474	1.494E-11	42.930	0.0001
PLT	AIS	rs4818031	G	A	-0.018	0.002	0.321	350474	6.742E-13	48.980	0.0001
PLT	AIS	rs4820268	A	G	-0.025	0.002	0.537	350474	7.114E-27	107.544	0.0003
PLT	AIS	rs4871590	G	A	0.016	0.002	0.578	350474	2.112E-11	42.552	0.0001
PLT	AIS	rs4905994	C	T	0.036	0.002	0.683	350474	7.003E-48	199.107	0.0006
PLT	AIS	rs4906254	G	A	-0.031	0.002	0.347	350474	9.986E-37	150.438	0.0004

Table S1. SNPs information of platelet indices (PLT, PDW, MPV and PCT) with Stroke and stroke subtypes group (ALS, LAS, SVS and CES)

Exposure	Outcome	SNP	Effect allele	Other allele	Beta	SE	Eaf	N	*P* val	Fval	R2
PLT	AIS	rs4935968	T	C	-0.026	0.002	0.517	350474	6.992E-30	120.641	0.0003
PLT	AIS	rs511515	G	A	0.098	0.003	0.699	350474	1E-200	1422.493	0.004
PLT	AIS	rs55660207	G	C	0.058	0.005	0.071	350474	4.307E-38	156.098	0.0004
PLT	AIS	rs55941898	T	C	0.025	0.003	0.140	350474	5.4E-14	53.365	0.0002
PLT	AIS	rs56043070	A	G	-0.127	0.004	0.072	350474	3.24E-178	756.500	0.0022
PLT	AIS	rs57843631	T	C	-0.225	0.009	0.019	350474	1.44E-147	672.478	0.0019
PLT	AIS	rs6070696	G	A	0.051	0.003	0.186	350474	4.576E-66	277.495	0.0008
PLT	AIS	rs60757417	G	C	-0.081	0.005	0.060	350474	2.384E-61	260.984	0.0007
PLT	AIS	rs6119728	A	G	-0.030	0.003	0.291	350474	2.681E-31	126.551	0.0004
PLT	AIS	rs6141	T	C	0.056	0.002	0.530	350474	2.78E-126	542.840	0.0015
PLT	AIS	rs61869602	C	T	0.016	0.002	0.407	350474	3.015E-11	41.608	0.0001
PLT	AIS	rs61963261	T	C	0.022	0.003	0.159	350474	4.079E-12	44.979	0.0001
PLT	AIS	rs62048972	T	C	0.039	0.003	0.164	350474	1.221E-36	150.076	0.0004
PLT	AIS	rs62175229	T	C	-0.023	0.002	0.565	350474	1.916E-22	88.939	0.0003
PLT	AIS	rs62252691	C	G	-0.036	0.002	0.373	350474	2.452E-47	209.229	0.0006
PLT	AIS	rs6445826	C	T	0.104	0.002	0.499	350474	1E-200	1920.079	0.0054
PLT	AIS	rs6505129	A	G	-0.068	0.002	0.517	350474	9.64E-192	817.864	0.0023
PLT	AIS	rs6510152	T	C	0.019	0.002	0.474	350474	7.645E-17	65.468	0.0002
PLT	AIS	rs655029	A	G	0.074	0.003	0.708	350474	1.83E-183	799.783	0.0023
PLT	AIS	rs692502	A	G	0.092	0.002	0.558	350474	1E-200	1458.425	0.0041
PLT	AIS	rs6993770	T	A	-0.066	0.003	0.286	350474	1.3E-147	626.335	0.0018
PLT	AIS	rs7029729	A	C	-0.032	0.003	0.255	350474	4.089E-34	138.968	0.0004
PLT	AIS	rs7032197	T	A	-0.029	0.003	0.652	350474	3.299E-29	133.985	0.0004
PLT	AIS	rs7111926	A	C	-0.025	0.002	0.640	350474	7.471E-25	101.683	0.0003
PLT	AIS	rs718515	A	G	-0.017	0.002	0.552	350474	1.069E-12	47.493	0.0001
PLT	AIS	rs721412	G	A	-0.027	0.002	0.319	350474	5.605E-27	109.659	0.0003
PLT	AIS	rs72798043	G	C	-0.032	0.004	0.086	350474	1.309E-14	55.920	0.0002
PLT	AIS	rs72807421	T	C	-0.053	0.008	0.026	350474	2.283E-12	48.785	0.0001
PLT	AIS	rs72828251	G	T	0.018	0.002	0.410	350474	9.273E-14	54.488	0.0002
PLT	AIS	rs73078594	T	A	0.028	0.004	0.102	350474	1.33E-13	51.342	0.0001
PLT	AIS	rs73185736	A	G	0.105	0.009	0.018	350474	1.541E-32	134.877	0.0004
PLT	AIS	rs738390	T	A	-0.034	0.002	0.312	350474	7.525E-42	172.344	0.0005
PLT	AIS	rs74505413	G	T	0.094	0.006	0.043	350474	1.101E-59	250.607	0.0007
PLT	AIS	rs74821293	C	T	-0.046	0.004	0.110	350474	3.458E-35	144.016	0.0004
PLT	AIS	rs75107793	A	G	0.107	0.004	0.073	350474	1.12E-127	548.037	0.0016
PLT	AIS	rs754709	A	G	-0.016	0.002	0.403	350474	5.014E-11	40.913	0.0001
PLT	AIS	rs7705526	A	C	0.038	0.002	0.325	350474	2.517E-52	222.193	0.0006
PLT	AIS	rs7833924	G	A	0.041	0.002	0.430	350474	5.142E-69	288.458	0.0008
PLT	AIS	rs7865719	G	A	-0.033	0.002	0.488	350474	1.301E-46	193.064	0.0006
PLT	AIS	rs78909033	A	G	0.064	0.003	0.135	350474	7.285E-80	334.747	0.001
PLT	AIS	rs7940646	C	T	0.024	0.002	0.690	350474	2.258E-21	83.918	0.0002
PLT	AIS	rs79548680	C	G	-0.030	0.003	0.135	350474	2.557E-18	72.290	0.0002
PLT	AIS	rs881586	C	T	0.046	0.003	0.140	350474	1.035E-42	175.873	0.0005
PLT	AIS	rs9307477	A	G	-0.016	0.002	0.516	350474	4.07E-12	45.684	0.0001
PLT	AIS	rs9399136	C	T	0.108	0.003	0.259	350474	1E-200	1575.678	0.0045
PLT	AIS	rs9402633	T	C	0.033	0.003	0.213	350474	2.776E-31	127.003	0.0004

Table S1. SNPs information of platelet indices (PLT, PDW, MPV and PCT) with Stroke and stroke subtypes group (ALS, LAS, SVS and CES)

Exposure	Outcome	SNP	Effect allele	Other allele	Beta	SE	Eaf	N	*P* val	Fval	R2
PLT	AIS	rs9704457	G	A	-0.017	0.002	0.466	350474	1.215E-12	49.799	0.0001
PLT	AIS	rs9860749	A	G	-0.024	0.003	0.750	350474	8.335E-19	73.556	0.0002
PLT	LAS	rs10840160	C	T	-0.023	0.002	0.462	350474	6.361E-23	91.500	0.0003
PLT	LAS	rs11071720	C	T	0.038	0.003	0.702	350474	1.46E-51	213.858	0.0006
PLT	LAS	rs11082304	T	G	-0.052	0.002	0.514	350474	9.77E-113	475.721	0.0014
PLT	LAS	rs114694170	C	T	0.144	0.005	0.059	350474	1.11E-186	811.444	0.0023
PLT	LAS	rs115365432	G	T	-0.029	0.004	0.102	350474	6.425E-14	52.881	0.0002
PLT	LAS	rs11553699	G	A	-0.110	0.004	0.135	350474	1E-200	991.239	0.0028
PLT	LAS	rs11604127	T	C	0.092	0.003	0.232	350474	1E-200	1055.154	0.003
PLT	LAS	rs11731274	G	T	0.052	0.003	0.176	350474	1.504E-65	274.024	0.0008
PLT	LAS	rs11841319	T	C	-0.061	0.004	0.103	350474	1.136E-57	241.375	0.0007
PLT	LAS	rs12065863	C	T	-0.037	0.003	0.251	350474	1.947E-43	179.545	0.0005
PLT	LAS	rs12569998	G	T	0.020	0.003	0.158	350474	1.608E-10	38.279	0.0001
PLT	LAS	rs12666044	A	T	0.028	0.002	0.665	350474	2.234E-29	123.126	0.0004
PLT	LAS	rs12748176	C	T	0.056	0.005	0.056	350474	1.107E-28	115.285	0.0003
PLT	LAS	rs148762611	G	A	-0.019	0.002	0.347	350474	7.63E-15	56.840	0.0002
PLT	LAS	rs151234	C	G	0.065	0.003	0.129	350474	3.843E-78	328.655	0.0009
PLT	LAS	rs1558328	T	C	-0.052	0.002	0.497	350474	1.94E-111	473.834	0.0014
PLT	LAS	rs16978075	C	T	0.025	0.004	0.110	350474	1.269E-11	43.154	0.0001
PLT	LAS	rs17652304	G	C	-0.087	0.005	0.058	350474	1.49E-67	286.960	0.0008
PLT	LAS	rs1768584	G	A	0.051	0.002	0.386	350474	1.36E-102	432.078	0.0012
PLT	LAS	rs2027939	A	G	-0.031	0.003	0.142	350474	3.767E-20	79.915	0.0002
PLT	LAS	rs2075672	G	A	0.027	0.002	0.624	350474	2.083E-30	122.654	0.0003
PLT	LAS	rs2155380	G	A	0.054	0.003	0.271	350474	6.234E-94	397.056	0.0011
PLT	LAS	rs2236055	G	A	-0.038	0.002	0.468	350474	5.254E-61	253.695	0.0007
PLT	LAS	rs2297066	G	C	0.052	0.003	0.242	350474	2.568E-82	345.542	0.001
PLT	LAS	rs2535403	C	T	-0.021	0.002	0.555	350474	5.131E-19	74.731	0.0002
PLT	LAS	rs2631360	A	G	0.027	0.002	0.519	350474	1.398E-32	132.239	0.0004
PLT	LAS	rs2699425	C	T	0.021	0.002	0.373	350474	4.435E-19	75.673	0.0002
PLT	LAS	rs2724564	T	G	0.022	0.002	0.561	350474	7.695E-21	82.102	0.0002
PLT	LAS	rs2841102	C	T	0.019	0.002	0.439	350474	1.336E-16	64.074	0.0002
PLT	LAS	rs301369	G	A	0.041	0.002	0.410	350474	2.275E-67	280.804	0.0008
PLT	LAS	rs3205136	A	C	-0.036	0.004	0.094	350474	3.739E-19	76.858	0.0002
PLT	LAS	rs342293	G	C	-0.076	0.002	0.459	350474	1E-200	999.347	0.0028
PLT	LAS	rs346738	C	T	0.033	0.002	0.655	350474	5.378E-42	175.581	0.0005
PLT	LAS	rs346744	A	G	-0.022	0.002	0.582	350474	9.406E-21	82.809	0.0002
PLT	LAS	rs4148445	T	C	0.064	0.004	0.918	350474	3.74E-53	220.278	0.0006
PLT	LAS	rs4257458	G	A	0.023	0.002	0.390	350474	6.693E-22	87.597	0.0002
PLT	LAS	rs4272720	G	A	-0.031	0.003	0.237	350474	1.879E-30	124.514	0.0004
PLT	LAS	rs4309234	T	C	-0.035	0.003	0.781	350474	2.54E-35	145.072	0.0004
PLT	LAS	rs4592028	C	T	0.017	0.002	0.330	350474	6.121E-12	44.585	0.0001
PLT	LAS	rs479404	C	T	-0.018	0.003	0.270	350474	1.494E-11	42.930	0.0001
PLT	LAS	rs4818031	G	A	-0.018	0.002	0.321	350474	6.742E-13	48.980	0.0001
PLT	LAS	rs4820268	A	G	-0.025	0.002	0.537	350474	7.114E-27	107.544	0.0003
PLT	LAS	rs4871590	G	A	0.016	0.002	0.578	350474	2.112E-11	42.552	0.0001
PLT	LAS	rs4905994	C	T	0.036	0.002	0.683	350474	7.003E-48	199.107	0.0006

Table S1. SNPs information of platelet indices (PLT, PDW, MPV and PCT) with Stroke and stroke subtypes group (ALS, LAS, SVS and CES)

Exposure	Outcome	SNP	Effect allele	Other allele	Beta	SE	Eaf	N	*P* val	Fval	R2
PLT	LAS	rs4906254	G	A	-0.031	0.002	0.347	350474	9.986E-37	150.438	0.0004
PLT	LAS	rs4935968	T	C	-0.026	0.002	0.517	350474	6.992E-30	120.641	0.0003
PLT	LAS	rs511515	G	A	0.098	0.003	0.699	350474	1E-200	1422.493	0.004
PLT	LAS	rs55660207	G	C	0.058	0.005	0.071	350474	4.307E-38	156.098	0.0004
PLT	LAS	rs55941898	T	C	0.025	0.003	0.140	350474	5.4E-14	53.365	0.0002
PLT	LAS	rs56043070	A	G	-0.127	0.004	0.072	350474	3.24E-178	756.500	0.0022
PLT	LAS	rs57843631	T	C	-0.225	0.009	0.019	350474	1.44E-147	672.478	0.0019
PLT	LAS	rs6070696	G	A	0.051	0.003	0.186	350474	4.576E-66	277.495	0.0008
PLT	LAS	rs60757417	G	C	-0.081	0.005	0.060	350474	2.384E-61	260.984	0.0007
PLT	LAS	rs6119728	A	G	-0.030	0.003	0.291	350474	2.681E-31	126.551	0.0004
PLT	LAS	rs6141	T	C	0.056	0.002	0.530	350474	2.78E-126	542.840	0.0015
PLT	LAS	rs61869602	C	T	0.016	0.002	0.407	350474	3.015E-11	41.608	0.0001
PLT	LAS	rs61963261	T	C	0.022	0.003	0.159	350474	4.079E-12	44.979	0.0001
PLT	LAS	rs62048972	T	C	0.039	0.003	0.164	350474	1.221E-36	150.076	0.0004
PLT	LAS	rs62175229	T	C	-0.023	0.002	0.565	350474	1.916E-22	88.939	0.0003
PLT	LAS	rs62252691	C	G	-0.036	0.002	0.373	350474	2.452E-47	209.229	0.0006
PLT	LAS	rs6445826	C	T	0.104	0.002	0.499	350474	1E-200	1920.079	0.0054
PLT	LAS	rs6505129	A	G	-0.068	0.002	0.517	350474	9.64E-192	817.864	0.0023
PLT	LAS	rs6510152	T	C	0.019	0.002	0.474	350474	7.645E-17	65.468	0.0002
PLT	LAS	rs655029	A	G	0.074	0.003	0.708	350474	1.83E-183	799.783	0.0023
PLT	LAS	rs692502	A	G	0.092	0.002	0.558	350474	1E-200	1458.425	0.0041
PLT	LAS	rs6993770	T	A	-0.066	0.003	0.286	350474	1.3E-147	626.335	0.0018
PLT	LAS	rs7029729	A	C	-0.032	0.003	0.255	350474	4.089E-34	138.968	0.0004
PLT	LAS	rs7032197	T	A	-0.029	0.003	0.652	350474	3.299E-29	133.985	0.0004
PLT	LAS	rs7111926	A	C	-0.025	0.002	0.640	350474	7.471E-25	101.683	0.0003
PLT	LAS	rs718515	A	G	-0.017	0.002	0.552	350474	1.069E-12	47.493	0.0001
PLT	LAS	rs721412	G	A	-0.027	0.002	0.319	350474	5.605E-27	109.659	0.0003
PLT	LAS	rs72798043	G	C	-0.032	0.004	0.086	350474	1.309E-14	55.920	0.0002
PLT	LAS	rs72807421	T	C	-0.053	0.008	0.026	350474	2.283E-12	48.785	0.0001
PLT	LAS	rs72828251	G	T	0.018	0.002	0.410	350474	9.273E-14	54.488	0.0002
PLT	LAS	rs73078594	T	A	0.028	0.004	0.102	350474	1.33E-13	51.342	0.0001
PLT	LAS	rs73185736	A	G	0.105	0.009	0.018	350474	1.541E-32	134.877	0.0004
PLT	LAS	rs738390	T	A	-0.034	0.002	0.312	350474	7.525E-42	172.344	0.0005
PLT	LAS	rs74505413	G	T	0.094	0.006	0.043	350474	1.101E-59	250.607	0.0007
PLT	LAS	rs74821293	C	T	-0.046	0.004	0.110	350474	3.458E-35	144.016	0.0004
PLT	LAS	rs75107793	A	G	0.107	0.004	0.073	350474	1.12E-127	548.037	0.0016
PLT	LAS	rs754709	A	G	-0.016	0.002	0.403	350474	5.014E-11	40.913	0.0001
PLT	LAS	rs7705526	A	C	0.038	0.002	0.325	350474	2.517E-52	222.193	0.0006
PLT	LAS	rs7833924	G	A	0.041	0.002	0.430	350474	5.142E-69	288.458	0.0008
PLT	LAS	rs7865719	G	A	-0.033	0.002	0.488	350474	1.301E-46	193.064	0.0006
PLT	LAS	rs78909033	A	G	0.064	0.003	0.135	350474	7.285E-80	334.747	0.001
PLT	LAS	rs7940646	C	T	0.024	0.002	0.690	350474	2.258E-21	83.918	0.0002
PLT	LAS	rs79548680	C	G	-0.030	0.003	0.135	350474	2.557E-18	72.290	0.0002
PLT	LAS	rs881586	C	T	0.046	0.003	0.140	350474	1.035E-42	175.873	0.0005
PLT	LAS	rs9307477	A	G	-0.016	0.002	0.516	350474	4.07E-12	45.684	0.0001
PLT	LAS	rs9399136	C	T	0.108	0.003	0.259	350474	1E-200	1575.678	0.0045

Table S1. SNPs information of platelet indices (PLT, PDW, MPV and PCT) with Stroke and stroke subtypes group (ALS, LAS, SVS and CES)

Exposure	Outcome	SNP	Effect allele	Other allele	Beta	SE	Eaf	N	*P* val	Fval	R2
PLT	LAS	rs9402633	T	C	0.033	0.003	0.213	350474	2.776E-31	127.003	0.0004
PLT	LAS	rs9704457	G	A	-0.017	0.002	0.466	350474	1.215E-12	49.799	0.0001
PLT	LAS	rs9860749	A	G	-0.024	0.003	0.750	350474	8.335E-19	73.556	0.0002
PLT	SVS	rs10840160	C	T	-0.023	0.002	0.462	350474	6.361E-23	91.500	0.0003
PLT	SVS	rs11071720	C	T	0.038	0.003	0.702	350474	1.46E-51	213.858	0.0006
PLT	SVS	rs11082304	T	G	-0.052	0.002	0.514	350474	9.77E-113	475.721	0.0014
PLT	SVS	rs114694170	C	T	0.144	0.005	0.059	350474	1.11E-186	811.444	0.0023
PLT	SVS	rs115365432	G	T	-0.029	0.004	0.102	350474	6.425E-14	52.881	0.0002
PLT	SVS	rs11553699	G	A	-0.110	0.004	0.135	350474	1E-200	991.239	0.0028
PLT	SVS	rs11604127	T	C	0.092	0.003	0.232	350474	1E-200	1055.154	0.003
PLT	SVS	rs11731274	G	T	0.052	0.003	0.176	350474	1.504E-65	274.024	0.0008
PLT	SVS	rs11841319	T	C	-0.061	0.004	0.103	350474	1.136E-57	241.375	0.0007
PLT	SVS	rs12065863	C	T	-0.037	0.003	0.251	350474	1.947E-43	179.545	0.0005
PLT	SVS	rs12569998	G	T	0.020	0.003	0.158	350474	1.608E-10	38.279	0.0001
PLT	SVS	rs12666044	A	T	0.028	0.002	0.665	350474	2.234E-29	123.126	0.0004
PLT	SVS	rs12748176	C	T	0.056	0.005	0.056	350474	1.107E-28	115.285	0.0003
PLT	SVS	rs148762611	G	A	-0.019	0.002	0.347	350474	7.63E-15	56.840	0.0002
PLT	SVS	rs151234	C	G	0.065	0.003	0.129	350474	3.843E-78	328.655	0.0009
PLT	SVS	rs1558328	T	C	-0.052	0.002	0.497	350474	1.94E-111	473.834	0.0014
PLT	SVS	rs16978075	C	T	0.025	0.004	0.110	350474	1.269E-11	43.154	0.0001
PLT	SVS	rs17652304	G	C	-0.087	0.005	0.058	350474	1.49E-67	286.960	0.0008
PLT	SVS	rs1768584	G	A	0.051	0.002	0.386	350474	1.36E-102	432.078	0.0012
PLT	SVS	rs2027939	A	G	-0.031	0.003	0.142	350474	3.767E-20	79.915	0.0002
PLT	SVS	rs2075672	G	A	0.027	0.002	0.624	350474	2.083E-30	122.654	0.0003
PLT	SVS	rs2155380	G	A	0.054	0.003	0.271	350474	6.234E-94	397.056	0.0011
PLT	SVS	rs2236055	G	A	-0.038	0.002	0.468	350474	5.254E-61	253.695	0.0007
PLT	SVS	rs2297066	G	C	0.052	0.003	0.242	350474	2.568E-82	345.542	0.001
PLT	SVS	rs2535403	C	T	-0.021	0.002	0.555	350474	5.131E-19	74.731	0.0002
PLT	SVS	rs2631360	A	G	0.027	0.002	0.519	350474	1.398E-32	132.239	0.0004
PLT	SVS	rs2699425	C	T	0.021	0.002	0.373	350474	4.435E-19	75.673	0.0002
PLT	SVS	rs2724564	T	G	0.022	0.002	0.561	350474	7.695E-21	82.102	0.0002
PLT	SVS	rs2841102	C	T	0.019	0.002	0.439	350474	1.336E-16	64.074	0.0002
PLT	SVS	rs301369	G	A	0.041	0.002	0.410	350474	2.275E-67	280.804	0.0008
PLT	SVS	rs3205136	A	C	-0.036	0.004	0.094	350474	3.739E-19	76.858	0.0002
PLT	SVS	rs342293	G	C	-0.076	0.002	0.459	350474	1E-200	999.347	0.0028
PLT	SVS	rs346738	C	T	0.033	0.002	0.655	350474	5.378E-42	175.581	0.0005
PLT	SVS	rs346744	A	G	-0.022	0.002	0.582	350474	9.406E-21	82.809	0.0002
PLT	SVS	rs4148445	T	C	0.064	0.004	0.918	350474	3.74E-53	220.278	0.0006
PLT	SVS	rs4257458	G	A	0.023	0.002	0.390	350474	6.693E-22	87.597	0.0002
PLT	SVS	rs4272720	G	A	-0.031	0.003	0.237	350474	1.879E-30	124.514	0.0004
PLT	SVS	rs4309234	T	C	-0.035	0.003	0.781	350474	2.54E-35	145.072	0.0004
PLT	SVS	rs4592028	C	T	0.017	0.002	0.330	350474	6.121E-12	44.585	0.0001
PLT	SVS	rs479404	C	T	-0.018	0.003	0.270	350474	1.494E-11	42.930	0.0001
PLT	SVS	rs4818031	G	A	-0.018	0.002	0.321	350474	6.742E-13	48.980	0.0001
PLT	SVS	rs4820268	A	G	-0.025	0.002	0.537	350474	7.114E-27	107.544	0.0003
PLT	SVS	rs4871590	G	A	0.016	0.002	0.578	350474	2.112E-11	42.552	0.0001

Table S1. SNPs information of platelet indices (PLT, PDW, MPV and PCT) with Stroke and stroke subtypes group (ALS, LAS, SVS and CES)

Exposure	Outcome	SNP	Effect allele	Other allele	Beta	SE	Eaf	N	*P* val	Fval	R2
PLT	SVS	rs4905994	C	T	0.036	0.002	0.683	350474	7.003E-48	199.107	0.0006
PLT	SVS	rs4906254	G	A	-0.031	0.002	0.347	350474	9.986E-37	150.438	0.0004
PLT	SVS	rs4935968	T	C	-0.026	0.002	0.517	350474	6.992E-30	120.641	0.0003
PLT	SVS	rs511515	G	A	0.098	0.003	0.699	350474	1E-200	1422.493	0.004
PLT	SVS	rs55660207	G	C	0.058	0.005	0.071	350474	4.307E-38	156.098	0.0004
PLT	SVS	rs55941898	T	C	0.025	0.003	0.140	350474	5.4E-14	53.365	0.0002
PLT	SVS	rs56043070	A	G	-0.127	0.004	0.072	350474	3.24E-178	756.500	0.0022
PLT	SVS	rs57843631	T	C	-0.225	0.009	0.019	350474	1.44E-147	672.478	0.0019
PLT	SVS	rs6070696	G	A	0.051	0.003	0.186	350474	4.576E-66	277.495	0.0008
PLT	SVS	rs60757417	G	C	-0.081	0.005	0.060	350474	2.384E-61	260.984	0.0007
PLT	SVS	rs6119728	A	G	-0.030	0.003	0.291	350474	2.681E-31	126.551	0.0004
PLT	SVS	rs6141	T	C	0.056	0.002	0.530	350474	2.78E-126	542.840	0.0015
PLT	SVS	rs61869602	C	T	0.016	0.002	0.407	350474	3.015E-11	41.608	0.0001
PLT	SVS	rs61963261	T	C	0.022	0.003	0.159	350474	4.079E-12	44.979	0.0001
PLT	SVS	rs62048972	T	C	0.039	0.003	0.164	350474	1.221E-36	150.076	0.0004
PLT	SVS	rs62175229	T	C	-0.023	0.002	0.565	350474	1.916E-22	88.939	0.0003
PLT	SVS	rs62252691	C	G	-0.036	0.002	0.373	350474	2.452E-47	209.229	0.0006
PLT	SVS	rs6445826	C	T	0.104	0.002	0.499	350474	1E-200	1920.079	0.0054
PLT	SVS	rs6505129	A	G	-0.068	0.002	0.517	350474	9.64E-192	817.864	0.0023
PLT	SVS	rs6510152	T	C	0.019	0.002	0.474	350474	7.645E-17	65.468	0.0002
PLT	SVS	rs655029	A	G	0.074	0.003	0.708	350474	1.83E-183	799.783	0.0023
PLT	SVS	rs692502	A	G	0.092	0.002	0.558	350474	1E-200	1458.425	0.0041
PLT	SVS	rs6993770	T	A	-0.066	0.003	0.286	350474	1.3E-147	626.335	0.0018
PLT	SVS	rs7029729	A	C	-0.032	0.003	0.255	350474	4.089E-34	138.968	0.0004
PLT	SVS	rs7032197	T	A	-0.029	0.003	0.652	350474	3.299E-29	133.985	0.0004
PLT	SVS	rs7111926	A	C	-0.025	0.002	0.640	350474	7.471E-25	101.683	0.0003
PLT	SVS	rs718515	A	G	-0.017	0.002	0.552	350474	1.069E-12	47.493	0.0001
PLT	SVS	rs721412	G	A	-0.027	0.002	0.319	350474	5.605E-27	109.659	0.0003
PLT	SVS	rs72798043	G	C	-0.032	0.004	0.086	350474	1.309E-14	55.920	0.0002
PLT	SVS	rs72807421	T	C	-0.053	0.008	0.026	350474	2.283E-12	48.785	0.0001
PLT	SVS	rs72828251	G	T	0.018	0.002	0.410	350474	9.273E-14	54.488	0.0002
PLT	SVS	rs73078594	T	A	0.028	0.004	0.102	350474	1.33E-13	51.342	0.0001
PLT	SVS	rs7310615	G	C	-0.095	0.002	0.518	350474	1E-200	1596.020	0.0045
PLT	SVS	rs73185736	A	G	0.105	0.009	0.018	350474	1.541E-32	134.877	0.0004
PLT	SVS	rs738390	T	A	-0.034	0.002	0.312	350474	7.525E-42	172.344	0.0005
PLT	SVS	rs74505413	G	T	0.094	0.006	0.043	350474	1.101E-59	250.607	0.0007
PLT	SVS	rs74821293	C	T	-0.046	0.004	0.110	350474	3.458E-35	144.016	0.0004
PLT	SVS	rs75107793	A	G	0.107	0.004	0.073	350474	1.12E-127	548.037	0.0016
PLT	SVS	rs754709	A	G	-0.016	0.002	0.403	350474	5.014E-11	40.913	0.0001
PLT	SVS	rs7705526	A	C	0.038	0.002	0.325	350474	2.517E-52	222.193	0.0006
PLT	SVS	rs7833924	G	A	0.041	0.002	0.430	350474	5.142E-69	288.458	0.0008
PLT	SVS	rs7865719	G	A	-0.033	0.002	0.488	350474	1.301E-46	193.064	0.0006
PLT	SVS	rs78909033	A	G	0.064	0.003	0.135	350474	7.285E-80	334.747	0.001
PLT	SVS	rs7940646	C	T	0.024	0.002	0.690	350474	2.258E-21	83.918	0.0002
PLT	SVS	rs79548680	C	G	-0.030	0.003	0.135	350474	2.557E-18	72.290	0.0002
PLT	SVS	rs881586	C	T	0.046	0.003	0.140	350474	1.035E-42	175.873	0.0005

Table S1. SNPs information of platelet indices (PLT, PDW, MPV and PCT) with Stroke and stroke subtypes group (ALS, LAS, SVS and CES)

Exposure	Outcome	SNP	Effect allele	Other allele	Beta	SE	Eaf	N	*P* val	Fval	R2
PLT	SVS	rs9307477	A	G	-0.016	0.002	0.516	350474	4.07E-12	45.684	0.0001
PLT	SVS	rs9399136	C	T	0.108	0.003	0.259	350474	1E-200	1575.678	0.0045
PLT	SVS	rs9402633	T	C	0.033	0.003	0.213	350474	2.776E-31	127.003	0.0004
PLT	SVS	rs9704457	G	A	-0.017	0.002	0.466	350474	1.215E-12	49.799	0.0001
PLT	SVS	rs9860749	A	G	-0.024	0.003	0.750	350474	8.335E-19	73.556	0.0002
PLT	CES	rs10840160	C	T	-0.023	0.002	0.462	350474	6.361E-23	91.500	0.0003
PLT	CES	rs11071720	C	T	0.038	0.003	0.702	350474	1.46E-51	213.858	0.0006
PLT	CES	rs11082304	T	G	-0.052	0.002	0.514	350474	9.77E-113	475.721	0.0014
PLT	CES	rs114694170	C	T	0.144	0.005	0.059	350474	1.11E-186	811.444	0.0023
PLT	CES	rs115365432	G	T	-0.029	0.004	0.102	350474	6.425E-14	52.881	0.0002
PLT	CES	rs11553699	G	A	-0.110	0.004	0.135	350474	1E-200	991.239	0.0028
PLT	CES	rs11604127	T	C	0.092	0.003	0.232	350474	1E-200	1055.154	0.003
PLT	CES	rs11731274	G	T	0.052	0.003	0.176	350474	1.504E-65	274.024	0.0008
PLT	CES	rs11841319	T	C	-0.061	0.004	0.103	350474	1.136E-57	241.375	0.0007
PLT	CES	rs12065863	C	T	-0.037	0.003	0.251	350474	1.947E-43	179.545	0.0005
PLT	CES	rs12569998	G	T	0.020	0.003	0.158	350474	1.608E-10	38.279	0.0001
PLT	CES	rs12666044	A	T	0.028	0.002	0.665	350474	2.234E-29	123.126	0.0004
PLT	CES	rs12748176	C	T	0.056	0.005	0.056	350474	1.107E-28	115.285	0.0003
PLT	CES	rs148762611	G	A	-0.019	0.002	0.347	350474	7.63E-15	56.840	0.0002
PLT	CES	rs151234	C	G	0.065	0.003	0.129	350474	3.843E-78	328.655	0.0009
PLT	CES	rs1558328	T	C	-0.052	0.002	0.497	350474	1.94E-111	473.834	0.0014
PLT	CES	rs16978075	C	T	0.025	0.004	0.110	350474	1.269E-11	43.154	0.0001
PLT	CES	rs17652304	G	C	-0.087	0.005	0.058	350474	1.49E-67	286.960	0.0008
PLT	CES	rs1768584	G	A	0.051	0.002	0.386	350474	1.36E-102	432.078	0.0012
PLT	CES	rs2027939	A	G	-0.031	0.003	0.142	350474	3.767E-20	79.915	0.0002
PLT	CES	rs2075672	G	A	0.027	0.002	0.624	350474	2.083E-30	122.654	0.0003
PLT	CES	rs2155380	G	A	0.054	0.003	0.271	350474	6.234E-94	397.056	0.0011
PLT	CES	rs2236055	G	A	-0.038	0.002	0.468	350474	5.254E-61	253.695	0.0007
PLT	CES	rs2297066	G	C	0.052	0.003	0.242	350474	2.568E-82	345.542	0.001
PLT	CES	rs2535403	C	T	-0.021	0.002	0.555	350474	5.131E-19	74.731	0.0002
PLT	CES	rs2631360	A	G	0.027	0.002	0.519	350474	1.398E-32	132.239	0.0004
PLT	CES	rs2699425	C	T	0.021	0.002	0.373	350474	4.435E-19	75.673	0.0002
PLT	CES	rs2724564	T	G	0.022	0.002	0.561	350474	7.695E-21	82.102	0.0002
PLT	CES	rs2841102	C	T	0.019	0.002	0.439	350474	1.336E-16	64.074	0.0002
PLT	CES	rs301369	G	A	0.041	0.002	0.410	350474	2.275E-67	280.804	0.0008
PLT	CES	rs3205136	A	C	-0.036	0.004	0.094	350474	3.739E-19	76.858	0.0002
PLT	CES	rs342293	G	C	-0.076	0.002	0.459	350474	1E-200	999.347	0.0028
PLT	CES	rs346738	C	T	0.033	0.002	0.655	350474	5.378E-42	175.581	0.0005
PLT	CES	rs346744	A	G	-0.022	0.002	0.582	350474	9.406E-21	82.809	0.0002
PLT	CES	rs4148445	T	C	0.064	0.004	0.918	350474	3.74E-53	220.278	0.0006
PLT	CES	rs4257458	G	A	0.023	0.002	0.390	350474	6.693E-22	87.597	0.0002
PLT	CES	rs4272720	G	A	-0.031	0.003	0.237	350474	1.879E-30	124.514	0.0004
PLT	CES	rs4309234	T	C	-0.035	0.003	0.781	350474	2.54E-35	145.072	0.0004
PLT	CES	rs4592028	C	T	0.017	0.002	0.330	350474	6.121E-12	44.585	0.0001
PLT	CES	rs479404	C	T	-0.018	0.003	0.270	350474	1.494E-11	42.930	0.0001
PLT	CES	rs4818031	G	A	-0.018	0.002	0.321	350474	6.742E-13	48.980	0.0001

Table S1. SNPs information of platelet indices (PLT, PDW, MPV and PCT) with Stroke and stroke subtypes group (ALS, LAS, SVS and CES)

Exposure	Outcome	SNP	Effect allele	Other allele	Beta	SE	Eaf	N	*P* val	Fval	R2
PLT	CES	rs4820268	A	G	-0.025	0.002	0.537	350474	7.114E-27	107.544	0.0003
PLT	CES	rs4871590	G	A	0.016	0.002	0.578	350474	2.112E-11	42.552	0.0001
PLT	CES	rs4905994	C	T	0.036	0.002	0.683	350474	7.003E-48	199.107	0.0006
PLT	CES	rs4906254	G	A	-0.031	0.002	0.347	350474	9.986E-37	150.438	0.0004
PLT	CES	rs4935968	T	C	-0.026	0.002	0.517	350474	6.992E-30	120.641	0.0003
PLT	CES	rs511515	G	A	0.098	0.003	0.699	350474	1E-200	1422.493	0.004
PLT	CES	rs55660207	G	C	0.058	0.005	0.071	350474	4.307E-38	156.098	0.0004
PLT	CES	rs55941898	T	C	0.025	0.003	0.140	350474	5.4E-14	53.365	0.0002
PLT	CES	rs56043070	A	G	-0.127	0.004	0.072	350474	3.24E-178	756.500	0.0022
PLT	CES	rs57843631	T	C	-0.225	0.009	0.019	350474	1.44E-147	672.478	0.0019
PLT	CES	rs6070696	G	A	0.051	0.003	0.186	350474	4.576E-66	277.495	0.0008
PLT	CES	rs60757417	G	C	-0.081	0.005	0.060	350474	2.384E-61	260.984	0.0007
PLT	CES	rs6119728	A	G	-0.030	0.003	0.291	350474	2.681E-31	126.551	0.0004
PLT	CES	rs6141	T	C	0.056	0.002	0.530	350474	2.78E-126	542.840	0.0015
PLT	CES	rs61869602	C	T	0.016	0.002	0.407	350474	3.015E-11	41.608	0.0001
PLT	CES	rs61963261	T	C	0.022	0.003	0.159	350474	4.079E-12	44.979	0.0001
PLT	CES	rs62048972	T	C	0.039	0.003	0.164	350474	1.221E-36	150.076	0.0004
PLT	CES	rs62175229	T	C	-0.023	0.002	0.565	350474	1.916E-22	88.939	0.0003
PLT	CES	rs62252691	C	G	-0.036	0.002	0.373	350474	2.452E-47	209.229	0.0006
PLT	CES	rs6445826	C	T	0.104	0.002	0.499	350474	1E-200	1920.079	0.0054
PLT	CES	rs6505129	A	G	-0.068	0.002	0.517	350474	9.64E-192	817.864	0.0023
PLT	CES	rs6510152	T	C	0.019	0.002	0.474	350474	7.645E-17	65.468	0.0002
PLT	CES	rs655029	A	G	0.074	0.003	0.708	350474	1.83E-183	799.783	0.0023
PLT	CES	rs692502	A	G	0.092	0.002	0.558	350474	1E-200	1458.425	0.0041
PLT	CES	rs6993770	T	A	-0.066	0.003	0.286	350474	1.3E-147	626.335	0.0018
PLT	CES	rs7029729	A	C	-0.032	0.003	0.255	350474	4.089E-34	138.968	0.0004
PLT	CES	rs7032197	T	A	-0.029	0.003	0.652	350474	3.299E-29	133.985	0.0004
PLT	CES	rs7111926	A	C	-0.025	0.002	0.640	350474	7.471E-25	101.683	0.0003
PLT	CES	rs718515	A	G	-0.017	0.002	0.552	350474	1.069E-12	47.493	0.0001
PLT	CES	rs721412	G	A	-0.027	0.002	0.319	350474	5.605E-27	109.659	0.0003
PLT	CES	rs72798043	G	C	-0.032	0.004	0.086	350474	1.309E-14	55.920	0.0002
PLT	CES	rs72807421	T	C	-0.053	0.008	0.026	350474	2.283E-12	48.785	0.0001
PLT	CES	rs72828251	G	T	0.018	0.002	0.410	350474	9.273E-14	54.488	0.0002
PLT	CES	rs73078594	T	A	0.028	0.004	0.102	350474	1.33E-13	51.342	0.0001
PLT	CES	rs7310615	G	C	-0.095	0.002	0.518	350474	1E-200	1596.020	0.0045
PLT	CES	rs73185736	A	G	0.105	0.009	0.018	350474	1.541E-32	134.877	0.0004
PLT	CES	rs738390	T	A	-0.034	0.002	0.312	350474	7.525E-42	172.344	0.0005
PLT	CES	rs74505413	G	T	0.094	0.006	0.043	350474	1.101E-59	250.607	0.0007
PLT	CES	rs74821293	C	T	-0.046	0.004	0.110	350474	3.458E-35	144.016	0.0004
PLT	CES	rs75107793	A	G	0.107	0.004	0.073	350474	1.12E-127	548.037	0.0016
PLT	CES	rs754709	A	G	-0.016	0.002	0.403	350474	5.014E-11	40.913	0.0001
PLT	CES	rs7705526	A	C	0.038	0.002	0.325	350474	2.517E-52	222.193	0.0006
PLT	CES	rs7833924	G	A	0.041	0.002	0.430	350474	5.142E-69	288.458	0.0008
PLT	CES	rs7865719	G	A	-0.033	0.002	0.488	350474	1.301E-46	193.064	0.0006
PLT	CES	rs7940646	C	T	0.024	0.002	0.690	350474	2.258E-21	83.918	0.0002
PLT	CES	rs79548680	C	G	-0.030	0.003	0.135	350474	2.557E-18	72.290	0.0002

Table S1. SNPs information of platelet indices (PLT, PDW, MPV and PCT) with Stroke and stroke subtypes group (ALS, LAS, SVS and CES)

Exposure	Outcome	SNP	Effect allele	Other allele	Beta	SE	Eaf	N	*P* val	Fval	R2
PLT	CES	rs881586	C	T	0.046	0.003	0.140	350474	1.035E-42	175.873	0.0005
PLT	CES	rs9307477	A	G	-0.016	0.002	0.516	350474	4.07E-12	45.684	0.0001
PLT	CES	rs9399136	C	T	0.108	0.003	0.259	350474	1E-200	1575.678	0.0045
PLT	CES	rs9402633	T	C	0.033	0.003	0.213	350474	2.776E-31	127.003	0.0004
PLT	CES	rs9704457	G	A	-0.017	0.002	0.466	350474	1.215E-12	49.799	0.0001
PLT	CES	rs9860749	A	G	-0.024	0.003	0.750	350474	8.335E-19	73.556	0.0002
PCT	Stroke	rs10411361	G	A	0.017	0.002	0.484	350471	6.616E-14	50.931	0.0001
PCT	Stroke	rs10779836	T	C	0.021	0.003	0.803	350471	3.856E-13	48.015	0.0001
PCT	Stroke	rs10849334	G	A	-0.016	0.003	0.276	350471	2.065E-10	36.721	0.0001
PCT	Stroke	rs10931934	C	T	0.018	0.002	0.608	350471	4.929E-14	51.482	0.0001
PCT	Stroke	rs114694170	C	T	0.081	0.005	0.059	350471	3.921E-62	255.373	0.0007
PCT	Stroke	rs11553699	G	A	0.112	0.003	0.135	350471	1E-200	1041.016	0.003
PCT	Stroke	rs116234817	A	G	0.082	0.005	0.052	350471	1.297E-57	231.528	0.0007
PCT	Stroke	rs11906768	C	T	-0.075	0.003	0.266	350471	8.09E-190	781.155	0.0022
PCT	Stroke	rs12052715	G	C	-0.043	0.003	0.727	350471	1.297E-64	259.926	0.0007
PCT	Stroke	rs12155039	A	C	-0.047	0.002	0.435	350471	6.153E-92	374.063	0.0011
PCT	Stroke	rs12340895	G	C	-0.023	0.003	0.261	350471	1.267E-18	70.096	0.0002
PCT	Stroke	rs12412214	A	G	-0.026	0.003	0.284	350471	1.467E-25	98.775	0.0003
PCT	Stroke	rs12491937	G	A	-0.037	0.002	0.418	350471	4.086E-57	229.320	0.0007
PCT	Stroke	rs12882460	T	C	0.042	0.005	0.066	350471	3.314E-20	76.980	0.0002
PCT	Stroke	rs12943566	G	A	0.035	0.002	0.654	350471	6.294E-48	191.297	0.0005
PCT	Stroke	rs12947592	T	C	0.039	0.003	0.176	350471	3.263E-39	154.658	0.0004
PCT	Stroke	rs139974673	C	T	0.107	0.007	0.025	350471	2.455E-48	193.827	0.0006
PCT	Stroke	rs144344009	C	T	-0.060	0.005	0.065	350471	1.39E-37	154.130	0.0004
PCT	Stroke	rs148762611	G	A	-0.022	0.002	0.347	350471	8.102E-20	75.482	0.0002
PCT	Stroke	rs149290349	A	G	-0.066	0.004	0.075	350471	1.972E-52	214.809	0.0006
PCT	Stroke	rs151234	C	G	0.038	0.003	0.129	350471	3.068E-29	114.263	0.0003
PCT	Stroke	rs1555405	A	G	-0.061	0.003	0.248	350471	1.14E-119	491.621	0.0014
PCT	Stroke	rs16853270	A	C	0.052	0.004	0.089	350471	1.534E-38	152.407	0.0004
PCT	Stroke	rs17356664	T	C	-0.030	0.002	0.307	350471	4.485E-33	130.145	0.0004
PCT	Stroke	rs1736144	C	G	-0.022	0.002	0.429	350471	2.408E-21	81.059	0.0002
PCT	Stroke	rs182268522	G	A	-0.069	0.009	0.017	350471	6.461E-15	56.501	0.0002
PCT	Stroke	rs1984021	A	G	0.042	0.003	0.136	350471	2.759E-37	147.513	0.0004
PCT	Stroke	rs210134	G	A	0.098	0.002	0.688	350471	1E-200	1466.176	0.0042
PCT	Stroke	rs2155380	G	A	0.056	0.003	0.271	350471	4.9E-108	442.624	0.0013
PCT	Stroke	rs2236055	G	A	-0.047	0.002	0.468	350471	4.215E-93	378.343	0.0011
PCT	Stroke	rs2298993	A	G	-0.028	0.002	0.402	350471	1.522E-33	132.907	0.0004
PCT	Stroke	rs2327528	A	G	0.043	0.003	0.212	350471	1.113E-52	211.357	0.0006
PCT	Stroke	rs2327614	T	A	-0.015	0.002	0.543	350471	2.916E-11	40.148	0.0001
PCT	Stroke	rs2411229	C	T	-0.020	0.002	0.389	350471	2.241E-17	65.271	0.0002
PCT	Stroke	rs2469434	C	T	-0.023	0.002	0.406	350471	1.385E-22	86.904	0.0002
PCT	Stroke	rs2699425	C	T	0.017	0.002	0.373	350471	2.03E-13	49.553	0.0001
PCT	Stroke	rs272905	C	T	0.020	0.002	0.460	350471	2.387E-18	72.718	0.0002
PCT	Stroke	rs2738744	G	A	0.018	0.003	0.737	350471	1.473E-12	45.465	0.0001
PCT	Stroke	rs2834322	A	G	0.019	0.003	0.181	350471	5.184E-11	39.066	0.0001
PCT	Stroke	rs28505677	G	C	-0.037	0.003	0.237	350471	4.99E-42	169.240	0.0005

Table S1. SNPs information of platelet indices (PLT, PDW, MPV and PCT) with Stroke and stroke subtypes group (ALS, LAS, SVS and CES)

Exposure	Outcome	SNP	Effect allele	Other allele	Beta	SE	Eaf	N	*P* val	Fval	R2
PCT	Stroke	rs2894602	G	A	-0.030	0.003	0.764	350471	5.421E-28	110.730	0.0003
PCT	Stroke	rs2993488	T	C	-0.036	0.003	0.187	350471	1.746E-35	140.055	0.0004
PCT	Stroke	rs351372	A	T	-0.018	0.002	0.525	350471	1.035E-15	58.327	0.0002
PCT	Stroke	rs35478863	G	T	0.022	0.002	0.325	350471	1.385E-18	72.921	0.0002
PCT	Stroke	rs3860612	G	C	0.021	0.003	0.187	350471	9.33E-13	46.710	0.0001
PCT	Stroke	rs387582	A	C	0.095	0.002	0.504	350471	1E-200	1574.563	0.0045
PCT	Stroke	rs3956480	T	C	-0.016	0.002	0.465	350471	2.003E-11	44.889	0.0001
PCT	Stroke	rs4020660	A	G	-0.025	0.002	0.465	350471	6.784E-27	109.354	0.0003
PCT	Stroke	rs415064	C	G	-0.152	0.005	0.047	350471	6.25E-177	726.893	0.0021
PCT	Stroke	rs448355	C	T	0.020	0.002	0.613	350471	2.591E-17	65.101	0.0002
PCT	Stroke	rs449454	G	A	0.035	0.002	0.616	350471	9.995E-51	203.439	0.0006
PCT	Stroke	rs4773860	T	C	0.038	0.002	0.522	350471	1.509E-60	246.173	0.0007
PCT	Stroke	rs4800148	A	G	-0.019	0.003	0.782	350471	1.359E-12	45.296	0.0001
PCT	Stroke	rs4820268	A	G	-0.025	0.002	0.537	350471	3.256E-28	109.465	0.0003
PCT	Stroke	rs55966801	C	T	0.039	0.003	0.231	350471	8.494E-47	190.904	0.0005
PCT	Stroke	rs56043070	A	G	-0.077	0.004	0.072	350471	4.862E-69	278.140	0.0008
PCT	Stroke	rs57749886	T	C	0.025	0.002	0.649	350471	3.935E-26	101.190	0.0003
PCT	Stroke	rs58434384	G	A	0.060	0.004	0.086	350471	7.501E-49	195.725	0.0006
PCT	Stroke	rs59018815	A	G	-0.027	0.003	0.199	350471	5.242E-22	84.367	0.0002
PCT	Stroke	rs59865663	A	G	0.036	0.003	0.203	350471	1.792E-36	146.400	0.0004
PCT	Stroke	rs61750929	T	C	-0.084	0.005	0.055	350471	9.326E-64	258.375	0.0007
PCT	Stroke	rs654868	G	A	-0.018	0.003	0.745	350471	3.374E-12	43.898	0.0001
PCT	Stroke	rs6815294	A	G	0.026	0.002	0.573	350471	7.124E-29	113.043	0.0003
PCT	Stroke	rs6993770	T	A	-0.050	0.003	0.286	350471	1.669E-88	359.560	0.001
PCT	Stroke	rs703059	A	G	-0.018	0.002	0.483	350471	7.989E-15	55.827	0.0002
PCT	Stroke	rs7036656	T	C	0.048	0.003	0.722	350471	3.64E-79	322.052	0.0009
PCT	Stroke	rs71524003	C	G	0.026	0.003	0.290	350471	1.125E-24	96.560	0.0003
PCT	Stroke	rs724322	T	C	-0.017	0.002	0.423	350471	1.116E-13	50.090	0.0001
PCT	Stroke	rs73721606	T	C	-0.029	0.003	0.188	350471	7.178E-23	87.663	0.0003
PCT	Stroke	rs7413585	C	T	-0.017	0.002	0.517	350471	4.924E-14	52.410	0.0001
PCT	Stroke	rs75107793	A	G	0.106	0.004	0.073	350471	8.67E-130	538.573	0.0015
PCT	Stroke	rs7536036	C	T	0.022	0.002	0.342	350471	3.037E-20	78.263	0.0002
PCT	Stroke	rs7615916	A	G	0.020	0.003	0.248	350471	3.395E-14	52.309	0.0001
PCT	Stroke	rs7636889	A	G	0.024	0.003	0.762	350471	9.324E-19	71.941	0.0002
PCT	Stroke	rs76486546	T	C	0.023	0.003	0.148	350471	1.069E-12	45.802	0.0001
PCT	Stroke	rs7705526	A	C	0.051	0.002	0.325	350471	1.17E-97	407.741	0.0012
PCT	Stroke	rs77261872	T	C	0.047	0.003	0.126	350471	1.222E-42	170.727	0.0005
PCT	Stroke	rs7762253	T	C	0.016	0.002	0.452	350471	2.35E-12	44.561	0.0001
PCT	Stroke	rs7804205	A	G	-0.026	0.004	0.115	350471	4.677E-13	47.164	0.0001
PCT	Stroke	rs7833924	G	A	0.029	0.002	0.430	350471	9.489E-38	149.121	0.0004
PCT	Stroke	rs78565404	T	C	0.134	0.005	0.053	350471	1.8E-148	634.500	0.0018
PCT	Stroke	rs7943063	A	C	0.030	0.002	0.409	350471	1.696E-38	152.715	0.0004
PCT	Stroke	rs7967182	C	T	-0.040	0.003	0.804	350471	8.022E-44	174.371	0.0005
PCT	Stroke	rs8066750	C	T	0.018	0.003	0.717	350471	2.363E-12	44.607	0.0001
PCT	Stroke	rs8178824	T	C	0.108	0.007	0.030	350471	9.763E-59	237.701	0.0007
PCT	Stroke	rs877118	T	C	-0.017	0.002	0.602	350471	7.244E-13	46.786	0.0001

Table S1. SNPs information of platelet indices (PLT, PDW, MPV and PCT) with Stroke and stroke subtypes group (ALS, LAS, SVS and CES)

Exposure	Outcome	SNP	Effect allele	Other allele	Beta	SE	Eaf	N	*P* val	Fval	R2
PCT	Stroke	rs9399136	C	T	0.121	0.003	0.259	350471	1E-200	1985.545	0.0056
PCT	Stroke	rs9764455	A	G	-0.061	0.005	0.061	350471	5.992E-38	150.949	0.0004
PCT	Stroke	rs9852215	C	T	0.022	0.003	0.245	350471	3.466E-17	65.348	0.0002
PCT	AIS	rs10411361	G	A	0.017	0.002	0.484	350471	6.616E-14	50.931	0.0001
PCT	AIS	rs10779836	T	C	0.021	0.003	0.803	350471	3.856E-13	48.015	0.0001
PCT	AIS	rs10849334	G	A	-0.016	0.003	0.276	350471	2.065E-10	36.721	0.0001
PCT	AIS	rs10931934	C	T	0.018	0.002	0.608	350471	4.929E-14	51.482	0.0001
PCT	AIS	rs114694170	C	T	0.081	0.005	0.059	350471	3.921E-62	255.373	0.0007
PCT	AIS	rs11553699	G	A	0.112	0.003	0.135	350471	1E-200	1041.016	0.003
PCT	AIS	rs116234817	A	G	0.082	0.005	0.052	350471	1.297E-57	231.528	0.0007
PCT	AIS	rs11906768	C	T	-0.075	0.003	0.266	350471	8.09E-190	781.155	0.0022
PCT	AIS	rs12052715	G	C	-0.043	0.003	0.727	350471	1.297E-64	259.926	0.0007
PCT	AIS	rs12155039	A	C	-0.047	0.002	0.435	350471	6.153E-92	374.063	0.0011
PCT	AIS	rs12340895	G	C	-0.023	0.003	0.261	350471	1.267E-18	70.096	0.0002
PCT	AIS	rs12412214	A	G	-0.026	0.003	0.284	350471	1.467E-25	98.775	0.0003
PCT	AIS	rs12491937	G	A	-0.037	0.002	0.418	350471	4.086E-57	229.320	0.0007
PCT	AIS	rs12882460	T	C	0.042	0.005	0.066	350471	3.314E-20	76.980	0.0002
PCT	AIS	rs12943566	G	A	0.035	0.002	0.654	350471	6.294E-48	191.297	0.0005
PCT	AIS	rs12947592	T	C	0.039	0.003	0.176	350471	3.263E-39	154.658	0.0004
PCT	AIS	rs139974673	C	T	0.107	0.007	0.025	350471	2.455E-48	193.827	0.0006
PCT	AIS	rs144344009	C	T	-0.060	0.005	0.065	350471	1.39E-37	154.130	0.0004
PCT	AIS	rs148762611	G	A	-0.022	0.002	0.347	350471	8.102E-20	75.482	0.0002
PCT	AIS	rs149290349	A	G	-0.066	0.004	0.075	350471	1.972E-52	214.809	0.0006
PCT	AIS	rs151234	C	G	0.038	0.003	0.129	350471	3.068E-29	114.263	0.0003
PCT	AIS	rs1555405	A	G	-0.061	0.003	0.248	350471	1.14E-119	491.621	0.0014
PCT	AIS	rs16853270	A	C	0.052	0.004	0.089	350471	1.534E-38	152.407	0.0004
PCT	AIS	rs17356664	T	C	-0.030	0.002	0.307	350471	4.485E-33	130.145	0.0004
PCT	AIS	rs1736144	C	G	-0.022	0.002	0.429	350471	2.408E-21	81.059	0.0002
PCT	AIS	rs182268522	G	A	-0.069	0.009	0.017	350471	6.461E-15	56.501	0.0002
PCT	AIS	rs1984021	A	G	0.042	0.003	0.136	350471	2.759E-37	147.513	0.0004
PCT	AIS	rs210134	G	A	0.098	0.002	0.688	350471	1E-200	1466.176	0.0042
PCT	AIS	rs2155380	G	A	0.056	0.003	0.271	350471	4.9E-108	442.624	0.0013
PCT	AIS	rs2236055	G	A	-0.047	0.002	0.468	350471	4.215E-93	378.343	0.0011
PCT	AIS	rs2298993	A	G	-0.028	0.002	0.402	350471	1.522E-33	132.907	0.0004
PCT	AIS	rs2327528	A	G	0.043	0.003	0.212	350471	1.113E-52	211.357	0.0006
PCT	AIS	rs2327614	T	A	-0.015	0.002	0.543	350471	2.916E-11	40.148	0.0001
PCT	AIS	rs2411229	C	T	-0.020	0.002	0.389	350471	2.241E-17	65.271	0.0002
PCT	AIS	rs2469434	C	T	-0.023	0.002	0.406	350471	1.385E-22	86.904	0.0002
PCT	AIS	rs2699425	C	T	0.017	0.002	0.373	350471	2.03E-13	49.553	0.0001
PCT	AIS	rs272905	C	T	0.020	0.002	0.460	350471	2.387E-18	72.718	0.0002
PCT	AIS	rs2738744	G	A	0.018	0.003	0.737	350471	1.473E-12	45.465	0.0001
PCT	AIS	rs2834322	A	G	0.019	0.003	0.181	350471	5.184E-11	39.066	0.0001
PCT	AIS	rs28505677	G	C	-0.037	0.003	0.237	350471	4.99E-42	169.240	0.0005
PCT	AIS	rs2894602	G	A	-0.030	0.003	0.764	350471	5.421E-28	110.730	0.0003
PCT	AIS	rs2993488	T	C	-0.036	0.003	0.187	350471	1.746E-35	140.055	0.0004
PCT	AIS	rs351372	A	T	-0.018	0.002	0.525	350471	1.035E-15	58.327	0.0002

Table S1. SNPs information of platelet indices (PLT, PDW, MPV and PCT) with Stroke and stroke subtypes group (ALS, LAS, SVS and CES)

Exposure	Outcome	SNP	Effect allele	Other allele	Beta	SE	Eaf	N	*P* val	Fval	R2
PCT	AIS	rs35478863	G	T	0.022	0.002	0.325	350471	1.385E-18	72.921	0.0002
PCT	AIS	rs3860612	G	C	0.021	0.003	0.187	350471	9.33E-13	46.710	0.0001
PCT	AIS	rs387582	A	C	0.095	0.002	0.504	350471	1E-200	1574.563	0.0045
PCT	AIS	rs3956480	T	C	-0.016	0.002	0.465	350471	2.003E-11	44.889	0.0001
PCT	AIS	rs4020660	A	G	-0.025	0.002	0.465	350471	6.784E-27	109.354	0.0003
PCT	AIS	rs415064	C	G	-0.152	0.005	0.047	350471	6.25E-177	726.893	0.0021
PCT	AIS	rs448355	C	T	0.020	0.002	0.613	350471	2.591E-17	65.101	0.0002
PCT	AIS	rs449454	G	A	0.035	0.002	0.616	350471	9.995E-51	203.439	0.0006
PCT	AIS	rs4773860	T	C	0.038	0.002	0.522	350471	1.509E-60	246.173	0.0007
PCT	AIS	rs4800148	A	G	-0.019	0.003	0.782	350471	1.359E-12	45.296	0.0001
PCT	AIS	rs4820268	A	G	-0.025	0.002	0.537	350471	3.256E-28	109.465	0.0003
PCT	AIS	rs55966801	C	T	0.039	0.003	0.231	350471	8.494E-47	190.904	0.0005
PCT	AIS	rs56043070	A	G	-0.077	0.004	0.072	350471	4.862E-69	278.140	0.0008
PCT	AIS	rs57749886	T	C	0.025	0.002	0.649	350471	3.935E-26	101.190	0.0003
PCT	AIS	rs58434384	G	A	0.060	0.004	0.086	350471	7.501E-49	195.725	0.0006
PCT	AIS	rs59018815	A	G	-0.027	0.003	0.199	350471	5.242E-22	84.367	0.0002
PCT	AIS	rs59865663	A	G	0.036	0.003	0.203	350471	1.792E-36	146.400	0.0004
PCT	AIS	rs61750929	T	C	-0.084	0.005	0.055	350471	9.326E-64	258.375	0.0007
PCT	AIS	rs654868	G	A	-0.018	0.003	0.745	350471	3.374E-12	43.898	0.0001
PCT	AIS	rs6815294	A	G	0.026	0.002	0.573	350471	7.124E-29	113.043	0.0003
PCT	AIS	rs6993770	T	A	-0.050	0.003	0.286	350471	1.669E-88	359.560	0.001
PCT	AIS	rs703059	A	G	-0.018	0.002	0.483	350471	7.989E-15	55.827	0.0002
PCT	AIS	rs7036656	T	C	0.048	0.003	0.722	350471	3.64E-79	322.052	0.0009
PCT	AIS	rs71524003	C	G	0.026	0.003	0.290	350471	1.125E-24	96.560	0.0003
PCT	AIS	rs724322	T	C	-0.017	0.002	0.423	350471	1.116E-13	50.090	0.0001
PCT	AIS	rs73721606	T	C	-0.029	0.003	0.188	350471	7.178E-23	87.663	0.0003
PCT	AIS	rs7413585	C	T	-0.017	0.002	0.517	350471	4.924E-14	52.410	0.0001
PCT	AIS	rs75107793	A	G	0.106	0.004	0.073	350471	8.67E-130	538.573	0.0015
PCT	AIS	rs7536036	C	T	0.022	0.002	0.342	350471	3.037E-20	78.263	0.0002
PCT	AIS	rs7615916	A	G	0.020	0.003	0.248	350471	3.395E-14	52.309	0.0001
PCT	AIS	rs7636889	A	G	0.024	0.003	0.762	350471	9.324E-19	71.941	0.0002
PCT	AIS	rs76486546	T	C	0.023	0.003	0.148	350471	1.069E-12	45.802	0.0001
PCT	AIS	rs7705526	A	C	0.051	0.002	0.325	350471	1.17E-97	407.741	0.0012
PCT	AIS	rs77261872	T	C	0.047	0.003	0.126	350471	1.222E-42	170.727	0.0005
PCT	AIS	rs7762253	T	C	0.016	0.002	0.452	350471	2.35E-12	44.561	0.0001
PCT	AIS	rs7804205	A	G	-0.026	0.004	0.115	350471	4.677E-13	47.164	0.0001
PCT	AIS	rs7833924	G	A	0.029	0.002	0.430	350471	9.489E-38	149.121	0.0004
PCT	AIS	rs78565404	T	C	0.134	0.005	0.053	350471	1.8E-148	634.500	0.0018
PCT	AIS	rs7943063	A	C	0.030	0.002	0.409	350471	1.696E-38	152.715	0.0004
PCT	AIS	rs7967182	C	T	-0.040	0.003	0.804	350471	8.022E-44	174.371	0.0005
PCT	AIS	rs8066750	C	T	0.018	0.003	0.717	350471	2.363E-12	44.607	0.0001
PCT	AIS	rs8178824	T	C	0.108	0.007	0.030	350471	9.763E-59	237.701	0.0007
PCT	AIS	rs877118	T	C	-0.017	0.002	0.602	350471	7.244E-13	46.786	0.0001
PCT	AIS	rs9399136	C	T	0.121	0.003	0.259	350471	1E-200	1985.545	0.0056
PCT	AIS	rs9764455	A	G	-0.061	0.005	0.061	350471	5.992E-38	150.949	0.0004
PCT	AIS	rs9852215	C	T	0.022	0.003	0.245	350471	3.466E-17	65.348	0.0002

Table S1. SNPs information of platelet indices (PLT, PDW, MPV and PCT) with Stroke and stroke subtypes group (ALS, LAS, SVS and CES)

Exposure	Outcome	SNP	Effect allele	Other allele	Beta	SE	Eaf	N	*P* val	Fval	R2
PCT	LAS	rs10411361	G	A	0.017	0.002	0.484	350471	6.616E-14	50.931	0.0001
PCT	LAS	rs10779836	T	C	0.021	0.003	0.803	350471	3.856E-13	48.015	0.0001
PCT	LAS	rs10849334	G	A	-0.016	0.003	0.276	350471	2.065E-10	36.721	0.0001
PCT	LAS	rs10931934	C	T	0.018	0.002	0.608	350471	4.929E-14	51.482	0.0001
PCT	LAS	rs114694170	C	T	0.081	0.005	0.059	350471	3.921E-62	255.373	0.0007
PCT	LAS	rs11553699	G	A	0.112	0.003	0.135	350471	1E-200	1041.016	0.003
PCT	LAS	rs116234817	A	G	0.082	0.005	0.052	350471	1.297E-57	231.528	0.0007
PCT	LAS	rs11906768	C	T	-0.075	0.003	0.266	350471	8.09E-190	781.155	0.0022
PCT	LAS	rs12052715	G	C	-0.043	0.003	0.727	350471	1.297E-64	259.926	0.0007
PCT	LAS	rs12155039	A	C	-0.047	0.002	0.435	350471	6.153E-92	374.063	0.0011
PCT	LAS	rs12340895	G	C	-0.023	0.003	0.261	350471	1.267E-18	70.096	0.0002
PCT	LAS	rs12412214	A	G	-0.026	0.003	0.284	350471	1.467E-25	98.775	0.0003
PCT	LAS	rs12491937	G	A	-0.037	0.002	0.418	350471	4.086E-57	229.320	0.0007
PCT	LAS	rs12882460	T	C	0.042	0.005	0.066	350471	3.314E-20	76.980	0.0002
PCT	LAS	rs12943566	G	A	0.035	0.002	0.654	350471	6.294E-48	191.297	0.0005
PCT	LAS	rs12947592	T	C	0.039	0.003	0.176	350471	3.263E-39	154.658	0.0004
PCT	LAS	rs139974673	C	T	0.107	0.007	0.025	350471	2.455E-48	193.827	0.0006
PCT	LAS	rs144344009	C	T	-0.060	0.005	0.065	350471	1.39E-37	154.130	0.0004
PCT	LAS	rs148762611	G	A	-0.022	0.002	0.347	350471	8.102E-20	75.482	0.0002
PCT	LAS	rs149290349	A	G	-0.066	0.004	0.075	350471	1.972E-52	214.809	0.0006
PCT	LAS	rs151234	C	G	0.038	0.003	0.129	350471	3.068E-29	114.263	0.0003
PCT	LAS	rs1555405	A	G	-0.061	0.003	0.248	350471	1.14E-119	491.621	0.0014
PCT	LAS	rs16853270	A	C	0.052	0.004	0.089	350471	1.534E-38	152.407	0.0004
PCT	LAS	rs17356664	T	C	-0.030	0.002	0.307	350471	4.485E-33	130.145	0.0004
PCT	LAS	rs1736144	C	G	-0.022	0.002	0.429	350471	2.408E-21	81.059	0.0002
PCT	LAS	rs182268522	G	A	-0.069	0.009	0.017	350471	6.461E-15	56.501	0.0002
PCT	LAS	rs1984021	A	G	0.042	0.003	0.136	350471	2.759E-37	147.513	0.0004
PCT	LAS	rs210134	G	A	0.098	0.002	0.688	350471	1E-200	1466.176	0.0042
PCT	LAS	rs2155380	G	A	0.056	0.003	0.271	350471	4.9E-108	442.624	0.0013
PCT	LAS	rs2236055	G	A	-0.047	0.002	0.468	350471	4.215E-93	378.343	0.0011
PCT	LAS	rs2298993	A	G	-0.028	0.002	0.402	350471	1.522E-33	132.907	0.0004
PCT	LAS	rs2327528	A	G	0.043	0.003	0.212	350471	1.113E-52	211.357	0.0006
PCT	LAS	rs2327614	T	A	-0.015	0.002	0.543	350471	2.916E-11	40.148	0.0001
PCT	LAS	rs2411229	C	T	-0.020	0.002	0.389	350471	2.241E-17	65.271	0.0002
PCT	LAS	rs2469434	C	T	-0.023	0.002	0.406	350471	1.385E-22	86.904	0.0002
PCT	LAS	rs2699425	C	T	0.017	0.002	0.373	350471	2.03E-13	49.553	0.0001
PCT	LAS	rs272905	C	T	0.020	0.002	0.460	350471	2.387E-18	72.718	0.0002
PCT	LAS	rs2738744	G	A	0.018	0.003	0.737	350471	1.473E-12	45.465	0.0001
PCT	LAS	rs2834322	A	G	0.019	0.003	0.181	350471	5.184E-11	39.066	0.0001
PCT	LAS	rs28505677	G	C	-0.037	0.003	0.237	350471	4.99E-42	169.240	0.0005
PCT	LAS	rs2894602	G	A	-0.030	0.003	0.764	350471	5.421E-28	110.730	0.0003
PCT	LAS	rs2993488	T	C	-0.036	0.003	0.187	350471	1.746E-35	140.055	0.0004
PCT	LAS	rs351372	A	T	-0.018	0.002	0.525	350471	1.035E-15	58.327	0.0002
PCT	LAS	rs35478863	G	T	0.022	0.002	0.325	350471	1.385E-18	72.921	0.0002
PCT	LAS	rs3860612	G	C	0.021	0.003	0.187	350471	9.33E-13	46.710	0.0001
PCT	LAS	rs387582	A	C	0.095	0.002	0.504	350471	1E-200	1574.563	0.0045

Table S1. SNPs information of platelet indices (PLT, PDW, MPV and PCT) with Stroke and stroke subtypes group (ALS, LAS, SVS and CES)

Exposure	Outcome	SNP	Effect allele	Other allele	Beta	SE	Eaf	N	*P* val	Fval	R2
PCT	LAS	rs3956480	T	C	-0.016	0.002	0.465	350471	2.003E-11	44.889	0.0001
PCT	LAS	rs4020660	A	G	-0.025	0.002	0.465	350471	6.784E-27	109.354	0.0003
PCT	LAS	rs415064	C	G	-0.152	0.005	0.047	350471	6.25E-177	726.893	0.0021
PCT	LAS	rs448355	C	T	0.020	0.002	0.613	350471	2.591E-17	65.101	0.0002
PCT	LAS	rs449454	G	A	0.035	0.002	0.616	350471	9.995E-51	203.439	0.0006
PCT	LAS	rs4773860	T	C	0.038	0.002	0.522	350471	1.509E-60	246.173	0.0007
PCT	LAS	rs4800148	A	G	-0.019	0.003	0.782	350471	1.359E-12	45.296	0.0001
PCT	LAS	rs4820268	A	G	-0.025	0.002	0.537	350471	3.256E-28	109.465	0.0003
PCT	LAS	rs55966801	C	T	0.039	0.003	0.231	350471	8.494E-47	190.904	0.0005
PCT	LAS	rs56043070	A	G	-0.077	0.004	0.072	350471	4.862E-69	278.140	0.0008
PCT	LAS	rs57749886	T	C	0.025	0.002	0.649	350471	3.935E-26	101.190	0.0003
PCT	LAS	rs58434384	G	A	0.060	0.004	0.086	350471	7.501E-49	195.725	0.0006
PCT	LAS	rs59018815	A	G	-0.027	0.003	0.199	350471	5.242E-22	84.367	0.0002
PCT	LAS	rs59865663	A	G	0.036	0.003	0.203	350471	1.792E-36	146.400	0.0004
PCT	LAS	rs61750929	T	C	-0.084	0.005	0.055	350471	9.326E-64	258.375	0.0007
PCT	LAS	rs654868	G	A	-0.018	0.003	0.745	350471	3.374E-12	43.898	0.0001
PCT	LAS	rs6815294	A	G	0.026	0.002	0.573	350471	7.124E-29	113.043	0.0003
PCT	LAS	rs6993770	T	A	-0.050	0.003	0.286	350471	1.669E-88	359.560	0.001
PCT	LAS	rs703059	A	G	-0.018	0.002	0.483	350471	7.989E-15	55.827	0.0002
PCT	LAS	rs7036656	T	C	0.048	0.003	0.722	350471	3.64E-79	322.052	0.0009
PCT	LAS	rs71524003	C	G	0.026	0.003	0.290	350471	1.125E-24	96.560	0.0003
PCT	LAS	rs724322	T	C	-0.017	0.002	0.423	350471	1.116E-13	50.090	0.0001
PCT	LAS	rs73721606	T	C	-0.029	0.003	0.188	350471	7.178E-23	87.663	0.0003
PCT	LAS	rs7413585	C	T	-0.017	0.002	0.517	350471	4.924E-14	52.410	0.0001
PCT	LAS	rs75107793	A	G	0.106	0.004	0.073	350471	8.67E-130	538.573	0.0015
PCT	LAS	rs7536036	C	T	0.022	0.002	0.342	350471	3.037E-20	78.263	0.0002
PCT	LAS	rs7615916	A	G	0.020	0.003	0.248	350471	3.395E-14	52.309	0.0001
PCT	LAS	rs7636889	A	G	0.024	0.003	0.762	350471	9.324E-19	71.941	0.0002
PCT	LAS	rs76486546	T	C	0.023	0.003	0.148	350471	1.069E-12	45.802	0.0001
PCT	LAS	rs7705526	A	C	0.051	0.002	0.325	350471	1.17E-97	407.741	0.0012
PCT	LAS	rs77261872	T	C	0.047	0.003	0.126	350471	1.222E-42	170.727	0.0005
PCT	LAS	rs7762253	T	C	0.016	0.002	0.452	350471	2.35E-12	44.561	0.0001
PCT	LAS	rs7804205	A	G	-0.026	0.004	0.115	350471	4.677E-13	47.164	0.0001
PCT	LAS	rs7833924	G	A	0.029	0.002	0.430	350471	9.489E-38	149.121	0.0004
PCT	LAS	rs78565404	T	C	0.134	0.005	0.053	350471	1.8E-148	634.500	0.0018
PCT	LAS	rs7943063	A	C	0.030	0.002	0.409	350471	1.696E-38	152.715	0.0004
PCT	LAS	rs7967182	C	T	-0.040	0.003	0.804	350471	8.022E-44	174.371	0.0005
PCT	LAS	rs8066750	C	T	0.018	0.003	0.717	350471	2.363E-12	44.607	0.0001
PCT	LAS	rs8178824	T	C	0.108	0.007	0.030	350471	9.763E-59	237.701	0.0007
PCT	LAS	rs877118	T	C	-0.017	0.002	0.602	350471	7.244E-13	46.786	0.0001
PCT	LAS	rs9399136	C	T	0.121	0.003	0.259	350471	1E-200	1985.545	0.0056
PCT	LAS	rs9764455	A	G	-0.061	0.005	0.061	350471	5.992E-38	150.949	0.0004
PCT	LAS	rs9852215	C	T	0.022	0.003	0.245	350471	3.466E-17	65.348	0.0002
PCT	SVS	rs10411361	G	A	0.017	0.002	0.484	350471	6.616E-14	50.931	0.0001
PCT	SVS	rs10774624	A	G	-0.099	0.002	0.514	350471	1E-200	1738.360	0.0049
PCT	SVS	rs10779836	T	C	0.021	0.003	0.803	350471	3.856E-13	48.015	0.0001

Table S1. SNPs information of platelet indices (PLT, PDW, MPV and PCT) with Stroke and stroke subtypes group (ALS, LAS, SVS and CES)

Exposure	Outcome	SNP	Effect allele	Other allele	Beta	SE	Eaf	N	*P* val	Fval	R2
PCT	SVS	rs10849334	G	A	-0.016	0.003	0.276	350471	2.065E-10	36.721	0.0001
PCT	SVS	rs10931934	C	T	0.018	0.002	0.608	350471	4.929E-14	51.482	0.0001
PCT	SVS	rs114694170	C	T	0.081	0.005	0.059	350471	3.921E-62	255.373	0.0007
PCT	SVS	rs11553699	G	A	0.112	0.003	0.135	350471	1E-200	1041.016	0.003
PCT	SVS	rs116234817	A	G	0.082	0.005	0.052	350471	1.297E-57	231.528	0.0007
PCT	SVS	rs11906768	C	T	-0.075	0.003	0.266	350471	8.09E-190	781.155	0.0022
PCT	SVS	rs12052715	G	C	-0.043	0.003	0.727	350471	1.297E-64	259.926	0.0007
PCT	SVS	rs12155039	A	C	-0.047	0.002	0.435	350471	6.153E-92	374.063	0.0011
PCT	SVS	rs12340895	G	C	-0.023	0.003	0.261	350471	1.267E-18	70.096	0.0002
PCT	SVS	rs12412214	A	G	-0.026	0.003	0.284	350471	1.467E-25	98.775	0.0003
PCT	SVS	rs12491937	G	A	-0.037	0.002	0.418	350471	4.086E-57	229.320	0.0007
PCT	SVS	rs12882460	T	C	0.042	0.005	0.066	350471	3.314E-20	76.980	0.0002
PCT	SVS	rs12943566	G	A	0.035	0.002	0.654	350471	6.294E-48	191.297	0.0005
PCT	SVS	rs12947592	T	C	0.039	0.003	0.176	350471	3.263E-39	154.658	0.0004
PCT	SVS	rs139974673	C	T	0.107	0.007	0.025	350471	2.455E-48	193.827	0.0006
PCT	SVS	rs144344009	C	T	-0.060	0.005	0.065	350471	1.39E-37	154.130	0.0004
PCT	SVS	rs148762611	G	A	-0.022	0.002	0.347	350471	8.102E-20	75.482	0.0002
PCT	SVS	rs149290349	A	G	-0.066	0.004	0.075	350471	1.972E-52	214.809	0.0006
PCT	SVS	rs151234	C	G	0.038	0.003	0.129	350471	3.068E-29	114.263	0.0003
PCT	SVS	rs1555405	A	G	-0.061	0.003	0.248	350471	1.14E-119	491.621	0.0014
PCT	SVS	rs16853270	A	C	0.052	0.004	0.089	350471	1.534E-38	152.407	0.0004
PCT	SVS	rs17356664	T	C	-0.030	0.002	0.307	350471	4.485E-33	130.145	0.0004
PCT	SVS	rs1736144	C	G	-0.022	0.002	0.429	350471	2.408E-21	81.059	0.0002
PCT	SVS	rs182268522	G	A	-0.069	0.009	0.017	350471	6.461E-15	56.501	0.0002
PCT	SVS	rs1984021	A	G	0.042	0.003	0.136	350471	2.759E-37	147.513	0.0004
PCT	SVS	rs210134	G	A	0.098	0.002	0.688	350471	1E-200	1466.176	0.0042
PCT	SVS	rs2155380	G	A	0.056	0.003	0.271	350471	4.9E-108	442.624	0.0013
PCT	SVS	rs2236055	G	A	-0.047	0.002	0.468	350471	4.215E-93	378.343	0.0011
PCT	SVS	rs2298993	A	G	-0.028	0.002	0.402	350471	1.522E-33	132.907	0.0004
PCT	SVS	rs2327528	A	G	0.043	0.003	0.212	350471	1.113E-52	211.357	0.0006
PCT	SVS	rs2327614	T	A	-0.015	0.002	0.543	350471	2.916E-11	40.148	0.0001
PCT	SVS	rs2411229	C	T	-0.020	0.002	0.389	350471	2.241E-17	65.271	0.0002
PCT	SVS	rs2469434	C	T	-0.023	0.002	0.406	350471	1.385E-22	86.904	0.0002
PCT	SVS	rs2699425	C	T	0.017	0.002	0.373	350471	2.03E-13	49.553	0.0001
PCT	SVS	rs272905	C	T	0.020	0.002	0.460	350471	2.387E-18	72.718	0.0002
PCT	SVS	rs2738744	G	A	0.018	0.003	0.737	350471	1.473E-12	45.465	0.0001
PCT	SVS	rs2834322	A	G	0.019	0.003	0.181	350471	5.184E-11	39.066	0.0001
PCT	SVS	rs28505677	G	C	-0.037	0.003	0.237	350471	4.99E-42	169.240	0.0005
PCT	SVS	rs2894602	G	A	-0.030	0.003	0.764	350471	5.421E-28	110.730	0.0003
PCT	SVS	rs2993488	T	C	-0.036	0.003	0.187	350471	1.746E-35	140.055	0.0004
PCT	SVS	rs351372	A	T	-0.018	0.002	0.525	350471	1.035E-15	58.327	0.0002
PCT	SVS	rs35478863	G	T	0.022	0.002	0.325	350471	1.385E-18	72.921	0.0002
PCT	SVS	rs3860612	G	C	0.021	0.003	0.187	350471	9.33E-13	46.710	0.0001
PCT	SVS	rs387582	A	C	0.095	0.002	0.504	350471	1E-200	1574.563	0.0045
PCT	SVS	rs3956480	T	C	-0.016	0.002	0.465	350471	2.003E-11	44.889	0.0001
PCT	SVS	rs4020660	A	G	-0.025	0.002	0.465	350471	6.784E-27	109.354	0.0003

Table S1. SNPs information of platelet indices (PLT, PDW, MPV and PCT) with Stroke and stroke subtypes group (ALS, LAS, SVS and CES)

Exposure	Outcome	SNP	Effect allele	Other allele	Beta	SE	Eaf	N	*P* val	Fval	R2
PCT	SVS	rs415064	C	G	-0.152	0.005	0.047	350471	6.25E-177	726.893	0.0021
PCT	SVS	rs448355	C	T	0.020	0.002	0.613	350471	2.591E-17	65.101	0.0002
PCT	SVS	rs449454	G	A	0.035	0.002	0.616	350471	9.995E-51	203.439	0.0006
PCT	SVS	rs4773860	T	C	0.038	0.002	0.522	350471	1.509E-60	246.173	0.0007
PCT	SVS	rs4800148	A	G	-0.019	0.003	0.782	350471	1.359E-12	45.296	0.0001
PCT	SVS	rs4820268	A	G	-0.025	0.002	0.537	350471	3.256E-28	109.465	0.0003
PCT	SVS	rs55966801	C	T	0.039	0.003	0.231	350471	8.494E-47	190.904	0.0005
PCT	SVS	rs56043070	A	G	-0.077	0.004	0.072	350471	4.862E-69	278.140	0.0008
PCT	SVS	rs57749886	T	C	0.025	0.002	0.649	350471	3.935E-26	101.190	0.0003
PCT	SVS	rs58434384	G	A	0.060	0.004	0.086	350471	7.501E-49	195.725	0.0006
PCT	SVS	rs59018815	A	G	-0.027	0.003	0.199	350471	5.242E-22	84.367	0.0002
PCT	SVS	rs59865663	A	G	0.036	0.003	0.203	350471	1.792E-36	146.400	0.0004
PCT	SVS	rs61750929	T	C	-0.084	0.005	0.055	350471	9.326E-64	258.375	0.0007
PCT	SVS	rs654868	G	A	-0.018	0.003	0.745	350471	3.374E-12	43.898	0.0001
PCT	SVS	rs6815294	A	G	0.026	0.002	0.573	350471	7.124E-29	113.043	0.0003
PCT	SVS	rs6993770	T	A	-0.050	0.003	0.286	350471	1.669E-88	359.560	0.001
PCT	SVS	rs703059	A	G	-0.018	0.002	0.483	350471	7.989E-15	55.827	0.0002
PCT	SVS	rs7036656	T	C	0.048	0.003	0.722	350471	3.64E-79	322.052	0.0009
PCT	SVS	rs71524003	C	G	0.026	0.003	0.290	350471	1.125E-24	96.560	0.0003
PCT	SVS	rs724322	T	C	-0.017	0.002	0.423	350471	1.116E-13	50.090	0.0001
PCT	SVS	rs73721606	T	C	-0.029	0.003	0.188	350471	7.178E-23	87.663	0.0003
PCT	SVS	rs7413585	C	T	-0.017	0.002	0.517	350471	4.924E-14	52.410	0.0001
PCT	SVS	rs75107793	A	G	0.106	0.004	0.073	350471	8.67E-130	538.573	0.0015
PCT	SVS	rs7536036	C	T	0.022	0.002	0.342	350471	3.037E-20	78.263	0.0002
PCT	SVS	rs7615916	A	G	0.020	0.003	0.248	350471	3.395E-14	52.309	0.0001
PCT	SVS	rs7636889	A	G	0.024	0.003	0.762	350471	9.324E-19	71.941	0.0002
PCT	SVS	rs76486546	T	C	0.023	0.003	0.148	350471	1.069E-12	45.802	0.0001
PCT	SVS	rs7705526	A	C	0.051	0.002	0.325	350471	1.17E-97	407.741	0.0012
PCT	SVS	rs77261872	T	C	0.047	0.003	0.126	350471	1.222E-42	170.727	0.0005
PCT	SVS	rs7762253	T	C	0.016	0.002	0.452	350471	2.35E-12	44.561	0.0001
PCT	SVS	rs7804205	A	G	-0.026	0.004	0.115	350471	4.677E-13	47.164	0.0001
PCT	SVS	rs7833924	G	A	0.029	0.002	0.430	350471	9.489E-38	149.121	0.0004
PCT	SVS	rs78565404	T	C	0.134	0.005	0.053	350471	1.8E-148	634.500	0.0018
PCT	SVS	rs7943063	A	C	0.030	0.002	0.409	350471	1.696E-38	152.715	0.0004
PCT	SVS	rs7967182	C	T	-0.040	0.003	0.804	350471	8.022E-44	174.371	0.0005
PCT	SVS	rs8066750	C	T	0.018	0.003	0.717	350471	2.363E-12	44.607	0.0001
PCT	SVS	rs8178824	T	C	0.108	0.007	0.030	350471	9.763E-59	237.701	0.0007
PCT	SVS	rs877118	T	C	-0.017	0.002	0.602	350471	7.244E-13	46.786	0.0001
PCT	SVS	rs9399136	C	T	0.121	0.003	0.259	350471	1E-200	1985.545	0.0056
PCT	SVS	rs9764455	A	G	-0.061	0.005	0.061	350471	5.992E-38	150.949	0.0004
PCT	SVS	rs9852215	C	T	0.022	0.003	0.245	350471	3.466E-17	65.348	0.0002
PCT	CES	rs10411361	G	A	0.017	0.002	0.484	350471	6.616E-14	50.931	0.0001
PCT	CES	rs10774624	A	G	-0.099	0.002	0.514	350471	1E-200	1738.360	0.0049
PCT	CES	rs10779836	T	C	0.021	0.003	0.803	350471	3.856E-13	48.015	0.0001
PCT	CES	rs10849334	G	A	-0.016	0.003	0.276	350471	2.065E-10	36.721	0.0001
PCT	CES	rs10931934	C	T	0.018	0.002	0.608	350471	4.929E-14	51.482	0.0001

Table S1. SNPs information of platelet indices (PLT, PDW, MPV and PCT) with Stroke and stroke subtypes group (ALS, LAS, SVS and CES)

Exposure	Outcome	SNP	Effect allele	Other allele	Beta	SE	Eaf	N	*P* val	Fval	R2
PCT	CES	rs114694170	C	T	0.081	0.005	0.059	350471	3.921E-62	255.373	0.0007
PCT	CES	rs11553699	G	A	0.112	0.003	0.135	350471	1E-200	1041.016	0.003
PCT	CES	rs116234817	A	G	0.082	0.005	0.052	350471	1.297E-57	231.528	0.0007
PCT	CES	rs11906768	C	T	-0.075	0.003	0.266	350471	8.09E-190	781.155	0.0022
PCT	CES	rs12052715	G	C	-0.043	0.003	0.727	350471	1.297E-64	259.926	0.0007
PCT	CES	rs12155039	A	C	-0.047	0.002	0.435	350471	6.153E-92	374.063	0.0011
PCT	CES	rs12340895	G	C	-0.023	0.003	0.261	350471	1.267E-18	70.096	0.0002
PCT	CES	rs12412214	A	G	-0.026	0.003	0.284	350471	1.467E-25	98.775	0.0003
PCT	CES	rs12491937	G	A	-0.037	0.002	0.418	350471	4.086E-57	229.320	0.0007
PCT	CES	rs12882460	T	C	0.042	0.005	0.066	350471	3.314E-20	76.980	0.0002
PCT	CES	rs12943566	G	A	0.035	0.002	0.654	350471	6.294E-48	191.297	0.0005
PCT	CES	rs12947592	T	C	0.039	0.003	0.176	350471	3.263E-39	154.658	0.0004
PCT	CES	rs139974673	C	T	0.107	0.007	0.025	350471	2.455E-48	193.827	0.0006
PCT	CES	rs144344009	C	T	-0.060	0.005	0.065	350471	1.39E-37	154.130	0.0004
PCT	CES	rs148762611	G	A	-0.022	0.002	0.347	350471	8.102E-20	75.482	0.0002
PCT	CES	rs149290349	A	G	-0.066	0.004	0.075	350471	1.972E-52	214.809	0.0006
PCT	CES	rs151234	C	G	0.038	0.003	0.129	350471	3.068E-29	114.263	0.0003
PCT	CES	rs1555405	A	G	-0.061	0.003	0.248	350471	1.14E-119	491.621	0.0014
PCT	CES	rs16853270	A	C	0.052	0.004	0.089	350471	1.534E-38	152.407	0.0004
PCT	CES	rs17356664	T	C	-0.030	0.002	0.307	350471	4.485E-33	130.145	0.0004
PCT	CES	rs1736144	C	G	-0.022	0.002	0.429	350471	2.408E-21	81.059	0.0002
PCT	CES	rs182268522	G	A	-0.069	0.009	0.017	350471	6.461E-15	56.501	0.0002
PCT	CES	rs1984021	A	G	0.042	0.003	0.136	350471	2.759E-37	147.513	0.0004
PCT	CES	rs210134	G	A	0.098	0.002	0.688	350471	1E-200	1466.176	0.0042
PCT	CES	rs2155380	G	A	0.056	0.003	0.271	350471	4.9E-108	442.624	0.0013
PCT	CES	rs2236055	G	A	-0.047	0.002	0.468	350471	4.215E-93	378.343	0.0011
PCT	CES	rs2298993	A	G	-0.028	0.002	0.402	350471	1.522E-33	132.907	0.0004
PCT	CES	rs2327528	A	G	0.043	0.003	0.212	350471	1.113E-52	211.357	0.0006
PCT	CES	rs2327614	T	A	-0.015	0.002	0.543	350471	2.916E-11	40.148	0.0001
PCT	CES	rs2411229	C	T	-0.020	0.002	0.389	350471	2.241E-17	65.271	0.0002
PCT	CES	rs2469434	C	T	-0.023	0.002	0.406	350471	1.385E-22	86.904	0.0002
PCT	CES	rs2699425	C	T	0.017	0.002	0.373	350471	2.03E-13	49.553	0.0001
PCT	CES	rs272905	C	T	0.020	0.002	0.460	350471	2.387E-18	72.718	0.0002
PCT	CES	rs2738744	G	A	0.018	0.003	0.737	350471	1.473E-12	45.465	0.0001
PCT	CES	rs2834322	A	G	0.019	0.003	0.181	350471	5.184E-11	39.066	0.0001
PCT	CES	rs28505677	G	C	-0.037	0.003	0.237	350471	4.99E-42	169.240	0.0005
PCT	CES	rs2894602	G	A	-0.030	0.003	0.764	350471	5.421E-28	110.730	0.0003
PCT	CES	rs2993488	T	C	-0.036	0.003	0.187	350471	1.746E-35	140.055	0.0004
PCT	CES	rs351372	A	T	-0.018	0.002	0.525	350471	1.035E-15	58.327	0.0002
PCT	CES	rs35478863	G	T	0.022	0.002	0.325	350471	1.385E-18	72.921	0.0002
PCT	CES	rs3860612	G	C	0.021	0.003	0.187	350471	9.33E-13	46.710	0.0001
PCT	CES	rs387582	A	C	0.095	0.002	0.504	350471	1E-200	1574.563	0.0045
PCT	CES	rs3956480	T	C	-0.016	0.002	0.465	350471	2.003E-11	44.889	0.0001
PCT	CES	rs4020660	A	G	-0.025	0.002	0.465	350471	6.784E-27	109.354	0.0003
PCT	CES	rs415064	C	G	-0.152	0.005	0.047	350471	6.25E-177	726.893	0.0021
PCT	CES	rs448355	C	T	0.020	0.002	0.613	350471	2.591E-17	65.101	0.0002

Table S1. SNPs information of platelet indices (PLT, PDW, MPV and PCT) with Stroke and stroke subtypes group (ALS, LAS, SVS and CES)

Exposure	Outcome	SNP	Effect allele	Other allele	Beta	SE	Eaf	N	*P* val	Fval	R2
PCT	CES	rs449454	G	A	0.035	0.002	0.616	350471	9.995E-51	203.439	0.0006
PCT	CES	rs4773860	T	C	0.038	0.002	0.522	350471	1.509E-60	246.173	0.0007
PCT	CES	rs4800148	A	G	-0.019	0.003	0.782	350471	1.359E-12	45.296	0.0001
PCT	CES	rs4820268	A	G	-0.025	0.002	0.537	350471	3.256E-28	109.465	0.0003
PCT	CES	rs55966801	C	T	0.039	0.003	0.231	350471	8.494E-47	190.904	0.0005
PCT	CES	rs56043070	A	G	-0.077	0.004	0.072	350471	4.862E-69	278.140	0.0008
PCT	CES	rs57749886	T	C	0.025	0.002	0.649	350471	3.935E-26	101.190	0.0003
PCT	CES	rs58434384	G	A	0.060	0.004	0.086	350471	7.501E-49	195.725	0.0006
PCT	CES	rs59018815	A	G	-0.027	0.003	0.199	350471	5.242E-22	84.367	0.0002
PCT	CES	rs59865663	A	G	0.036	0.003	0.203	350471	1.792E-36	146.400	0.0004
PCT	CES	rs61750929	T	C	-0.084	0.005	0.055	350471	9.326E-64	258.375	0.0007
PCT	CES	rs654868	G	A	-0.018	0.003	0.745	350471	3.374E-12	43.898	0.0001
PCT	CES	rs6815294	A	G	0.026	0.002	0.573	350471	7.124E-29	113.043	0.0003
PCT	CES	rs6993770	T	A	-0.050	0.003	0.286	350471	1.669E-88	359.560	0.001
PCT	CES	rs703059	A	G	-0.018	0.002	0.483	350471	7.989E-15	55.827	0.0002
PCT	CES	rs7036656	T	C	0.048	0.003	0.722	350471	3.64E-79	322.052	0.0009
PCT	CES	rs71524003	C	G	0.026	0.003	0.290	350471	1.125E-24	96.560	0.0003
PCT	CES	rs724322	T	C	-0.017	0.002	0.423	350471	1.116E-13	50.090	0.0001
PCT	CES	rs73721606	T	C	-0.029	0.003	0.188	350471	7.178E-23	87.663	0.0003
PCT	CES	rs7413585	C	T	-0.017	0.002	0.517	350471	4.924E-14	52.410	0.0001
PCT	CES	rs75107793	A	G	0.106	0.004	0.073	350471	8.67E-130	538.573	0.0015
PCT	CES	rs7536036	C	T	0.022	0.002	0.342	350471	3.037E-20	78.263	0.0002
PCT	CES	rs7615916	A	G	0.020	0.003	0.248	350471	3.395E-14	52.309	0.0001
PCT	CES	rs7636889	A	G	0.024	0.003	0.762	350471	9.324E-19	71.941	0.0002
PCT	CES	rs76486546	T	C	0.023	0.003	0.148	350471	1.069E-12	45.802	0.0001
PCT	CES	rs7705526	A	C	0.051	0.002	0.325	350471	1.17E-97	407.741	0.0012
PCT	CES	rs77261872	T	C	0.047	0.003	0.126	350471	1.222E-42	170.727	0.0005
PCT	CES	rs7762253	T	C	0.016	0.002	0.452	350471	2.35E-12	44.561	0.0001
PCT	CES	rs7804205	A	G	-0.026	0.004	0.115	350471	4.677E-13	47.164	0.0001
PCT	CES	rs7833924	G	A	0.029	0.002	0.430	350471	9.489E-38	149.121	0.0004
PCT	CES	rs78565404	T	C	0.134	0.005	0.053	350471	1.8E-148	634.500	0.0018
PCT	CES	rs7943063	A	C	0.030	0.002	0.409	350471	1.696E-38	152.715	0.0004
PCT	CES	rs7967182	C	T	-0.040	0.003	0.804	350471	8.022E-44	174.371	0.0005
PCT	CES	rs8066750	C	T	0.018	0.003	0.717	350471	2.363E-12	44.607	0.0001
PCT	CES	rs8178824	T	C	0.108	0.007	0.030	350471	9.763E-59	237.701	0.0007
PCT	CES	rs877118	T	C	-0.017	0.002	0.602	350471	7.244E-13	46.786	0.0001
PCT	CES	rs9399136	C	T	0.121	0.003	0.259	350471	1E-200	1985.545	0.0056
PCT	CES	rs9764455	A	G	-0.061	0.005	0.061	350471	5.992E-38	150.949	0.0004
PCT	CES	rs9852215	C	T	0.022	0.003	0.245	350471	3.466E-17	65.348	0.0002
MPV	Stroke	rs10140498	G	A	0.027	0.003	0.169	350470	1.199E-17	73.007	0.0002
MPV	Stroke	rs10186787	C	T	0.025	0.003	0.692	350470	9.402E-22	92.505	0.0003
MPV	Stroke	rs10509742	T	A	-0.066	0.005	0.063	350470	7.943E-35	179.183	0.0005
MPV	Stroke	rs10813766	G	T	-0.057	0.003	0.662	350470	8.75E-115	517.135	0.0015
MPV	Stroke	rs10954750	G	C	0.029	0.003	0.789	350470	1.138E-22	98.605	0.0003
MPV	Stroke	rs10995445	T	A	-0.093	0.002	0.472	350470	1E-200	1502.703	0.0043
MPV	Stroke	rs11064074	T	C	0.101	0.002	0.462	350470	1E-200	1782.627	0.0051

Table S1. SNPs information of platelet indices (PLT, PDW, MPV and PCT) with Stroke and stroke subtypes group (ALS, LAS, SVS and CES)

Exposure	Outcome	SNP	Effect allele	Other allele	Beta	SE	Eaf	N	*P* val	Fval	R2
MPV	Stroke	rs11071720	C	T	-0.057	0.003	0.702	350470	5.15E-104	469.294	0.0013
MPV	Stroke	rs11121529	G	C	0.082	0.004	0.114	350470	3.76E-105	473.407	0.0013
MPV	Stroke	rs11170863	T	C	-0.055	0.004	0.131	350470	7.856E-55	245.052	0.0007
MPV	Stroke	rs111780165	A	G	-0.044	0.005	0.070	350470	6.473E-21	87.956	0.0003
MPV	Stroke	rs114685606	T	A	0.234	0.006	0.046	350470	1E-200	1680.462	0.0048
MPV	Stroke	rs114968084	A	G	0.100	0.007	0.030	350470	3.327E-43	202.336	0.0006
MPV	Stroke	rs11620922	C	G	-0.042	0.003	0.233	350470	6.808E-50	220.605	0.0006
MPV	Stroke	rs11734099	A	G	-0.104	0.003	0.176	350470	1E-200	1111.401	0.0032
MPV	Stroke	rs11789898	T	G	-0.053	0.003	0.166	350470	1.654E-61	274.386	0.0008
MPV	Stroke	rs11808063	G	A	-0.027	0.003	0.306	350470	1.589E-25	110.964	0.0003
MPV	Stroke	rs12232825	G	A	0.103	0.009	0.017	350470	5.999E-29	127.278	0.0004
MPV	Stroke	rs12616493	T	C	0.019	0.002	0.556	350470	1.004E-14	60.165	0.0002
MPV	Stroke	rs12979891	T	C	-0.029	0.002	0.539	350470	5.721E-34	148.097	0.0004
MPV	Stroke	rs138092465	C	G	-0.117	0.003	0.253	350470	1E-200	1831.995	0.0052
MPV	Stroke	rs145243487	T	A	-0.112	0.005	0.059	350470	4.38E-106	491.192	0.0014
MPV	Stroke	rs146337807	T	C	0.314	0.005	0.054	350470	1E-200	3581.205	0.0101
MPV	Stroke	rs151234	C	G	-0.066	0.004	0.129	350470	9.945E-76	339.683	0.001
MPV	Stroke	rs17116384	G	A	0.026	0.003	0.320	350470	1.857E-24	104.935	0.0003
MPV	Stroke	rs1736013	C	G	-0.018	0.002	0.432	350470	2.093E-14	58.330	0.0002
MPV	Stroke	rs17411126	C	T	-0.020	0.003	0.264	350470	1.474E-13	54.490	0.0002
MPV	Stroke	rs1883258	T	G	0.043	0.002	0.384	350470	1.808E-68	306.742	0.0009
MPV	Stroke	rs1894572	C	T	0.026	0.002	0.473	350470	6.39E-27	115.140	0.0003
MPV	Stroke	rs2038479	A	C	0.133	0.003	0.804	350470	1E-200	1972.345	0.0056
MPV	Stroke	rs2079356	T	C	-0.109	0.003	0.398	350470	1E-200	1995.101	0.0057
MPV	Stroke	rs2137082	C	G	0.036	0.002	0.519	350470	4.439E-52	230.446	0.0007
MPV	Stroke	rs216812	A	G	0.019	0.003	0.323	350470	5.77E-13	53.338	0.0002
MPV	Stroke	rs2297066	G	C	-0.078	0.003	0.242	350470	7.14E-171	775.716	0.0022
MPV	Stroke	rs2313211	A	T	-0.023	0.002	0.554	350470	6.926E-21	88.422	0.0003
MPV	Stroke	rs236691	C	G	-0.078	0.003	0.184	350470	9.4E-140	637.738	0.0018
MPV	Stroke	rs2413712	G	A	-0.031	0.003	0.666	350470	3.141E-35	153.908	0.0004
MPV	Stroke	rs2516471	A	G	-0.125	0.005	0.063	350470	6.46E-141	649.149	0.0018
MPV	Stroke	rs2700982	G	C	0.018	0.002	0.458	350470	2.496E-13	53.897	0.0002
MPV	Stroke	rs2709864	A	G	0.048	0.004	0.886	350470	3.977E-38	166.495	0.0005
MPV	Stroke	rs2783179	C	T	0.023	0.003	0.368	350470	1.771E-19	83.034	0.0002
MPV	Stroke	rs28396651	G	A	0.033	0.002	0.601	350470	3.927E-42	184.813	0.0005
MPV	Stroke	rs28633508	T	C	0.028	0.003	0.313	350470	2.53E-26	114.426	0.0003
MPV	Stroke	rs3135064	C	T	-0.020	0.002	0.547	350470	1.601E-17	72.904	0.0002
MPV	Stroke	rs34379299	G	A	-0.027	0.003	0.195	350470	4.097E-19	80.094	0.0002
MPV	Stroke	rs346738	C	T	-0.056	0.003	0.655	350470	2.33E-108	497.273	0.0014
MPV	Stroke	rs34991606	T	C	-0.052	0.003	0.140	350470	2.161E-51	227.537	0.0006
MPV	Stroke	rs35554791	G	A	-0.064	0.005	0.062	350470	1.198E-37	166.881	0.0005
MPV	Stroke	rs35740053	A	C	0.019	0.003	0.206	350470	7.903E-11	42.203	0.0001
MPV	Stroke	rs367990687	T	A	-0.142	0.002	0.489	350470	1E-200	3574.165	0.0101
MPV	Stroke	rs3740995	C	T	-0.035	0.003	0.846	350470	1.761E-26	114.168	0.0003
MPV	Stroke	rs3752112	G	T	-0.026	0.003	0.186	350470	6.422E-17	69.991	0.0002
MPV	Stroke	rs3770781	G	A	-0.035	0.002	0.441	350470	8.985E-49	217.109	0.0006

Table S1. SNPs information of platelet indices (PLT, PDW, MPV and PCT) with Stroke and stroke subtypes group (ALS, LAS, SVS and CES)

Exposure	Outcome	SNP	Effect allele	Other allele	Beta	SE	Eaf	N	*P* val	Fval	R2
MPV	Stroke	rs3826508	C	G	-0.053	0.005	0.070	350470	6.759E-30	129.247	0.0004
MPV	Stroke	rs3937015	C	T	-0.134	0.009	0.981	350470	6.081E-53	236.360	0.0007
MPV	Stroke	rs4814779	A	C	-0.077	0.003	0.266	350470	1.61E-177	806.807	0.0023
MPV	Stroke	rs4941565	G	T	-0.032	0.003	0.652	350470	3.056E-37	162.127	0.0005
MPV	Stroke	rs55809585	G	A	-0.052	0.003	0.816	350470	2.296E-64	287.360	0.0008
MPV	Stroke	rs56316167	T	C	-0.017	0.003	0.419	350470	1.834E-11	48.236	0.0001
MPV	Stroke	rs57843631	T	C	0.265	0.009	0.019	350470	8.45E-190	926.422	0.0026
MPV	Stroke	rs60757417	G	C	0.115	0.005	0.060	350470	4.3E-115	530.535	0.0015
MPV	Stroke	rs6105433	G	T	-0.042	0.004	0.136	350470	1.502E-33	146.953	0.0004
MPV	Stroke	rs61776822	A	G	0.032	0.002	0.500	350470	1.137E-39	179.244	0.0005
MPV	Stroke	rs61896141	C	A	-0.055	0.003	0.190	350470	1.212E-72	325.437	0.0009
MPV	Stroke	rs61919382	T	C	-0.041	0.005	0.073	350470	4.457E-19	79.675	0.0002
MPV	Stroke	rs62047991	G	A	-0.021	0.003	0.303	350470	2.964E-15	62.354	0.0002
MPV	Stroke	rs62116961	C	G	-0.016	0.002	0.377	350470	1.373E-10	41.302	0.0001
MPV	Stroke	rs62338220	A	G	-0.027	0.004	0.083	350470	3.918E-10	39.755	0.0001
MPV	Stroke	rs655029	A	G	-0.087	0.003	0.708	350470	1E-200	1099.765	0.0031
MPV	Stroke	rs671339	G	A	-0.050	0.003	0.737	350470	7.106E-77	344.568	0.001
MPV	Stroke	rs6802617	T	C	-0.116	0.003	0.747	350470	1E-200	1797.537	0.0051
MPV	Stroke	rs6848819	C	A	-0.070	0.007	0.033	350470	1.177E-25	109.099	0.0003
MPV	Stroke	rs691299	T	G	-0.026	0.003	0.692	350470	6.699E-24	101.535	0.0003
MPV	Stroke	rs6993770	T	A	0.046	0.003	0.286	350470	3.243E-67	299.457	0.0009
MPV	Stroke	rs7008182	A	T	0.041	0.002	0.490	350470	4.58E-65	290.704	0.0008
MPV	Stroke	rs7116797	G	A	-0.031	0.004	0.893	350470	3.176E-15	62.577	0.0002
MPV	Stroke	rs72847212	C	T	0.027	0.003	0.202	350470	8.185E-20	83.309	0.0002
MPV	Stroke	rs7285107	A	T	-0.024	0.003	0.261	350470	8.076E-18	75.525	0.0002
MPV	Stroke	rs73229934	T	C	0.384	0.008	0.024	350470	1E-200	2463.849	0.007
MPV	Stroke	rs73723358	G	A	-0.071	0.004	0.122	350470	7.386E-82	383.992	0.0011
MPV	Stroke	rs7427439	G	A	-0.029	0.003	0.748	350470	6.005E-26	111.330	0.0003
MPV	Stroke	rs74324636	G	T	-0.082	0.004	0.084	350470	8.584E-81	361.852	0.001
MPV	Stroke	rs74505413	G	T	-0.098	0.006	0.043	350470	5.895E-61	273.358	0.0008
MPV	Stroke	rs7517721	C	T	-0.040	0.003	0.643	350470	3.172E-56	252.780	0.0007
MPV	Stroke	rs7590948	A	G	-0.016	0.002	0.550	350470	3.627E-11	43.952	0.0001
MPV	Stroke	rs7700960	C	T	0.051	0.008	0.024	350470	1.272E-10	41.966	0.0001
MPV	Stroke	rs78909033	A	G	-0.177	0.003	0.135	350470	1E-200	2594.331	0.0073
MPV	Stroke	rs7894089	C	T	0.024	0.003	0.161	350470	1.993E-13	54.113	0.0002
MPV	Stroke	rs79548680	C	G	0.044	0.004	0.135	350470	5.216E-36	158.936	0.0005
MPV	Stroke	rs80133860	T	G	-0.046	0.007	0.032	350470	3.161E-11	45.656	0.0001
MPV	Stroke	rs80250978	T	C	0.037	0.003	0.151	350470	8.498E-28	120.041	0.0003
MPV	Stroke	rs8099412	C	T	-0.084	0.002	0.475	350470	1E-200	1247.612	0.0035
MPV	Stroke	rs912324	T	A	0.018	0.003	0.743	350470	1.256E-10	41.715	0.0001
MPV	Stroke	rs9326600	A	G	-0.105	0.002	0.428	350470	1E-200	1919.135	0.0054
MPV	Stroke	rs9368600	G	C	0.021	0.003	0.287	350470	5.083E-15	61.189	0.0002
MPV	Stroke	rs9407530	A	G	-0.038	0.003	0.289	350470	9.303E-41	204.890	0.0006
MPV	Stroke	rs9549753	A	C	0.048	0.002	0.532	350470	6.145E-88	396.258	0.0011
MPV	Stroke	rs972577	T	C	0.061	0.002	0.552	350470	1.17E-144	655.297	0.0019
MPV	Stroke	rs976552	C	A	-0.020	0.003	0.240	350470	3.198E-12	48.888	0.0001

Table S1. SNPs information of platelet indices (PLT, PDW, MPV and PCT) with Stroke and stroke subtypes group (ALS, LAS, SVS and CES)

Exposure	Outcome	SNP	Effect allele	Other allele	Beta	SE	Eaf	N	*P* val	Fval	R2
MPV	Stroke	rs9823526	G	C	0.101	0.002	0.541	350470	1E-200	1797.817	0.0051
MPV	Stroke	rs9925477	G	T	0.033	0.002	0.468	350470	1.809E-42	192.370	0.0005
MPV	Stroke	rs9962297	C	A	0.016	0.002	0.614	350470	5.813E-11	42.952	0.0001
MPV	AIS	rs10140498	G	A	0.027	0.003	0.169	350470	1.199E-17	73.007	0.0002
MPV	AIS	rs10186787	C	T	0.025	0.003	0.692	350470	9.402E-22	92.505	0.0003
MPV	AIS	rs10509742	T	A	-0.066	0.005	0.063	350470	7.943E-35	179.183	0.0005
MPV	AIS	rs10813766	G	T	-0.057	0.003	0.662	350470	8.75E-115	517.135	0.0015
MPV	AIS	rs10954750	G	C	0.029	0.003	0.789	350470	1.138E-22	98.605	0.0003
MPV	AIS	rs10995445	T	A	-0.093	0.002	0.472	350470	1E-200	1502.703	0.0043
MPV	AIS	rs11064074	T	C	0.101	0.002	0.462	350470	1E-200	1782.627	0.0051
MPV	AIS	rs11071720	C	T	-0.057	0.003	0.702	350470	5.15E-104	469.294	0.0013
MPV	AIS	rs11121529	G	C	0.082	0.004	0.114	350470	3.76E-105	473.407	0.0013
MPV	AIS	rs11170863	T	C	-0.055	0.004	0.131	350470	7.856E-55	245.052	0.0007
MPV	AIS	rs111780165	A	G	-0.044	0.005	0.070	350470	6.473E-21	87.956	0.0003
MPV	AIS	rs114685606	T	A	0.234	0.006	0.046	350470	1E-200	1680.462	0.0048
MPV	AIS	rs114968084	A	G	0.100	0.007	0.030	350470	3.327E-43	202.336	0.0006
MPV	AIS	rs11620922	C	G	-0.042	0.003	0.233	350470	6.808E-50	220.605	0.0006
MPV	AIS	rs11734099	A	G	-0.104	0.003	0.176	350470	1E-200	1111.401	0.0032
MPV	AIS	rs11789898	T	G	-0.053	0.003	0.166	350470	1.654E-61	274.386	0.0008
MPV	AIS	rs11808063	G	A	-0.027	0.003	0.306	350470	1.589E-25	110.964	0.0003
MPV	AIS	rs12232825	G	A	0.103	0.009	0.017	350470	5.999E-29	127.278	0.0004
MPV	AIS	rs12616493	T	C	0.019	0.002	0.556	350470	1.004E-14	60.165	0.0002
MPV	AIS	rs12979891	T	C	-0.029	0.002	0.539	350470	5.721E-34	148.097	0.0004
MPV	AIS	rs138092465	C	G	-0.117	0.003	0.253	350470	1E-200	1831.995	0.0052
MPV	AIS	rs145243487	T	A	-0.112	0.005	0.059	350470	4.38E-106	491.192	0.0014
MPV	AIS	rs146337807	T	C	0.314	0.005	0.054	350470	1E-200	3581.205	0.0101
MPV	AIS	rs151234	C	G	-0.066	0.004	0.129	350470	9.945E-76	339.683	0.001
MPV	AIS	rs17116384	G	A	0.026	0.003	0.320	350470	1.857E-24	104.935	0.0003
MPV	AIS	rs1736013	C	G	-0.018	0.002	0.432	350470	2.093E-14	58.330	0.0002
MPV	AIS	rs17411126	C	T	-0.020	0.003	0.264	350470	1.474E-13	54.490	0.0002
MPV	AIS	rs1883258	T	G	0.043	0.002	0.384	350470	1.808E-68	306.742	0.0009
MPV	AIS	rs1894572	C	T	0.026	0.002	0.473	350470	6.39E-27	115.140	0.0003
MPV	AIS	rs2038479	A	C	0.133	0.003	0.804	350470	1E-200	1972.345	0.0056
MPV	AIS	rs2079356	T	C	-0.109	0.003	0.398	350470	1E-200	1995.101	0.0057
MPV	AIS	rs2137082	C	G	0.036	0.002	0.519	350470	4.439E-52	230.446	0.0007
MPV	AIS	rs216812	A	G	0.019	0.003	0.323	350470	5.77E-13	53.338	0.0002
MPV	AIS	rs2297066	G	C	-0.078	0.003	0.242	350470	7.14E-171	775.716	0.0022
MPV	AIS	rs2313211	A	T	-0.023	0.002	0.554	350470	6.926E-21	88.422	0.0003
MPV	AIS	rs236691	C	G	-0.078	0.003	0.184	350470	9.4E-140	637.738	0.0018
MPV	AIS	rs2413712	G	A	-0.031	0.003	0.666	350470	3.141E-35	153.908	0.0004
MPV	AIS	rs2516471	A	G	-0.125	0.005	0.063	350470	6.46E-141	649.149	0.0018
MPV	AIS	rs2700982	G	C	0.018	0.002	0.458	350470	2.496E-13	53.897	0.0002
MPV	AIS	rs2709864	A	G	0.048	0.004	0.886	350470	3.977E-38	166.495	0.0005
MPV	AIS	rs2783179	C	T	0.023	0.003	0.368	350470	1.771E-19	83.034	0.0002
MPV	AIS	rs28396651	G	A	0.033	0.002	0.601	350470	3.927E-42	184.813	0.0005
MPV	AIS	rs28633508	T	C	0.028	0.003	0.313	350470	2.53E-26	114.426	0.0003

Table S1. SNPs information of platelet indices (PLT, PDW, MPV and PCT) with Stroke and stroke subtypes group (ALS, LAS, SVS and CES)

Exposure	Outcome	SNP	Effect allele	Other allele	Beta	SE	Eaf	N	*P* val	Fval	R2
MPV	AIS	rs3135064	C	T	-0.020	0.002	0.547	350470	1.601E-17	72.904	0.0002
MPV	AIS	rs34379299	G	A	-0.027	0.003	0.195	350470	4.097E-19	80.094	0.0002
MPV	AIS	rs346738	C	T	-0.056	0.003	0.655	350470	2.33E-108	497.273	0.0014
MPV	AIS	rs34991606	T	C	-0.052	0.003	0.140	350470	2.161E-51	227.537	0.0006
MPV	AIS	rs35554791	G	A	-0.064	0.005	0.062	350470	1.198E-37	166.881	0.0005
MPV	AIS	rs35740053	A	C	0.019	0.003	0.206	350470	7.903E-11	42.203	0.0001
MPV	AIS	rs367990687	T	A	-0.142	0.002	0.489	350470	1E-200	3574.165	0.0101
MPV	AIS	rs3740995	C	T	-0.035	0.003	0.846	350470	1.761E-26	114.168	0.0003
MPV	AIS	rs3752112	G	T	-0.026	0.003	0.186	350470	6.422E-17	69.991	0.0002
MPV	AIS	rs3770781	G	A	-0.035	0.002	0.441	350470	8.985E-49	217.109	0.0006
MPV	AIS	rs3826508	C	G	-0.053	0.005	0.070	350470	6.759E-30	129.247	0.0004
MPV	AIS	rs3937015	C	T	-0.134	0.009	0.981	350470	6.081E-53	236.360	0.0007
MPV	AIS	rs4814779	A	C	-0.077	0.003	0.266	350470	1.61E-177	806.807	0.0023
MPV	AIS	rs4941565	G	T	-0.032	0.003	0.652	350470	3.056E-37	162.127	0.0005
MPV	AIS	rs55809585	G	A	-0.052	0.003	0.816	350470	2.296E-64	287.360	0.0008
MPV	AIS	rs56316167	T	C	-0.017	0.003	0.419	350470	1.834E-11	48.236	0.0001
MPV	AIS	rs57843631	T	C	0.265	0.009	0.019	350470	8.45E-190	926.422	0.0026
MPV	AIS	rs60757417	G	C	0.115	0.005	0.060	350470	4.3E-115	530.535	0.0015
MPV	AIS	rs6105433	G	T	-0.042	0.004	0.136	350470	1.502E-33	146.953	0.0004
MPV	AIS	rs61776822	A	G	0.032	0.002	0.500	350470	1.137E-39	179.244	0.0005
MPV	AIS	rs61896141	C	A	-0.055	0.003	0.190	350470	1.212E-72	325.437	0.0009
MPV	AIS	rs61919382	T	C	-0.041	0.005	0.073	350470	4.457E-19	79.675	0.0002
MPV	AIS	rs62047991	G	A	-0.021	0.003	0.303	350470	2.964E-15	62.354	0.0002
MPV	AIS	rs62116961	C	G	-0.016	0.002	0.377	350470	1.373E-10	41.302	0.0001
MPV	AIS	rs62338220	A	G	-0.027	0.004	0.083	350470	3.918E-10	39.755	0.0001
MPV	AIS	rs655029	A	G	-0.087	0.003	0.708	350470	1E-200	1099.765	0.0031
MPV	AIS	rs671339	G	A	-0.050	0.003	0.737	350470	7.106E-77	344.568	0.001
MPV	AIS	rs6802617	T	C	-0.116	0.003	0.747	350470	1E-200	1797.537	0.0051
MPV	AIS	rs6848819	C	A	-0.070	0.007	0.033	350470	1.177E-25	109.099	0.0003
MPV	AIS	rs691299	T	G	-0.026	0.003	0.692	350470	6.699E-24	101.535	0.0003
MPV	AIS	rs6993770	T	A	0.046	0.003	0.286	350470	3.243E-67	299.457	0.0009
MPV	AIS	rs7008182	A	T	0.041	0.002	0.490	350470	4.58E-65	290.704	0.0008
MPV	AIS	rs7116797	G	A	-0.031	0.004	0.893	350470	3.176E-15	62.577	0.0002
MPV	AIS	rs72847212	C	T	0.027	0.003	0.202	350470	8.185E-20	83.309	0.0002
MPV	AIS	rs7285107	A	T	-0.024	0.003	0.261	350470	8.076E-18	75.525	0.0002
MPV	AIS	rs73229934	T	C	0.384	0.008	0.024	350470	1E-200	2463.849	0.007
MPV	AIS	rs73723358	G	A	-0.071	0.004	0.122	350470	7.386E-82	383.992	0.0011
MPV	AIS	rs7427439	G	A	-0.029	0.003	0.748	350470	6.005E-26	111.330	0.0003
MPV	AIS	rs74324636	G	T	-0.082	0.004	0.084	350470	8.584E-81	361.852	0.001
MPV	AIS	rs74505413	G	T	-0.098	0.006	0.043	350470	5.895E-61	273.358	0.0008
MPV	AIS	rs7517721	C	T	-0.040	0.003	0.643	350470	3.172E-56	252.780	0.0007
MPV	AIS	rs7590948	A	G	-0.016	0.002	0.550	350470	3.627E-11	43.952	0.0001
MPV	AIS	rs7700960	C	T	0.051	0.008	0.024	350470	1.272E-10	41.966	0.0001
MPV	AIS	rs78909033	A	G	-0.177	0.003	0.135	350470	1E-200	2594.331	0.0073
MPV	AIS	rs7894089	C	T	0.024	0.003	0.161	350470	1.993E-13	54.113	0.0002
MPV	AIS	rs79548680	C	G	0.044	0.004	0.135	350470	5.216E-36	158.936	0.0005

Table S1. SNPs information of platelet indices (PLT, PDW, MPV and PCT) with Stroke and stroke subtypes group (ALS, LAS, SVS and CES)

Exposure	Outcome	SNP	Effect allele	Other allele	Beta	SE	Eaf	N	*P* val	Fval	R2
MPV	AIS	rs80133860	T	G	-0.046	0.007	0.032	350470	3.161E-11	45.656	0.0001
MPV	AIS	rs80250978	T	C	0.037	0.003	0.151	350470	8.498E-28	120.041	0.0003
MPV	AIS	rs8099412	C	T	-0.084	0.002	0.475	350470	1E-200	1247.612	0.0035
MPV	AIS	rs912324	T	A	0.018	0.003	0.743	350470	1.256E-10	41.715	0.0001
MPV	AIS	rs9326600	A	G	-0.105	0.002	0.428	350470	1E-200	1919.135	0.0054
MPV	AIS	rs9368600	G	C	0.021	0.003	0.287	350470	5.083E-15	61.189	0.0002
MPV	AIS	rs9407530	A	G	-0.038	0.003	0.289	350470	9.303E-41	204.890	0.0006
MPV	AIS	rs9549753	A	C	0.048	0.002	0.532	350470	6.145E-88	396.258	0.0011
MPV	AIS	rs972577	T	C	0.061	0.002	0.552	350470	1.17E-144	655.297	0.0019
MPV	AIS	rs976552	C	A	-0.020	0.003	0.240	350470	3.198E-12	48.888	0.0001
MPV	AIS	rs9823526	G	C	0.101	0.002	0.541	350470	1E-200	1797.817	0.0051
MPV	AIS	rs9925477	G	T	0.033	0.002	0.468	350470	1.809E-42	192.370	0.0005
MPV	AIS	rs9962297	C	A	0.016	0.002	0.614	350470	5.813E-11	42.952	0.0001
MPV	LAS	rs10140498	G	A	0.027	0.003	0.169	350470	1.199E-17	73.007	0.0002
MPV	LAS	rs10186787	C	T	0.025	0.003	0.692	350470	9.402E-22	92.505	0.0003
MPV	LAS	rs10509742	T	A	-0.066	0.005	0.063	350470	7.943E-35	179.183	0.0005
MPV	LAS	rs10813766	G	T	-0.057	0.003	0.662	350470	8.75E-115	517.135	0.0015
MPV	LAS	rs10954750	G	C	0.029	0.003	0.789	350470	1.138E-22	98.605	0.0003
MPV	LAS	rs10995445	T	A	-0.093	0.002	0.472	350470	1E-200	1502.703	0.0043
MPV	LAS	rs11064074	T	C	0.101	0.002	0.462	350470	1E-200	1782.627	0.0051
MPV	LAS	rs11071720	C	T	-0.057	0.003	0.702	350470	5.15E-104	469.294	0.0013
MPV	LAS	rs11121529	G	C	0.082	0.004	0.114	350470	3.76E-105	473.407	0.0013
MPV	LAS	rs11170863	T	C	-0.055	0.004	0.131	350470	7.856E-55	245.052	0.0007
MPV	LAS	rs111780165	A	G	-0.044	0.005	0.070	350470	6.473E-21	87.956	0.0003
MPV	LAS	rs114685606	T	A	0.234	0.006	0.046	350470	1E-200	1680.462	0.0048
MPV	LAS	rs114968084	A	G	0.100	0.007	0.030	350470	3.327E-43	202.336	0.0006
MPV	LAS	rs11620922	C	G	-0.042	0.003	0.233	350470	6.808E-50	220.605	0.0006
MPV	LAS	rs11734099	A	G	-0.104	0.003	0.176	350470	1E-200	1111.401	0.0032
MPV	LAS	rs11789898	T	G	-0.053	0.003	0.166	350470	1.654E-61	274.386	0.0008
MPV	LAS	rs11808063	G	A	-0.027	0.003	0.306	350470	1.589E-25	110.964	0.0003
MPV	LAS	rs12232825	G	A	0.103	0.009	0.017	350470	5.999E-29	127.278	0.0004
MPV	LAS	rs12616493	T	C	0.019	0.002	0.556	350470	1.004E-14	60.165	0.0002
MPV	LAS	rs12979891	T	C	-0.029	0.002	0.539	350470	5.721E-34	148.097	0.0004
MPV	LAS	rs138092465	C	G	-0.117	0.003	0.253	350470	1E-200	1831.995	0.0052
MPV	LAS	rs145243487	T	A	-0.112	0.005	0.059	350470	4.38E-106	491.192	0.0014
MPV	LAS	rs146337807	T	C	0.314	0.005	0.054	350470	1E-200	3581.205	0.0101
MPV	LAS	rs151234	C	G	-0.066	0.004	0.129	350470	9.945E-76	339.683	0.001
MPV	LAS	rs17116384	G	A	0.026	0.003	0.320	350470	1.857E-24	104.935	0.0003
MPV	LAS	rs1736013	C	G	-0.018	0.002	0.432	350470	2.093E-14	58.330	0.0002
MPV	LAS	rs17411126	C	T	-0.020	0.003	0.264	350470	1.474E-13	54.490	0.0002
MPV	LAS	rs1883258	T	G	0.043	0.002	0.384	350470	1.808E-68	306.742	0.0009
MPV	LAS	rs1894572	C	T	0.026	0.002	0.473	350470	6.39E-27	115.140	0.0003
MPV	LAS	rs2038479	A	C	0.133	0.003	0.804	350470	1E-200	1972.345	0.0056
MPV	LAS	rs2079356	T	C	-0.109	0.003	0.398	350470	1E-200	1995.101	0.0057
MPV	LAS	rs2137082	C	G	0.036	0.002	0.519	350470	4.439E-52	230.446	0.0007
MPV	LAS	rs216812	A	G	0.019	0.003	0.323	350470	5.77E-13	53.338	0.0002

Table S1. SNPs information of platelet indices (PLT, PDW, MPV and PCT) with Stroke and stroke subtypes group (ALS, LAS, SVS and CES)

Exposure	Outcome	SNP	Effect allele	Other allele	Beta	SE	Eaf	N	*P* val	Fval	R2
MPV	LAS	rs2297066	G	C	-0.078	0.003	0.242	350470	7.14E-171	775.716	0.0022
MPV	LAS	rs2313211	A	T	-0.023	0.002	0.554	350470	6.926E-21	88.422	0.0003
MPV	LAS	rs236691	C	G	-0.078	0.003	0.184	350470	9.4E-140	637.738	0.0018
MPV	LAS	rs2413712	G	A	-0.031	0.003	0.666	350470	3.141E-35	153.908	0.0004
MPV	LAS	rs2516471	A	G	-0.125	0.005	0.063	350470	6.46E-141	649.149	0.0018
MPV	LAS	rs2700982	G	C	0.018	0.002	0.458	350470	2.496E-13	53.897	0.0002
MPV	LAS	rs2709864	A	G	0.048	0.004	0.886	350470	3.977E-38	166.495	0.0005
MPV	LAS	rs2783179	C	T	0.023	0.003	0.368	350470	1.771E-19	83.034	0.0002
MPV	LAS	rs28396651	G	A	0.033	0.002	0.601	350470	3.927E-42	184.813	0.0005
MPV	LAS	rs28633508	T	C	0.028	0.003	0.313	350470	2.53E-26	114.426	0.0003
MPV	LAS	rs3135064	C	T	-0.020	0.002	0.547	350470	1.601E-17	72.904	0.0002
MPV	LAS	rs34379299	G	A	-0.027	0.003	0.195	350470	4.097E-19	80.094	0.0002
MPV	LAS	rs346738	C	T	-0.056	0.003	0.655	350470	2.33E-108	497.273	0.0014
MPV	LAS	rs34991606	T	C	-0.052	0.003	0.140	350470	2.161E-51	227.537	0.0006
MPV	LAS	rs35554791	G	A	-0.064	0.005	0.062	350470	1.198E-37	166.881	0.0005
MPV	LAS	rs35740053	A	C	0.019	0.003	0.206	350470	7.903E-11	42.203	0.0001
MPV	LAS	rs367990687	T	A	-0.142	0.002	0.489	350470	1E-200	3574.165	0.0101
MPV	LAS	rs3740995	C	T	-0.035	0.003	0.846	350470	1.761E-26	114.168	0.0003
MPV	LAS	rs3752112	G	T	-0.026	0.003	0.186	350470	6.422E-17	69.991	0.0002
MPV	LAS	rs3770781	G	A	-0.035	0.002	0.441	350470	8.985E-49	217.109	0.0006
MPV	LAS	rs3826508	C	G	-0.053	0.005	0.070	350470	6.759E-30	129.247	0.0004
MPV	LAS	rs3937015	C	T	-0.134	0.009	0.981	350470	6.081E-53	236.360	0.0007
MPV	LAS	rs4814779	A	C	-0.077	0.003	0.266	350470	1.61E-177	806.807	0.0023
MPV	LAS	rs4941565	G	T	-0.032	0.003	0.652	350470	3.056E-37	162.127	0.0005
MPV	LAS	rs55809585	G	A	-0.052	0.003	0.816	350470	2.296E-64	287.360	0.0008
MPV	LAS	rs56316167	T	C	-0.017	0.003	0.419	350470	1.834E-11	48.236	0.0001
MPV	LAS	rs57843631	T	C	0.265	0.009	0.019	350470	8.45E-190	926.422	0.0026
MPV	LAS	rs60757417	G	C	0.115	0.005	0.060	350470	4.3E-115	530.535	0.0015
MPV	LAS	rs6105433	G	T	-0.042	0.004	0.136	350470	1.502E-33	146.953	0.0004
MPV	LAS	rs61776822	A	G	0.032	0.002	0.500	350470	1.137E-39	179.244	0.0005
MPV	LAS	rs61896141	C	A	-0.055	0.003	0.190	350470	1.212E-72	325.437	0.0009
MPV	LAS	rs61919382	T	C	-0.041	0.005	0.073	350470	4.457E-19	79.675	0.0002
MPV	LAS	rs62047991	G	A	-0.021	0.003	0.303	350470	2.964E-15	62.354	0.0002
MPV	LAS	rs62116961	C	G	-0.016	0.002	0.377	350470	1.373E-10	41.302	0.0001
MPV	LAS	rs62338220	A	G	-0.027	0.004	0.083	350470	3.918E-10	39.755	0.0001
MPV	LAS	rs655029	A	G	-0.087	0.003	0.708	350470	1E-200	1099.765	0.0031
MPV	LAS	rs671339	G	A	-0.050	0.003	0.737	350470	7.106E-77	344.568	0.001
MPV	LAS	rs6802617	T	C	-0.116	0.003	0.747	350470	1E-200	1797.537	0.0051
MPV	LAS	rs6848819	C	A	-0.070	0.007	0.033	350470	1.177E-25	109.099	0.0003
MPV	LAS	rs691299	T	G	-0.026	0.003	0.692	350470	6.699E-24	101.535	0.0003
MPV	LAS	rs6993770	T	A	0.046	0.003	0.286	350470	3.243E-67	299.457	0.0009
MPV	LAS	rs7008182	A	T	0.041	0.002	0.490	350470	4.58E-65	290.704	0.0008
MPV	LAS	rs7116797	G	A	-0.031	0.004	0.893	350470	3.176E-15	62.577	0.0002
MPV	LAS	rs72847212	C	T	0.027	0.003	0.202	350470	8.185E-20	83.309	0.0002
MPV	LAS	rs7285107	A	T	-0.024	0.003	0.261	350470	8.076E-18	75.525	0.0002
MPV	LAS	rs73229934	T	C	0.384	0.008	0.024	350470	1E-200	2463.849	0.007

Table S1. SNPs information of platelet indices (PLT, PDW, MPV and PCT) with Stroke and stroke subtypes group (ALS, LAS, SVS and CES)

Exposure	Outcome	SNP	Effect allele	Other allele	Beta	SE	Eaf	N	*P* val	Fval	R2
MPV	LAS	rs73723358	G	A	-0.071	0.004	0.122	350470	7.386E-82	383.992	0.0011
MPV	LAS	rs7427439	G	A	-0.029	0.003	0.748	350470	6.005E-26	111.330	0.0003
MPV	LAS	rs74324636	G	T	-0.082	0.004	0.084	350470	8.584E-81	361.852	0.001
MPV	LAS	rs74505413	G	T	-0.098	0.006	0.043	350470	5.895E-61	273.358	0.0008
MPV	LAS	rs7517721	C	T	-0.040	0.003	0.643	350470	3.172E-56	252.780	0.0007
MPV	LAS	rs7590948	A	G	-0.016	0.002	0.550	350470	3.627E-11	43.952	0.0001
MPV	LAS	rs7700960	C	T	0.051	0.008	0.024	350470	1.272E-10	41.966	0.0001
MPV	LAS	rs78909033	A	G	-0.177	0.003	0.135	350470	1E-200	2594.331	0.0073
MPV	LAS	rs7894089	C	T	0.024	0.003	0.161	350470	1.993E-13	54.113	0.0002
MPV	LAS	rs79548680	C	G	0.044	0.004	0.135	350470	5.216E-36	158.936	0.0005
MPV	LAS	rs80133860	T	G	-0.046	0.007	0.032	350470	3.161E-11	45.656	0.0001
MPV	LAS	rs80250978	T	C	0.037	0.003	0.151	350470	8.498E-28	120.041	0.0003
MPV	LAS	rs8099412	C	T	-0.084	0.002	0.475	350470	1E-200	1247.612	0.0035
MPV	LAS	rs912324	T	A	0.018	0.003	0.743	350470	1.256E-10	41.715	0.0001
MPV	LAS	rs9326600	A	G	-0.105	0.002	0.428	350470	1E-200	1919.135	0.0054
MPV	LAS	rs9368600	G	C	0.021	0.003	0.287	350470	5.083E-15	61.189	0.0002
MPV	LAS	rs9407530	A	G	-0.038	0.003	0.289	350470	9.303E-41	204.890	0.0006
MPV	LAS	rs9549753	A	C	0.048	0.002	0.532	350470	6.145E-88	396.258	0.0011
MPV	LAS	rs972577	T	C	0.061	0.002	0.552	350470	1.17E-144	655.297	0.0019
MPV	LAS	rs976552	C	A	-0.020	0.003	0.240	350470	3.198E-12	48.888	0.0001
MPV	LAS	rs9823526	G	C	0.101	0.002	0.541	350470	1E-200	1797.817	0.0051
MPV	LAS	rs9925477	G	T	0.033	0.002	0.468	350470	1.809E-42	192.370	0.0005
MPV	LAS	rs9962297	C	A	0.016	0.002	0.614	350470	5.813E-11	42.952	0.0001
MPV	SVS	rs10140498	G	A	0.027	0.003	0.169	350470	1.199E-17	73.007	0.0002
MPV	SVS	rs10186787	C	T	0.025	0.003	0.692	350470	9.402E-22	92.505	0.0003
MPV	SVS	rs10509742	T	A	-0.066	0.005	0.063	350470	7.943E-35	179.183	0.0005
MPV	SVS	rs10813766	G	T	-0.057	0.003	0.662	350470	8.75E-115	517.135	0.0015
MPV	SVS	rs10954750	G	C	0.029	0.003	0.789	350470	1.138E-22	98.605	0.0003
MPV	SVS	rs10995445	T	A	-0.093	0.002	0.472	350470	1E-200	1502.703	0.0043
MPV	SVS	rs11064074	T	C	0.101	0.002	0.462	350470	1E-200	1782.627	0.0051
MPV	SVS	rs11071720	C	T	-0.057	0.003	0.702	350470	5.15E-104	469.294	0.0013
MPV	SVS	rs11121529	G	C	0.082	0.004	0.114	350470	3.76E-105	473.407	0.0013
MPV	SVS	rs11170863	T	C	-0.055	0.004	0.131	350470	7.856E-55	245.052	0.0007
MPV	SVS	rs111780165	A	G	-0.044	0.005	0.070	350470	6.473E-21	87.956	0.0003
MPV	SVS	rs114685606	T	A	0.234	0.006	0.046	350470	1E-200	1680.462	0.0048
MPV	SVS	rs114968084	A	G	0.100	0.007	0.030	350470	3.327E-43	202.336	0.0006
MPV	SVS	rs11620922	C	G	-0.042	0.003	0.233	350470	6.808E-50	220.605	0.0006
MPV	SVS	rs11734099	A	G	-0.104	0.003	0.176	350470	1E-200	1111.401	0.0032
MPV	SVS	rs11789898	T	G	-0.053	0.003	0.166	350470	1.654E-61	274.386	0.0008
MPV	SVS	rs11808063	G	A	-0.027	0.003	0.306	350470	1.589E-25	110.964	0.0003
MPV	SVS	rs12232825	G	A	0.103	0.009	0.017	350470	5.999E-29	127.278	0.0004
MPV	SVS	rs12616493	T	C	0.019	0.002	0.556	350470	1.004E-14	60.165	0.0002
MPV	SVS	rs12979891	T	C	-0.029	0.002	0.539	350470	5.721E-34	148.097	0.0004
MPV	SVS	rs138092465	C	G	-0.117	0.003	0.253	350470	1E-200	1831.995	0.0052
MPV	SVS	rs145243487	T	A	-0.112	0.005	0.059	350470	4.38E-106	491.192	0.0014
MPV	SVS	rs146337807	T	C	0.314	0.005	0.054	350470	1E-200	3581.205	0.0101

Table S1. SNPs information of platelet indices (PLT, PDW, MPV and PCT) with Stroke and stroke subtypes group (ALS, LAS, SVS and CES)

Exposure	Outcome	SNP	Effect allele	Other allele	Beta	SE	Eaf	N	*P* val	Fval	R2
MPV	SVS	rs151234	C	G	-0.066	0.004	0.129	350470	9.945E-76	339.683	0.001
MPV	SVS	rs17116384	G	A	0.026	0.003	0.320	350470	1.857E-24	104.935	0.0003
MPV	SVS	rs1736013	C	G	-0.018	0.002	0.432	350470	2.093E-14	58.330	0.0002
MPV	SVS	rs17411126	C	T	-0.020	0.003	0.264	350470	1.474E-13	54.490	0.0002
MPV	SVS	rs1883258	T	G	0.043	0.002	0.384	350470	1.808E-68	306.742	0.0009
MPV	SVS	rs1894572	C	T	0.026	0.002	0.473	350470	6.39E-27	115.140	0.0003
MPV	SVS	rs2038479	A	C	0.133	0.003	0.804	350470	1E-200	1972.345	0.0056
MPV	SVS	rs2079356	T	C	-0.109	0.003	0.398	350470	1E-200	1995.101	0.0057
MPV	SVS	rs2137082	C	G	0.036	0.002	0.519	350470	4.439E-52	230.446	0.0007
MPV	SVS	rs216812	A	G	0.019	0.003	0.323	350470	5.77E-13	53.338	0.0002
MPV	SVS	rs2297066	G	C	-0.078	0.003	0.242	350470	7.14E-171	775.716	0.0022
MPV	SVS	rs2313211	A	T	-0.023	0.002	0.554	350470	6.926E-21	88.422	0.0003
MPV	SVS	rs236691	C	G	-0.078	0.003	0.184	350470	9.4E-140	637.738	0.0018
MPV	SVS	rs2413712	G	A	-0.031	0.003	0.666	350470	3.141E-35	153.908	0.0004
MPV	SVS	rs2516471	A	G	-0.125	0.005	0.063	350470	6.46E-141	649.149	0.0018
MPV	SVS	rs2700982	G	C	0.018	0.002	0.458	350470	2.496E-13	53.897	0.0002
MPV	SVS	rs2709864	A	G	0.048	0.004	0.886	350470	3.977E-38	166.495	0.0005
MPV	SVS	rs2783179	C	T	0.023	0.003	0.368	350470	1.771E-19	83.034	0.0002
MPV	SVS	rs28396651	G	A	0.033	0.002	0.601	350470	3.927E-42	184.813	0.0005
MPV	SVS	rs28633508	T	C	0.028	0.003	0.313	350470	2.53E-26	114.426	0.0003
MPV	SVS	rs3135064	C	T	-0.020	0.002	0.547	350470	1.601E-17	72.904	0.0002
MPV	SVS	rs34379299	G	A	-0.027	0.003	0.195	350470	4.097E-19	80.094	0.0002
MPV	SVS	rs346738	C	T	-0.056	0.003	0.655	350470	2.33E-108	497.273	0.0014
MPV	SVS	rs34991606	T	C	-0.052	0.003	0.140	350470	2.161E-51	227.537	0.0006
MPV	SVS	rs35554791	G	A	-0.064	0.005	0.062	350470	1.198E-37	166.881	0.0005
MPV	SVS	rs35740053	A	C	0.019	0.003	0.206	350470	7.903E-11	42.203	0.0001
MPV	SVS	rs367990687	T	A	-0.142	0.002	0.489	350470	1E-200	3574.165	0.0101
MPV	SVS	rs3740995	C	T	-0.035	0.003	0.846	350470	1.761E-26	114.168	0.0003
MPV	SVS	rs3752112	G	T	-0.026	0.003	0.186	350470	6.422E-17	69.991	0.0002
MPV	SVS	rs3770781	G	A	-0.035	0.002	0.441	350470	8.985E-49	217.109	0.0006
MPV	SVS	rs3826508	C	G	-0.053	0.005	0.070	350470	6.759E-30	129.247	0.0004
MPV	SVS	rs3937015	C	T	-0.134	0.009	0.981	350470	6.081E-53	236.360	0.0007
MPV	SVS	rs4814779	A	C	-0.077	0.003	0.266	350470	1.61E-177	806.807	0.0023
MPV	SVS	rs4941565	G	T	-0.032	0.003	0.652	350470	3.056E-37	162.127	0.0005
MPV	SVS	rs55809585	G	A	-0.052	0.003	0.816	350470	2.296E-64	287.360	0.0008
MPV	SVS	rs56316167	T	C	-0.017	0.003	0.419	350470	1.834E-11	48.236	0.0001
MPV	SVS	rs57843631	T	C	0.265	0.009	0.019	350470	8.45E-190	926.422	0.0026
MPV	SVS	rs60757417	G	C	0.115	0.005	0.060	350470	4.3E-115	530.535	0.0015
MPV	SVS	rs6105433	G	T	-0.042	0.004	0.136	350470	1.502E-33	146.953	0.0004
MPV	SVS	rs61776822	A	G	0.032	0.002	0.500	350470	1.137E-39	179.244	0.0005
MPV	SVS	rs61896141	C	A	-0.055	0.003	0.190	350470	1.212E-72	325.437	0.0009
MPV	SVS	rs61919382	T	C	-0.041	0.005	0.073	350470	4.457E-19	79.675	0.0002
MPV	SVS	rs62047991	G	A	-0.021	0.003	0.303	350470	2.964E-15	62.354	0.0002
MPV	SVS	rs62116961	C	G	-0.016	0.002	0.377	350470	1.373E-10	41.302	0.0001
MPV	SVS	rs62338220	A	G	-0.027	0.004	0.083	350470	3.918E-10	39.755	0.0001
MPV	SVS	rs655029	A	G	-0.087	0.003	0.708	350470	1E-200	1099.765	0.0031

Table S1. SNPs information of platelet indices (PLT, PDW, MPV and PCT) with Stroke and stroke subtypes group (ALS, LAS, SVS and CES)

Exposure	Outcome	SNP	Effect allele	Other allele	Beta	SE	Eaf	N	*P* val	Fval	R2
MPV	SVS	rs671339	G	A	-0.050	0.003	0.737	350470	7.106E-77	344.568	0.001
MPV	SVS	rs6802617	T	C	-0.116	0.003	0.747	350470	1E-200	1797.537	0.0051
MPV	SVS	rs6848819	C	A	-0.070	0.007	0.033	350470	1.177E-25	109.099	0.0003
MPV	SVS	rs691299	T	G	-0.026	0.003	0.692	350470	6.699E-24	101.535	0.0003
MPV	SVS	rs6993770	T	A	0.046	0.003	0.286	350470	3.243E-67	299.457	0.0009
MPV	SVS	rs7008182	A	T	0.041	0.002	0.490	350470	4.58E-65	290.704	0.0008
MPV	SVS	rs7116797	G	A	-0.031	0.004	0.893	350470	3.176E-15	62.577	0.0002
MPV	SVS	rs72847212	C	T	0.027	0.003	0.202	350470	8.185E-20	83.309	0.0002
MPV	SVS	rs7285107	A	T	-0.024	0.003	0.261	350470	8.076E-18	75.525	0.0002
MPV	SVS	rs73229934	T	C	0.384	0.008	0.024	350470	1E-200	2463.849	0.007
MPV	SVS	rs73723358	G	A	-0.071	0.004	0.122	350470	7.386E-82	383.992	0.0011
MPV	SVS	rs7427439	G	A	-0.029	0.003	0.748	350470	6.005E-26	111.330	0.0003
MPV	SVS	rs74324636	G	T	-0.082	0.004	0.084	350470	8.584E-81	361.852	0.001
MPV	SVS	rs74505413	G	T	-0.098	0.006	0.043	350470	5.895E-61	273.358	0.0008
MPV	SVS	rs7517721	C	T	-0.040	0.003	0.643	350470	3.172E-56	252.780	0.0007
MPV	SVS	rs7590948	A	G	-0.016	0.002	0.550	350470	3.627E-11	43.952	0.0001
MPV	SVS	rs7700960	C	T	0.051	0.008	0.024	350470	1.272E-10	41.966	0.0001
MPV	SVS	rs78909033	A	G	-0.177	0.003	0.135	350470	1E-200	2594.331	0.0073
MPV	SVS	rs7894089	C	T	0.024	0.003	0.161	350470	1.993E-13	54.113	0.0002
MPV	SVS	rs79548680	C	G	0.044	0.004	0.135	350470	5.216E-36	158.936	0.0005
MPV	SVS	rs80133860	T	G	-0.046	0.007	0.032	350470	3.161E-11	45.656	0.0001
MPV	SVS	rs80250978	T	C	0.037	0.003	0.151	350470	8.498E-28	120.041	0.0003
MPV	SVS	rs8099412	C	T	-0.084	0.002	0.475	350470	1E-200	1247.612	0.0035
MPV	SVS	rs912324	T	A	0.018	0.003	0.743	350470	1.256E-10	41.715	0.0001
MPV	SVS	rs9326600	A	G	-0.105	0.002	0.428	350470	1E-200	1919.135	0.0054
MPV	SVS	rs9368600	G	C	0.021	0.003	0.287	350470	5.083E-15	61.189	0.0002
MPV	SVS	rs9407530	A	G	-0.038	0.003	0.289	350470	9.303E-41	204.890	0.0006
MPV	SVS	rs9549753	A	C	0.048	0.002	0.532	350470	6.145E-88	396.258	0.0011
MPV	SVS	rs972577	T	C	0.061	0.002	0.552	350470	1.17E-144	655.297	0.0019
MPV	SVS	rs976552	C	A	-0.020	0.003	0.240	350470	3.198E-12	48.888	0.0001
MPV	SVS	rs9823526	G	C	0.101	0.002	0.541	350470	1E-200	1797.817	0.0051
MPV	CES	rs10140498	G	A	0.027	0.003	0.169	350470	1.199E-17	73.007	0.0002
MPV	CES	rs10186787	C	T	0.025	0.003	0.692	350470	9.402E-22	92.505	0.0003
MPV	CES	rs10509742	T	A	-0.066	0.005	0.063	350470	7.943E-35	179.183	0.0005
MPV	CES	rs10813766	G	T	-0.057	0.003	0.662	350470	8.75E-115	517.135	0.0015
MPV	CES	rs10954750	G	C	0.029	0.003	0.789	350470	1.138E-22	98.605	0.0003
MPV	CES	rs10995445	T	A	-0.093	0.002	0.472	350470	1E-200	1502.703	0.0043
MPV	CES	rs11064074	T	C	0.101	0.002	0.462	350470	1E-200	1782.627	0.0051
MPV	CES	rs11071720	C	T	-0.057	0.003	0.702	350470	5.15E-104	469.294	0.0013
MPV	CES	rs11121529	G	C	0.082	0.004	0.114	350470	3.76E-105	473.407	0.0013
MPV	CES	rs11170863	T	C	-0.055	0.004	0.131	350470	7.856E-55	245.052	0.0007
MPV	CES	rs111780165	A	G	-0.044	0.005	0.070	350470	6.473E-21	87.956	0.0003
MPV	CES	rs114685606	T	A	0.234	0.006	0.046	350470	1E-200	1680.462	0.0048
MPV	CES	rs114968084	A	G	0.100	0.007	0.030	350470	3.327E-43	202.336	0.0006
MPV	CES	rs11620922	C	G	-0.042	0.003	0.233	350470	6.808E-50	220.605	0.0006
MPV	CES	rs11734099	A	G	-0.104	0.003	0.176	350470	1E-200	1111.401	0.0032

Table S1. SNPs information of platelet indices (PLT, PDW, MPV and PCT) with Stroke and stroke subtypes group (ALS, LAS, SVS and CES)

Exposure	Outcome	SNP	Effect allele	Other allele	Beta	SE	Eaf	N	*P* val	Fval	R2
MPV	CES	rs11789898	T	G	-0.053	0.003	0.166	350470	1.654E-61	274.386	0.0008
MPV	CES	rs11808063	G	A	-0.027	0.003	0.306	350470	1.589E-25	110.964	0.0003
MPV	CES	rs12232825	G	A	0.103	0.009	0.017	350470	5.999E-29	127.278	0.0004
MPV	CES	rs12616493	T	C	0.019	0.002	0.556	350470	1.004E-14	60.165	0.0002
MPV	CES	rs12979891	T	C	-0.029	0.002	0.539	350470	5.721E-34	148.097	0.0004
MPV	CES	rs138092465	C	G	-0.117	0.003	0.253	350470	1E-200	1831.995	0.0052
MPV	CES	rs145243487	T	A	-0.112	0.005	0.059	350470	4.38E-106	491.192	0.0014
MPV	CES	rs146337807	T	C	0.314	0.005	0.054	350470	1E-200	3581.205	0.0101
MPV	CES	rs151234	C	G	-0.066	0.004	0.129	350470	9.945E-76	339.683	0.001
MPV	CES	rs17116384	G	A	0.026	0.003	0.320	350470	1.857E-24	104.935	0.0003
MPV	CES	rs1736013	C	G	-0.018	0.002	0.432	350470	2.093E-14	58.330	0.0002
MPV	CES	rs17411126	C	T	-0.020	0.003	0.264	350470	1.474E-13	54.490	0.0002
MPV	CES	rs1883258	T	G	0.043	0.002	0.384	350470	1.808E-68	306.742	0.0009
MPV	CES	rs1894572	C	T	0.026	0.002	0.473	350470	6.39E-27	115.140	0.0003
MPV	CES	rs2038479	A	C	0.133	0.003	0.804	350470	1E-200	1972.345	0.0056
MPV	CES	rs2079356	T	C	-0.109	0.003	0.398	350470	1E-200	1995.101	0.0057
MPV	CES	rs2137082	C	G	0.036	0.002	0.519	350470	4.439E-52	230.446	0.0007
MPV	CES	rs216812	A	G	0.019	0.003	0.323	350470	5.77E-13	53.338	0.0002
MPV	CES	rs2297066	G	C	-0.078	0.003	0.242	350470	7.14E-171	775.716	0.0022
MPV	CES	rs2313211	A	T	-0.023	0.002	0.554	350470	6.926E-21	88.422	0.0003
MPV	CES	rs236691	C	G	-0.078	0.003	0.184	350470	9.4E-140	637.738	0.0018
MPV	CES	rs2413712	G	A	-0.031	0.003	0.666	350470	3.141E-35	153.908	0.0004
MPV	CES	rs2516471	A	G	-0.125	0.005	0.063	350470	6.46E-141	649.149	0.0018
MPV	CES	rs2700982	G	C	0.018	0.002	0.458	350470	2.496E-13	53.897	0.0002
MPV	CES	rs2709864	A	G	0.048	0.004	0.886	350470	3.977E-38	166.495	0.0005
MPV	CES	rs2783179	C	T	0.023	0.003	0.368	350470	1.771E-19	83.034	0.0002
MPV	CES	rs28396651	G	A	0.033	0.002	0.601	350470	3.927E-42	184.813	0.0005
MPV	CES	rs28633508	T	C	0.028	0.003	0.313	350470	2.53E-26	114.426	0.0003
MPV	CES	rs3135064	C	T	-0.020	0.002	0.547	350470	1.601E-17	72.904	0.0002
MPV	CES	rs34379299	G	A	-0.027	0.003	0.195	350470	4.097E-19	80.094	0.0002
MPV	CES	rs346738	C	T	-0.056	0.003	0.655	350470	2.33E-108	497.273	0.0014
MPV	CES	rs34991606	T	C	-0.052	0.003	0.140	350470	2.161E-51	227.537	0.0006
MPV	CES	rs35554791	G	A	-0.064	0.005	0.062	350470	1.198E-37	166.881	0.0005
MPV	CES	rs35740053	A	C	0.019	0.003	0.206	350470	7.903E-11	42.203	0.0001
MPV	CES	rs367990687	T	A	-0.142	0.002	0.489	350470	1E-200	3574.165	0.0101
MPV	CES	rs3740995	C	T	-0.035	0.003	0.846	350470	1.761E-26	114.168	0.0003
MPV	CES	rs3752112	G	T	-0.026	0.003	0.186	350470	6.422E-17	69.991	0.0002
MPV	CES	rs3770781	G	A	-0.035	0.002	0.441	350470	8.985E-49	217.109	0.0006
MPV	CES	rs3826508	C	G	-0.053	0.005	0.070	350470	6.759E-30	129.247	0.0004
MPV	CES	rs3937015	C	T	-0.134	0.009	0.981	350470	6.081E-53	236.360	0.0007
MPV	CES	rs4814779	A	C	-0.077	0.003	0.266	350470	1.61E-177	806.807	0.0023
MPV	CES	rs4941565	G	T	-0.032	0.003	0.652	350470	3.056E-37	162.127	0.0005
MPV	CES	rs55809585	G	A	-0.052	0.003	0.816	350470	2.296E-64	287.360	0.0008
MPV	CES	rs56316167	T	C	-0.017	0.003	0.419	350470	1.834E-11	48.236	0.0001
MPV	CES	rs57843631	T	C	0.265	0.009	0.019	350470	8.45E-190	926.422	0.0026
MPV	CES	rs60757417	G	C	0.115	0.005	0.060	350470	4.3E-115	530.535	0.0015

Table S1. SNPs information of platelet indices (PLT, PDW, MPV and PCT) with Stroke and stroke subtypes group (ALS, LAS, SVS and CES)

Exposure	Outcome	SNP	Effect allele	Other allele	Beta	SE	Eaf	N	*P* val	Fval	R2
MPV	CES	rs6105433	G	T	-0.042	0.004	0.136	350470	1.502E-33	146.953	0.0004
MPV	CES	rs61776822	A	G	0.032	0.002	0.500	350470	1.137E-39	179.244	0.0005
MPV	CES	rs61896141	C	A	-0.055	0.003	0.190	350470	1.212E-72	325.437	0.0009
MPV	CES	rs61919382	T	C	-0.041	0.005	0.073	350470	4.457E-19	79.675	0.0002
MPV	CES	rs62047991	G	A	-0.021	0.003	0.303	350470	2.964E-15	62.354	0.0002
MPV	CES	rs62116961	C	G	-0.016	0.002	0.377	350470	1.373E-10	41.302	0.0001
MPV	CES	rs62338220	A	G	-0.027	0.004	0.083	350470	3.918E-10	39.755	0.0001
MPV	CES	rs655029	A	G	-0.087	0.003	0.708	350470	1E-200	1099.765	0.0031
MPV	CES	rs671339	G	A	-0.050	0.003	0.737	350470	7.106E-77	344.568	0.001
MPV	CES	rs6802617	T	C	-0.116	0.003	0.747	350470	1E-200	1797.537	0.0051
MPV	CES	rs6848819	C	A	-0.070	0.007	0.033	350470	1.177E-25	109.099	0.0003
MPV	CES	rs691299	T	G	-0.026	0.003	0.692	350470	6.699E-24	101.535	0.0003
MPV	CES	rs6993770	T	A	0.046	0.003	0.286	350470	3.243E-67	299.457	0.0009
MPV	CES	rs7008182	A	T	0.041	0.002	0.490	350470	4.58E-65	290.704	0.0008
MPV	CES	rs7116797	G	A	-0.031	0.004	0.893	350470	3.176E-15	62.577	0.0002
MPV	CES	rs72847212	C	T	0.027	0.003	0.202	350470	8.185E-20	83.309	0.0002
MPV	CES	rs7285107	A	T	-0.024	0.003	0.261	350470	8.076E-18	75.525	0.0002
MPV	CES	rs73229934	T	C	0.384	0.008	0.024	350470	1E-200	2463.849	0.007
MPV	CES	rs73723358	G	A	-0.071	0.004	0.122	350470	7.386E-82	383.992	0.0011
MPV	CES	rs7427439	G	A	-0.029	0.003	0.748	350470	6.005E-26	111.330	0.0003
MPV	CES	rs74324636	G	T	-0.082	0.004	0.084	350470	8.584E-81	361.852	0.001
MPV	CES	rs74505413	G	T	-0.098	0.006	0.043	350470	5.895E-61	273.358	0.0008
MPV	CES	rs7517721	C	T	-0.040	0.003	0.643	350470	3.172E-56	252.780	0.0007
MPV	CES	rs7590948	A	G	-0.016	0.002	0.550	350470	3.627E-11	43.952	0.0001
MPV	CES	rs7700960	C	T	0.051	0.008	0.024	350470	1.272E-10	41.966	0.0001
MPV	CES	rs7894089	C	T	0.024	0.003	0.161	350470	1.993E-13	54.113	0.0002
MPV	CES	rs79548680	C	G	0.044	0.004	0.135	350470	5.216E-36	158.936	0.0005
MPV	CES	rs80133860	T	G	-0.046	0.007	0.032	350470	3.161E-11	45.656	0.0001
MPV	CES	rs80250978	T	C	0.037	0.003	0.151	350470	8.498E-28	120.041	0.0003
MPV	CES	rs8099412	C	T	-0.084	0.002	0.475	350470	1E-200	1247.612	0.0035
MPV	CES	rs912324	T	A	0.018	0.003	0.743	350470	1.256E-10	41.715	0.0001
MPV	CES	rs9326600	A	G	-0.105	0.002	0.428	350470	1E-200	1919.135	0.0054
MPV	CES	rs9368600	G	C	0.021	0.003	0.287	350470	5.083E-15	61.189	0.0002
MPV	CES	rs9407530	A	G	-0.038	0.003	0.289	350470	9.303E-41	204.890	0.0006
MPV	CES	rs9549753	A	C	0.048	0.002	0.532	350470	6.145E-88	396.258	0.0011
MPV	CES	rs972577	T	C	0.061	0.002	0.552	350470	1.17E-144	655.297	0.0019
MPV	CES	rs976552	C	A	-0.020	0.003	0.240	350470	3.198E-12	48.888	0.0001
MPV	CES	rs9823526	G	C	0.101	0.002	0.541	350470	1E-200	1797.817	0.0051
MPV	CES	rs9925477	G	T	0.033	0.002	0.468	350470	1.809E-42	192.370	0.0005
MPV	CES	rs9962297	C	A	0.016	0.002	0.614	350470	5.813E-11	42.952	0.0001
PDW	Stroke	rs1002707	T	C	0.040	0.003	0.250	350470	2.054E-49	214.106	0.0006
PDW	Stroke	rs10067280	T	C	-0.026	0.002	0.644	350470	3.093E-25	105.535	0.0003
PDW	Stroke	rs10077980	T	C	-0.033	0.003	0.252	350470	6.281E-34	144.845	0.0004
PDW	Stroke	rs10820606	C	A	-0.060	0.003	0.228	350470	1.731E-96	438.685	0.0013
PDW	Stroke	rs10914053	T	C	0.145	0.003	0.826	350470	1E-200	2126.952	0.006
PDW	Stroke	rs11013176	G	A	-0.023	0.003	0.281	350470	1.618E-18	76.263	0.0002

Table S1. SNPs information of platelet indices (PLT, PDW, MPV and PCT) with Stroke and stroke subtypes group (ALS, LAS, SVS and CES)

Exposure	Outcome	SNP	Effect allele	Other allele	Beta	SE	Eaf	N	*P* val	Fval	R2
PDW	Stroke	rs11121012	G	A	-0.026	0.002	0.646	350470	6.957E-26	109.554	0.0003
PDW	Stroke	rs114694170	C	T	-0.119	0.005	0.059	350470	1.42E-122	553.713	0.0016
PDW	Stroke	rs11497367	A	G	0.123	0.006	0.042	350470	6.324E-97	427.909	0.0012
PDW	Stroke	rs11553699	G	A	0.136	0.004	0.135	350470	1E-200	1532.546	0.0044
PDW	Stroke	rs11579514	C	T	-0.020	0.003	0.297	350470	1.309E-14	58.183	0.0002
PDW	Stroke	rs11667509	C	G	0.049	0.002	0.377	350470	1.863E-87	394.414	0.0011
PDW	Stroke	rs11702425	C	T	-0.017	0.003	0.303	350470	4.562E-11	42.724	0.0001
PDW	Stroke	rs11731274	G	T	-0.064	0.003	0.176	350470	1.009E-94	417.890	0.0012
PDW	Stroke	rs11779690	G	A	-0.022	0.002	0.400	350470	4.437E-20	82.624	0.0002
PDW	Stroke	rs11834197	A	G	-0.024	0.002	0.435	350470	1.17E-24	102.902	0.0003
PDW	Stroke	rs11852600	G	T	0.017	0.002	0.349	350470	1.193E-11	45.384	0.0001
PDW	Stroke	rs12166430	G	A	-0.034	0.004	0.098	350470	2.696E-17	70.839	0.0002
PDW	Stroke	rs12616493	T	C	0.019	0.002	0.556	350470	4.242E-16	65.077	0.0002
PDW	Stroke	rs12640722	T	A	0.023	0.003	0.700	350470	2.281E-18	74.993	0.0002
PDW	Stroke	rs12666334	T	G	0.095	0.002	0.421	350470	1E-200	1559.970	0.0044
PDW	Stroke	rs1344236	G	A	0.019	0.003	0.285	350470	1.242E-12	49.268	0.0001
PDW	Stroke	rs1385742	T	A	0.034	0.002	0.647	350470	3.657E-42	184.817	0.0005
PDW	Stroke	rs1530455	C	T	0.038	0.002	0.597	350470	2.229E-55	245.418	0.0007
PDW	Stroke	rs1719285	A	T	-0.085	0.003	0.157	350470	1.07E-152	678.050	0.0019
PDW	Stroke	rs1797077	T	C	0.034	0.002	0.480	350470	8.019E-47	203.257	0.0006
PDW	Stroke	rs1980532	G	A	-0.056	0.002	0.447	350470	1.47E-121	543.750	0.0015
PDW	Stroke	rs2228367	C	T	0.218	0.009	0.019	350470	2.07E-131	610.180	0.0017
PDW	Stroke	rs2810490	A	G	-0.018	0.003	0.284	350470	5.4E-12	46.461	0.0001
PDW	Stroke	rs2876139	C	A	0.033	0.002	0.638	350470	5.623E-40	172.027	0.0005
PDW	Stroke	rs34834842	G	A	0.103	0.006	0.038	350470	6.417E-62	270.379	0.0008
PDW	Stroke	rs3748136	A	G	0.087	0.003	0.211	350470	3.73E-198	883.945	0.0025
PDW	Stroke	rs3761260	A	G	0.158	0.003	0.138	350470	1E-200	2080.684	0.0059
PDW	Stroke	rs4775609	G	T	-0.023	0.002	0.509	350470	3.357E-22	91.992	0.0003
PDW	Stroke	rs4783186	C	T	0.057	0.004	0.876	350470	3.44E-57	247.929	0.0007
PDW	Stroke	rs4790718	A	G	-0.089	0.005	0.064	350470	2.11E-75	331.072	0.0009
PDW	Stroke	rs4904924	A	G	-0.022	0.003	0.797	350470	1.925E-13	52.907	0.0002
PDW	Stroke	rs4952073	C	T	-0.116	0.003	0.703	350470	1E-200	1980.074	0.0056
PDW	Stroke	rs540909	C	T	0.052	0.003	0.754	350470	1.395E-80	352.189	0.001
PDW	Stroke	rs56050846	A	G	-0.024	0.003	0.242	350470	3.135E-18	74.585	0.0002
PDW	Stroke	rs565176201	G	A	0.031	0.003	0.710	350470	4.335E-31	137.726	0.0004
PDW	Stroke	rs56956502	A	G	-0.034	0.003	0.248	350470	1.997E-35	151.198	0.0004
PDW	Stroke	rs5758968	C	T	0.089	0.002	0.492	350470	1E-200	1401.958	0.004
PDW	Stroke	rs606552	G	A	0.060	0.003	0.247	350470	7.4E-107	476.631	0.0014
PDW	Stroke	rs60696641	A	G	0.126	0.004	0.079	350470	5.62E-182	812.314	0.0023
PDW	Stroke	rs6070629	T	G	0.033	0.003	0.763	350470	1.276E-32	140.511	0.0004
PDW	Stroke	rs609311	A	C	-0.031	0.003	0.157	350470	4.241E-21	90.895	0.0003
PDW	Stroke	rs61751937	C	G	0.082	0.007	0.029	350470	1.712E-31	133.043	0.0004
PDW	Stroke	rs61838222	A	G	-0.025	0.002	0.622	350470	7.343E-25	104.345	0.0003
PDW	Stroke	rs6533483	A	G	0.020	0.002	0.402	350470	6.009E-16	64.631	0.0002
PDW	Stroke	rs6558407	T	C	-0.048	0.002	0.424	350470	1.953E-91	402.604	0.0011
PDW	Stroke	rs671339	G	A	-0.039	0.003	0.737	350470	8.226E-48	206.804	0.0006

Table S1. SNPs information of platelet indices (PLT, PDW, MPV and PCT) with Stroke and stroke subtypes group (ALS, LAS, SVS and CES)

Exposure	Outcome	SNP	Effect allele	Other allele	Beta	SE	Eaf	N	*P* val	Fval	R2
PDW	Stroke	rs6772399	T	C	0.023	0.003	0.225	350470	2.179E-16	66.920	0.0002
PDW	Stroke	rs6900762	G	A	0.036	0.003	0.781	350470	1.219E-36	156.813	0.0004
PDW	Stroke	rs6953107	T	C	-0.073	0.003	0.197	350470	3.67E-134	598.817	0.0017
PDW	Stroke	rs7192755	G	T	0.021	0.003	0.253	350470	8.722E-15	58.850	0.0002
PDW	Stroke	rs7235010	A	G	0.030	0.003	0.784	350470	2.027E-25	105.988	0.0003
PDW	Stroke	rs72699866	A	G	0.026	0.003	0.191	350470	2.053E-17	70.830	0.0002
PDW	Stroke	rs727088	A	G	0.053	0.002	0.525	350470	1.56E-109	484.161	0.0014
PDW	Stroke	rs7503168	G	A	-0.104	0.003	0.172	350470	1E-200	1083.408	0.0031
PDW	Stroke	rs752806	T	A	0.020	0.002	0.522	350470	2.053E-17	71.610	0.0002
PDW	Stroke	rs7574937	T	C	0.069	0.004	0.097	350470	1.889E-67	294.818	0.0008
PDW	Stroke	rs7640885	C	A	-0.023	0.003	0.668	350470	5.961E-20	83.067	0.0002
PDW	Stroke	rs7649970	T	C	-0.026	0.004	0.121	350470	8.927E-13	49.746	0.0001
PDW	Stroke	rs7696969	G	T	-0.037	0.002	0.619	350470	2.276E-51	222.657	0.0006
PDW	Stroke	rs7701346	G	A	-0.041	0.002	0.573	350470	1.549E-64	286.478	0.0008
PDW	Stroke	rs7711939	C	T	0.018	0.002	0.645	350470	1.666E-13	53.157	0.0002
PDW	Stroke	rs7719425	C	T	0.027	0.003	0.203	350470	5.276E-20	82.969	0.0002
PDW	Stroke	rs7768878	A	G	0.046	0.007	0.028	350470	2.209E-10	39.398	0.0001
PDW	Stroke	rs7769393	G	A	-0.048	0.003	0.216	350470	1.697E-63	276.686	0.0008
PDW	Stroke	rs7830388	G	T	0.034	0.002	0.454	350470	2.102E-46	201.148	0.0006
PDW	Stroke	rs7847758	T	C	0.019	0.003	0.784	350470	1.4E-10	41.817	0.0001
PDW	Stroke	rs78895525	T	C	-0.036	0.003	0.220	350470	1.149E-35	155.416	0.0004
PDW	Stroke	rs7936105	C	T	-0.017	0.002	0.417	350470	2.023E-12	48.596	0.0001
PDW	Stroke	rs7958679	T	C	0.157	0.005	0.074	350470	1E-200	1188.629	0.0034
PDW	Stroke	rs892090	G	T	0.081	0.003	0.835	350470	1.66E-142	632.072	0.0018
PDW	Stroke	rs941207	G	C	-0.061	0.003	0.268	350470	1.25E-115	510.930	0.0015
PDW	Stroke	rs9534458	A	G	0.061	0.002	0.652	350470	3.37E-132	585.345	0.0017
PDW	Stroke	rs9549753	A	C	0.045	0.002	0.532	350470	1.534E-79	350.291	0.001
PDW	Stroke	rs9636612	G	A	0.026	0.002	0.373	350470	2.306E-26	111.208	0.0003
PDW	Stroke	rs9890401	G	T	0.021	0.002	0.629	350470	2.754E-17	71.474	0.0002
PDW	AIS	rs1002707	T	C	0.040	0.003	0.250	350470	2.054E-49	214.106	0.0006
PDW	AIS	rs10067280	T	C	-0.026	0.002	0.644	350470	3.093E-25	105.535	0.0003
PDW	AIS	rs10077980	T	C	-0.033	0.003	0.252	350470	6.281E-34	144.845	0.0004
PDW	AIS	rs10820606	C	A	-0.060	0.003	0.228	350470	1.731E-96	438.685	0.0013
PDW	AIS	rs10914053	T	C	0.145	0.003	0.826	350470	1E-200	2126.952	0.006
PDW	AIS	rs11013176	G	A	-0.023	0.003	0.281	350470	1.618E-18	76.263	0.0002
PDW	AIS	rs11121012	G	A	-0.026	0.002	0.646	350470	6.957E-26	109.554	0.0003
PDW	AIS	rs114694170	C	T	-0.119	0.005	0.059	350470	1.42E-122	553.713	0.0016
PDW	AIS	rs11497367	A	G	0.123	0.006	0.042	350470	6.324E-97	427.909	0.0012
PDW	AIS	rs11553699	G	A	0.136	0.004	0.135	350470	1E-200	1532.546	0.0044
PDW	AIS	rs11579514	C	T	-0.020	0.003	0.297	350470	1.309E-14	58.183	0.0002
PDW	AIS	rs11667509	C	G	0.049	0.002	0.377	350470	1.863E-87	394.414	0.0011
PDW	AIS	rs11702425	C	T	-0.017	0.003	0.303	350470	4.562E-11	42.724	0.0001
PDW	AIS	rs11731274	G	T	-0.064	0.003	0.176	350470	1.009E-94	417.890	0.0012
PDW	AIS	rs11779690	G	A	-0.022	0.002	0.400	350470	4.437E-20	82.624	0.0002
PDW	AIS	rs11834197	A	G	-0.024	0.002	0.435	350470	1.17E-24	102.902	0.0003
PDW	AIS	rs11852600	G	T	0.017	0.002	0.349	350470	1.193E-11	45.384	0.0001

Table S1. SNPs information of platelet indices (PLT, PDW, MPV and PCT) with Stroke and stroke subtypes group (ALS, LAS, SVS and CES)

Exposure	Outcome	SNP	Effect allele	Other allele	Beta	SE	Eaf	N	*P* val	Fval	R2
PDW	AIS	rs12166430	G	A	-0.034	0.004	0.098	350470	2.696E-17	70.839	0.0002
PDW	AIS	rs12616493	T	C	0.019	0.002	0.556	350470	4.242E-16	65.077	0.0002
PDW	AIS	rs12640722	T	A	0.023	0.003	0.700	350470	2.281E-18	74.993	0.0002
PDW	AIS	rs12666334	T	G	0.095	0.002	0.421	350470	1E-200	1559.970	0.0044
PDW	AIS	rs1344236	G	A	0.019	0.003	0.285	350470	1.242E-12	49.268	0.0001
PDW	AIS	rs1385742	T	A	0.034	0.002	0.647	350470	3.657E-42	184.817	0.0005
PDW	AIS	rs1530455	C	T	0.038	0.002	0.597	350470	2.229E-55	245.418	0.0007
PDW	AIS	rs1719285	A	T	-0.085	0.003	0.157	350470	1.07E-152	678.050	0.0019
PDW	AIS	rs1797077	T	C	0.034	0.002	0.480	350470	8.019E-47	203.257	0.0006
PDW	AIS	rs1980532	G	A	-0.056	0.002	0.447	350470	1.47E-121	543.750	0.0015
PDW	AIS	rs2228367	C	T	0.218	0.009	0.019	350470	2.07E-131	610.180	0.0017
PDW	AIS	rs2810490	A	G	-0.018	0.003	0.284	350470	5.4E-12	46.461	0.0001
PDW	AIS	rs2876139	C	A	0.033	0.002	0.638	350470	5.623E-40	172.027	0.0005
PDW	AIS	rs34834842	G	A	0.103	0.006	0.038	350470	6.417E-62	270.379	0.0008
PDW	AIS	rs3748136	A	G	0.087	0.003	0.211	350470	3.73E-198	883.945	0.0025
PDW	AIS	rs3761260	A	G	0.158	0.003	0.138	350470	1E-200	2080.684	0.0059
PDW	AIS	rs4775609	G	T	-0.023	0.002	0.509	350470	3.357E-22	91.992	0.0003
PDW	AIS	rs4783186	C	T	0.057	0.004	0.876	350470	3.44E-57	247.929	0.0007
PDW	AIS	rs4790718	A	G	-0.089	0.005	0.064	350470	2.11E-75	331.072	0.0009
PDW	AIS	rs4904924	A	G	-0.022	0.003	0.797	350470	1.925E-13	52.907	0.0002
PDW	AIS	rs4952073	C	T	-0.116	0.003	0.703	350470	1E-200	1980.074	0.0056
PDW	AIS	rs540909	C	T	0.052	0.003	0.754	350470	1.395E-80	352.189	0.001
PDW	AIS	rs56050846	A	G	-0.024	0.003	0.242	350470	3.135E-18	74.585	0.0002
PDW	AIS	rs565176201	G	A	0.031	0.003	0.710	350470	4.335E-31	137.726	0.0004
PDW	AIS	rs56956502	A	G	-0.034	0.003	0.248	350470	1.997E-35	151.198	0.0004
PDW	AIS	rs5758968	C	T	0.089	0.002	0.492	350470	1E-200	1401.958	0.004
PDW	AIS	rs606552	G	A	0.060	0.003	0.247	350470	7.4E-107	476.631	0.0014
PDW	AIS	rs60696641	A	G	0.126	0.004	0.079	350470	5.62E-182	812.314	0.0023
PDW	AIS	rs6070629	T	G	0.033	0.003	0.763	350470	1.276E-32	140.511	0.0004
PDW	AIS	rs609311	A	C	-0.031	0.003	0.157	350470	4.241E-21	90.895	0.0003
PDW	AIS	rs61751937	C	G	0.082	0.007	0.029	350470	1.712E-31	133.043	0.0004
PDW	AIS	rs61838222	A	G	-0.025	0.002	0.622	350470	7.343E-25	104.345	0.0003
PDW	AIS	rs6533483	A	G	0.020	0.002	0.402	350470	6.009E-16	64.631	0.0002
PDW	AIS	rs6558407	T	C	-0.048	0.002	0.424	350470	1.953E-91	402.604	0.0011
PDW	AIS	rs671339	G	A	-0.039	0.003	0.737	350470	8.226E-48	206.804	0.0006
PDW	AIS	rs6772399	T	C	0.023	0.003	0.225	350470	2.179E-16	66.920	0.0002
PDW	AIS	rs6900762	G	A	0.036	0.003	0.781	350470	1.219E-36	156.813	0.0004
PDW	AIS	rs6953107	T	C	-0.073	0.003	0.197	350470	3.67E-134	598.817	0.0017
PDW	AIS	rs7192755	G	T	0.021	0.003	0.253	350470	8.722E-15	58.850	0.0002
PDW	AIS	rs7235010	A	G	0.030	0.003	0.784	350470	2.027E-25	105.988	0.0003
PDW	AIS	rs72699866	A	G	0.026	0.003	0.191	350470	2.053E-17	70.830	0.0002
PDW	AIS	rs727088	A	G	0.053	0.002	0.525	350470	1.56E-109	484.161	0.0014
PDW	AIS	rs7503168	G	A	-0.104	0.003	0.172	350470	1E-200	1083.408	0.0031
PDW	AIS	rs752806	T	A	0.020	0.002	0.522	350470	2.053E-17	71.610	0.0002
PDW	AIS	rs7574937	T	C	0.069	0.004	0.097	350470	1.889E-67	294.818	0.0008
PDW	AIS	rs7640885	C	A	-0.023	0.003	0.668	350470	5.961E-20	83.067	0.0002

Table S1. SNPs information of platelet indices (PLT, PDW, MPV and PCT) with Stroke and stroke subtypes group (ALS, LAS, SVS and CES)

Exposure	Outcome	SNP	Effect allele	Other allele	Beta	SE	Eaf	N	*P* val	Fval	R2
PDW	AIS	rs7649970	T	C	-0.026	0.004	0.121	350470	8.927E-13	49.746	0.0001
PDW	AIS	rs7696969	G	T	-0.037	0.002	0.619	350470	2.276E-51	222.657	0.0006
PDW	AIS	rs7701346	G	A	-0.041	0.002	0.573	350470	1.549E-64	286.478	0.0008
PDW	AIS	rs7711939	C	T	0.018	0.002	0.645	350470	1.666E-13	53.157	0.0002
PDW	AIS	rs7719425	C	T	0.027	0.003	0.203	350470	5.276E-20	82.969	0.0002
PDW	AIS	rs7768878	A	G	0.046	0.007	0.028	350470	2.209E-10	39.398	0.0001
PDW	AIS	rs7769393	G	A	-0.048	0.003	0.216	350470	1.697E-63	276.686	0.0008
PDW	AIS	rs7830388	G	T	0.034	0.002	0.454	350470	2.102E-46	201.148	0.0006
PDW	AIS	rs7847758	T	C	0.019	0.003	0.784	350470	1.4E-10	41.817	0.0001
PDW	AIS	rs78895525	T	C	-0.036	0.003	0.220	350470	1.149E-35	155.416	0.0004
PDW	AIS	rs7936105	C	T	-0.017	0.002	0.417	350470	2.023E-12	48.596	0.0001
PDW	AIS	rs7958679	T	C	0.157	0.005	0.074	350470	1E-200	1188.629	0.0034
PDW	AIS	rs892090	G	T	0.081	0.003	0.835	350470	1.66E-142	632.072	0.0018
PDW	AIS	rs941207	G	C	-0.061	0.003	0.268	350470	1.25E-115	510.930	0.0015
PDW	AIS	rs9549753	A	C	0.045	0.002	0.532	350470	1.534E-79	350.291	0.001
PDW	AIS	rs9636612	G	A	0.026	0.002	0.373	350470	2.306E-26	111.208	0.0003
PDW	AIS	rs9890401	G	T	0.021	0.002	0.629	350470	2.754E-17	71.474	0.0002
PDW	LAS	rs1002707	T	C	0.040	0.003	0.250	350470	2.054E-49	214.106	0.0006
PDW	LAS	rs10067280	T	C	-0.026	0.002	0.644	350470	3.093E-25	105.535	0.0003
PDW	LAS	rs10077980	T	C	-0.033	0.003	0.252	350470	6.281E-34	144.845	0.0004
PDW	LAS	rs10820606	C	A	-0.060	0.003	0.228	350470	1.731E-96	438.685	0.0013
PDW	LAS	rs10914053	T	C	0.145	0.003	0.826	350470	1E-200	2126.952	0.006
PDW	LAS	rs11013176	G	A	-0.023	0.003	0.281	350470	1.618E-18	76.263	0.0002
PDW	LAS	rs11121012	G	A	-0.026	0.002	0.646	350470	6.957E-26	109.554	0.0003
PDW	LAS	rs114694170	C	T	-0.119	0.005	0.059	350470	1.42E-122	553.713	0.0016
PDW	LAS	rs11497367	A	G	0.123	0.006	0.042	350470	6.324E-97	427.909	0.0012
PDW	LAS	rs11553699	G	A	0.136	0.004	0.135	350470	1E-200	1532.546	0.0044
PDW	LAS	rs11579514	C	T	-0.020	0.003	0.297	350470	1.309E-14	58.183	0.0002
PDW	LAS	rs11667509	C	G	0.049	0.002	0.377	350470	1.863E-87	394.414	0.0011
PDW	LAS	rs11702425	C	T	-0.017	0.003	0.303	350470	4.562E-11	42.724	0.0001
PDW	LAS	rs11731274	G	T	-0.064	0.003	0.176	350470	1.009E-94	417.890	0.0012
PDW	LAS	rs11779690	G	A	-0.022	0.002	0.400	350470	4.437E-20	82.624	0.0002
PDW	LAS	rs11834197	A	G	-0.024	0.002	0.435	350470	1.17E-24	102.902	0.0003
PDW	LAS	rs11852600	G	T	0.017	0.002	0.349	350470	1.193E-11	45.384	0.0001
PDW	LAS	rs12166430	G	A	-0.034	0.004	0.098	350470	2.696E-17	70.839	0.0002
PDW	LAS	rs12616493	T	C	0.019	0.002	0.556	350470	4.242E-16	65.077	0.0002
PDW	LAS	rs12640722	T	A	0.023	0.003	0.700	350470	2.281E-18	74.993	0.0002
PDW	LAS	rs12666334	T	G	0.095	0.002	0.421	350470	1E-200	1559.970	0.0044
PDW	LAS	rs1344236	G	A	0.019	0.003	0.285	350470	1.242E-12	49.268	0.0001
PDW	LAS	rs1385742	T	A	0.034	0.002	0.647	350470	3.657E-42	184.817	0.0005
PDW	LAS	rs1530455	C	T	0.038	0.002	0.597	350470	2.229E-55	245.418	0.0007
PDW	LAS	rs1719285	A	T	-0.085	0.003	0.157	350470	1.07E-152	678.050	0.0019
PDW	LAS	rs1797077	T	C	0.034	0.002	0.480	350470	8.019E-47	203.257	0.0006
PDW	LAS	rs1980532	G	A	-0.056	0.002	0.447	350470	1.47E-121	543.750	0.0015
PDW	LAS	rs2228367	C	T	0.218	0.009	0.019	350470	2.07E-131	610.180	0.0017
PDW	LAS	rs2810490	A	G	-0.018	0.003	0.284	350470	5.4E-12	46.461	0.0001

Table S1. SNPs information of platelet indices (PLT, PDW, MPV and PCT) with Stroke and stroke subtypes group (ALS, LAS, SVS and CES)

Exposure	Outcome	SNP	Effect allele	Other allele	Beta	SE	Eaf	N	*P* val	Fval	R2
PDW	LAS	rs2876139	C	A	0.033	0.002	0.638	350470	5.623E-40	172.027	0.0005
PDW	LAS	rs34834842	G	A	0.103	0.006	0.038	350470	6.417E-62	270.379	0.0008
PDW	LAS	rs3748136	A	G	0.087	0.003	0.211	350470	3.73E-198	883.945	0.0025
PDW	LAS	rs3761260	A	G	0.158	0.003	0.138	350470	1E-200	2080.684	0.0059
PDW	LAS	rs4775609	G	T	-0.023	0.002	0.509	350470	3.357E-22	91.992	0.0003
PDW	LAS	rs4783186	C	T	0.057	0.004	0.876	350470	3.44E-57	247.929	0.0007
PDW	LAS	rs4790718	A	G	-0.089	0.005	0.064	350470	2.11E-75	331.072	0.0009
PDW	LAS	rs4904924	A	G	-0.022	0.003	0.797	350470	1.925E-13	52.907	0.0002
PDW	LAS	rs4952073	C	T	-0.116	0.003	0.703	350470	1E-200	1980.074	0.0056
PDW	LAS	rs540909	C	T	0.052	0.003	0.754	350470	1.395E-80	352.189	0.001
PDW	LAS	rs56050846	A	G	-0.024	0.003	0.242	350470	3.135E-18	74.585	0.0002
PDW	LAS	rs565176201	G	A	0.031	0.003	0.710	350470	4.335E-31	137.726	0.0004
PDW	LAS	rs56956502	A	G	-0.034	0.003	0.248	350470	1.997E-35	151.198	0.0004
PDW	LAS	rs5758968	C	T	0.089	0.002	0.492	350470	1E-200	1401.958	0.004
PDW	LAS	rs606552	G	A	0.060	0.003	0.247	350470	7.4E-107	476.631	0.0014
PDW	LAS	rs60696641	A	G	0.126	0.004	0.079	350470	5.62E-182	812.314	0.0023
PDW	LAS	rs6070629	T	G	0.033	0.003	0.763	350470	1.276E-32	140.511	0.0004
PDW	LAS	rs609311	A	C	-0.031	0.003	0.157	350470	4.241E-21	90.895	0.0003
PDW	LAS	rs61751937	C	G	0.082	0.007	0.029	350470	1.712E-31	133.043	0.0004
PDW	LAS	rs61838222	A	G	-0.025	0.002	0.622	350470	7.343E-25	104.345	0.0003
PDW	LAS	rs6533483	A	G	0.020	0.002	0.402	350470	6.009E-16	64.631	0.0002
PDW	LAS	rs6558407	T	C	-0.048	0.002	0.424	350470	1.953E-91	402.604	0.0011
PDW	LAS	rs671339	G	A	-0.039	0.003	0.737	350470	8.226E-48	206.804	0.0006
PDW	LAS	rs6772399	T	C	0.023	0.003	0.225	350470	2.179E-16	66.920	0.0002
PDW	LAS	rs6900762	G	A	0.036	0.003	0.781	350470	1.219E-36	156.813	0.0004
PDW	LAS	rs6953107	T	C	-0.073	0.003	0.197	350470	3.67E-134	598.817	0.0017
PDW	LAS	rs7192755	G	T	0.021	0.003	0.253	350470	8.722E-15	58.850	0.0002
PDW	LAS	rs7235010	A	G	0.030	0.003	0.784	350470	2.027E-25	105.988	0.0003
PDW	LAS	rs72699866	A	G	0.026	0.003	0.191	350470	2.053E-17	70.830	0.0002
PDW	LAS	rs727088	A	G	0.053	0.002	0.525	350470	1.56E-109	484.161	0.0014
PDW	LAS	rs7503168	G	A	-0.104	0.003	0.172	350470	1E-200	1083.408	0.0031
PDW	LAS	rs752806	T	A	0.020	0.002	0.522	350470	2.053E-17	71.610	0.0002
PDW	LAS	rs7574937	T	C	0.069	0.004	0.097	350470	1.889E-67	294.818	0.0008
PDW	LAS	rs7640885	C	A	-0.023	0.003	0.668	350470	5.961E-20	83.067	0.0002
PDW	LAS	rs7649970	T	C	-0.026	0.004	0.121	350470	8.927E-13	49.746	0.0001
PDW	LAS	rs7696969	G	T	-0.037	0.002	0.619	350470	2.276E-51	222.657	0.0006
PDW	LAS	rs7701346	G	A	-0.041	0.002	0.573	350470	1.549E-64	286.478	0.0008
PDW	LAS	rs7711939	C	T	0.018	0.002	0.645	350470	1.666E-13	53.157	0.0002
PDW	LAS	rs7719425	C	T	0.027	0.003	0.203	350470	5.276E-20	82.969	0.0002
PDW	LAS	rs7768878	A	G	0.046	0.007	0.028	350470	2.209E-10	39.398	0.0001
PDW	LAS	rs7769393	G	A	-0.048	0.003	0.216	350470	1.697E-63	276.686	0.0008
PDW	LAS	rs7830388	G	T	0.034	0.002	0.454	350470	2.102E-46	201.148	0.0006
PDW	LAS	rs7847758	T	C	0.019	0.003	0.784	350470	1.4E-10	41.817	0.0001
PDW	LAS	rs78895525	T	C	-0.036	0.003	0.220	350470	1.149E-35	155.416	0.0004
PDW	LAS	rs7936105	C	T	-0.017	0.002	0.417	350470	2.023E-12	48.596	0.0001
PDW	LAS	rs7958679	T	C	0.157	0.005	0.074	350470	1E-200	1188.629	0.0034

Table S1. SNPs information of platelet indices (PLT, PDW, MPV and PCT) with Stroke and stroke subtypes group (ALS, LAS, SVS and CES)

Exposure	Outcome	SNP	Effect allele	Other allele	Beta	SE	Eaf	N	*P* val	Fval	R2
PDW	LAS	rs892090	G	T	0.081	0.003	0.835	350470	1.66E-142	632.072	0.0018
PDW	LAS	rs941207	G	C	-0.061	0.003	0.268	350470	1.25E-115	510.930	0.0015
PDW	LAS	rs9549753	A	C	0.045	0.002	0.532	350470	1.534E-79	350.291	0.001
PDW	LAS	rs9636612	G	A	0.026	0.002	0.373	350470	2.306E-26	111.208	0.0003
PDW	LAS	rs9890401	G	T	0.021	0.002	0.629	350470	2.754E-17	71.474	0.0002
PDW	SVS	rs1002707	T	C	0.040	0.003	0.250	350470	2.054E-49	214.106	0.0006
PDW	SVS	rs10067280	T	C	-0.026	0.002	0.644	350470	3.093E-25	105.535	0.0003
PDW	SVS	rs10077980	T	C	-0.033	0.003	0.252	350470	6.281E-34	144.845	0.0004
PDW	SVS	rs10774624	A	G	-0.018	0.002	0.514	350470	1.273E-13	54.718	0.0002
PDW	SVS	rs10820606	C	A	-0.060	0.003	0.228	350470	1.731E-96	438.685	0.0013
PDW	SVS	rs10914053	T	C	0.145	0.003	0.826	350470	1E-200	2126.952	0.006
PDW	SVS	rs11013176	G	A	-0.023	0.003	0.281	350470	1.618E-18	76.263	0.0002
PDW	SVS	rs11121012	G	A	-0.026	0.002	0.646	350470	6.957E-26	109.554	0.0003
PDW	SVS	rs114694170	C	T	-0.119	0.005	0.059	350470	1.42E-122	553.713	0.0016
PDW	SVS	rs11497367	A	G	0.123	0.006	0.042	350470	6.324E-97	427.909	0.0012
PDW	SVS	rs11553699	G	A	0.136	0.004	0.135	350470	1E-200	1532.546	0.0044
PDW	SVS	rs11579514	C	T	-0.020	0.003	0.297	350470	1.309E-14	58.183	0.0002
PDW	SVS	rs11667509	C	G	0.049	0.002	0.377	350470	1.863E-87	394.414	0.0011
PDW	SVS	rs11702425	C	T	-0.017	0.003	0.303	350470	4.562E-11	42.724	0.0001
PDW	SVS	rs11731274	G	T	-0.064	0.003	0.176	350470	1.009E-94	417.890	0.0012
PDW	SVS	rs11779690	G	A	-0.022	0.002	0.400	350470	4.437E-20	82.624	0.0002
PDW	SVS	rs11834197	A	G	-0.024	0.002	0.435	350470	1.17E-24	102.902	0.0003
PDW	SVS	rs11852600	G	T	0.017	0.002	0.349	350470	1.193E-11	45.384	0.0001
PDW	SVS	rs12166430	G	A	-0.034	0.004	0.098	350470	2.696E-17	70.839	0.0002
PDW	SVS	rs12616493	T	C	0.019	0.002	0.556	350470	4.242E-16	65.077	0.0002
PDW	SVS	rs12640722	T	A	0.023	0.003	0.700	350470	2.281E-18	74.993	0.0002
PDW	SVS	rs12666334	T	G	0.095	0.002	0.421	350470	1E-200	1559.970	0.0044
PDW	SVS	rs1344236	G	A	0.019	0.003	0.285	350470	1.242E-12	49.268	0.0001
PDW	SVS	rs1385742	T	A	0.034	0.002	0.647	350470	3.657E-42	184.817	0.0005
PDW	SVS	rs1530455	C	T	0.038	0.002	0.597	350470	2.229E-55	245.418	0.0007
PDW	SVS	rs1719285	A	T	-0.085	0.003	0.157	350470	1.07E-152	678.050	0.0019
PDW	SVS	rs1797077	T	C	0.034	0.002	0.480	350470	8.019E-47	203.257	0.0006
PDW	SVS	rs1980532	G	A	-0.056	0.002	0.447	350470	1.47E-121	543.750	0.0015
PDW	SVS	rs2228367	C	T	0.218	0.009	0.019	350470	2.07E-131	610.180	0.0017
PDW	SVS	rs2810490	A	G	-0.018	0.003	0.284	350470	5.4E-12	46.461	0.0001
PDW	SVS	rs2876139	C	A	0.033	0.002	0.638	350470	5.623E-40	172.027	0.0005
PDW	SVS	rs34834842	G	A	0.103	0.006	0.038	350470	6.417E-62	270.379	0.0008
PDW	SVS	rs3748136	A	G	0.087	0.003	0.211	350470	3.73E-198	883.945	0.0025
PDW	SVS	rs3761260	A	G	0.158	0.003	0.138	350470	1E-200	2080.684	0.0059
PDW	SVS	rs3843751	T	C	0.017	0.003	0.664	350470	3.175E-11	43.181	0.0001
PDW	SVS	rs415895	G	C	0.031	0.002	0.645	350470	3.207E-36	154.656	0.0004
PDW	SVS	rs4775609	G	T	-0.023	0.002	0.509	350470	3.357E-22	91.992	0.0003
PDW	SVS	rs4783186	C	T	0.057	0.004	0.876	350470	3.44E-57	247.929	0.0007
PDW	SVS	rs4790718	A	G	-0.089	0.005	0.064	350470	2.11E-75	331.072	0.0009
PDW	SVS	rs4904924	A	G	-0.022	0.003	0.797	350470	1.925E-13	52.907	0.0002
PDW	SVS	rs4952073	C	T	-0.116	0.003	0.703	350470	1E-200	1980.074	0.0056

Table S1. SNPs information of platelet indices (PLT, PDW, MPV and PCT) with Stroke and stroke subtypes group (ALS, LAS, SVS and CES)

Exposure	Outcome	SNP	Effect allele	Other allele	Beta	SE	Eaf	N	*P* val	Fval	R2
PDW	SVS	rs540909	C	T	0.052	0.003	0.754	350470	1.395E-80	352.189	0.001
PDW	SVS	rs56050846	A	G	-0.024	0.003	0.242	350470	3.135E-18	74.585	0.0002
PDW	SVS	rs565176201	G	A	0.031	0.003	0.710	350470	4.335E-31	137.726	0.0004
PDW	SVS	rs56956502	A	G	-0.034	0.003	0.248	350470	1.997E-35	151.198	0.0004
PDW	SVS	rs5758968	C	T	0.089	0.002	0.492	350470	1E-200	1401.958	0.004
PDW	SVS	rs606552	G	A	0.060	0.003	0.247	350470	7.4E-107	476.631	0.0014
PDW	SVS	rs60696641	A	G	0.126	0.004	0.079	350470	5.62E-182	812.314	0.0023
PDW	SVS	rs6070629	T	G	0.033	0.003	0.763	350470	1.276E-32	140.511	0.0004
PDW	SVS	rs609311	A	C	-0.031	0.003	0.157	350470	4.241E-21	90.895	0.0003
PDW	SVS	rs61751937	C	G	0.082	0.007	0.029	350470	1.712E-31	133.043	0.0004
PDW	SVS	rs61838222	A	G	-0.025	0.002	0.622	350470	7.343E-25	104.345	0.0003
PDW	SVS	rs6533483	A	G	0.020	0.002	0.402	350470	6.009E-16	64.631	0.0002
PDW	SVS	rs6558407	T	C	-0.048	0.002	0.424	350470	1.953E-91	402.604	0.0011
PDW	SVS	rs671339	G	A	-0.039	0.003	0.737	350470	8.226E-48	206.804	0.0006
PDW	SVS	rs6772399	T	C	0.023	0.003	0.225	350470	2.179E-16	66.920	0.0002
PDW	SVS	rs6900762	G	A	0.036	0.003	0.781	350470	1.219E-36	156.813	0.0004
PDW	SVS	rs6953107	T	C	-0.073	0.003	0.197	350470	3.67E-134	598.817	0.0017
PDW	SVS	rs7192755	G	T	0.021	0.003	0.253	350470	8.722E-15	58.850	0.0002
PDW	SVS	rs7235010	A	G	0.030	0.003	0.784	350470	2.027E-25	105.988	0.0003
PDW	SVS	rs72699866	A	G	0.026	0.003	0.191	350470	2.053E-17	70.830	0.0002
PDW	SVS	rs727088	A	G	0.053	0.002	0.525	350470	1.56E-109	484.161	0.0014
PDW	SVS	rs7503168	G	A	-0.104	0.003	0.172	350470	1E-200	1083.408	0.0031
PDW	SVS	rs752806	T	A	0.020	0.002	0.522	350470	2.053E-17	71.610	0.0002
PDW	SVS	rs7574937	T	C	0.069	0.004	0.097	350470	1.889E-67	294.818	0.0008
PDW	SVS	rs7640885	C	A	-0.023	0.003	0.668	350470	5.961E-20	83.067	0.0002
PDW	SVS	rs7649970	T	C	-0.026	0.004	0.121	350470	8.927E-13	49.746	0.0001
PDW	SVS	rs7696969	G	T	-0.037	0.002	0.619	350470	2.276E-51	222.657	0.0006
PDW	SVS	rs7701346	G	A	-0.041	0.002	0.573	350470	1.549E-64	286.478	0.0008
PDW	SVS	rs7711939	C	T	0.018	0.002	0.645	350470	1.666E-13	53.157	0.0002
PDW	SVS	rs7719425	C	T	0.027	0.003	0.203	350470	5.276E-20	82.969	0.0002
PDW	SVS	rs7768878	A	G	0.046	0.007	0.028	350470	2.209E-10	39.398	0.0001
PDW	SVS	rs7769393	G	A	-0.048	0.003	0.216	350470	1.697E-63	276.686	0.0008
PDW	SVS	rs7830388	G	T	0.034	0.002	0.454	350470	2.102E-46	201.148	0.0006
PDW	SVS	rs7847758	T	C	0.019	0.003	0.784	350470	1.4E-10	41.817	0.0001
PDW	SVS	rs78895525	T	C	-0.036	0.003	0.220	350470	1.149E-35	155.416	0.0004
PDW	SVS	rs7936105	C	T	-0.017	0.002	0.417	350470	2.023E-12	48.596	0.0001
PDW	SVS	rs7958679	T	C	0.157	0.005	0.074	350470	1E-200	1188.629	0.0034
PDW	SVS	rs892090	G	T	0.081	0.003	0.835	350470	1.66E-142	632.072	0.0018
PDW	SVS	rs941207	G	C	-0.061	0.003	0.268	350470	1.25E-115	510.930	0.0015
PDW	SVS	rs9534458	A	G	0.061	0.002	0.652	350470	3.37E-132	585.345	0.0017
PDW	SVS	rs9549753	A	C	0.045	0.002	0.532	350470	1.534E-79	350.291	0.001
PDW	SVS	rs9636612	G	A	0.026	0.002	0.373	350470	2.306E-26	111.208	0.0003
PDW	SVS	rs9890401	G	T	0.021	0.002	0.629	350470	2.754E-17	71.474	0.0002
PDW	CES	rs1002707	T	C	0.040	0.003	0.250	350470	2.054E-49	214.106	0.0006
PDW	CES	rs10067280	T	C	-0.026	0.002	0.644	350470	3.093E-25	105.535	0.0003
PDW	CES	rs10077980	T	C	-0.033	0.003	0.252	350470	6.281E-34	144.845	0.0004

Table S1. SNPs information of platelet indices (PLT, PDW, MPV and PCT) with Stroke and stroke subtypes group (ALS, LAS, SVS and CES)

Exposure	Outcome	SNP	Effect allele	Other allele	Beta	SE	Eaf	N	*P* val	Fval	R2
PDW	CES	rs10774624	A	G	-0.018	0.002	0.514	350470	1.273E-13	54.718	0.0002
PDW	CES	rs10820606	C	A	-0.060	0.003	0.228	350470	1.731E-96	438.685	0.0013
PDW	CES	rs10914053	T	C	0.145	0.003	0.826	350470	1E-200	2126.952	0.006
PDW	CES	rs11013176	G	A	-0.023	0.003	0.281	350470	1.618E-18	76.263	0.0002
PDW	CES	rs11121012	G	A	-0.026	0.002	0.646	350470	6.957E-26	109.554	0.0003
PDW	CES	rs114694170	C	T	-0.119	0.005	0.059	350470	1.42E-122	553.713	0.0016
PDW	CES	rs11497367	A	G	0.123	0.006	0.042	350470	6.324E-97	427.909	0.0012
PDW	CES	rs11553699	G	A	0.136	0.004	0.135	350470	1E-200	1532.546	0.0044
PDW	CES	rs11579514	C	T	-0.020	0.003	0.297	350470	1.309E-14	58.183	0.0002
PDW	CES	rs11667509	C	G	0.049	0.002	0.377	350470	1.863E-87	394.414	0.0011
PDW	CES	rs11702425	C	T	-0.017	0.003	0.303	350470	4.562E-11	42.724	0.0001
PDW	CES	rs11731274	G	T	-0.064	0.003	0.176	350470	1.009E-94	417.890	0.0012
PDW	CES	rs11779690	G	A	-0.022	0.002	0.400	350470	4.437E-20	82.624	0.0002
PDW	CES	rs11834197	A	G	-0.024	0.002	0.435	350470	1.17E-24	102.902	0.0003
PDW	CES	rs11852600	G	T	0.017	0.002	0.349	350470	1.193E-11	45.384	0.0001
PDW	CES	rs12166430	G	A	-0.034	0.004	0.098	350470	2.696E-17	70.839	0.0002
PDW	CES	rs12616493	T	C	0.019	0.002	0.556	350470	4.242E-16	65.077	0.0002
PDW	CES	rs12640722	T	A	0.023	0.003	0.700	350470	2.281E-18	74.993	0.0002
PDW	CES	rs12666334	T	G	0.095	0.002	0.421	350470	1E-200	1559.970	0.0044
PDW	CES	rs1344236	G	A	0.019	0.003	0.285	350470	1.242E-12	49.268	0.0001
PDW	CES	rs1385742	T	A	0.034	0.002	0.647	350470	3.657E-42	184.817	0.0005
PDW	CES	rs1530455	C	T	0.038	0.002	0.597	350470	2.229E-55	245.418	0.0007
PDW	CES	rs1719285	A	T	-0.085	0.003	0.157	350470	1.07E-152	678.050	0.0019
PDW	CES	rs1797077	T	C	0.034	0.002	0.480	350470	8.019E-47	203.257	0.0006
PDW	CES	rs1980532	G	A	-0.056	0.002	0.447	350470	1.47E-121	543.750	0.0015
PDW	CES	rs2228367	C	T	0.218	0.009	0.019	350470	2.07E-131	610.180	0.0017
PDW	CES	rs2810490	A	G	-0.018	0.003	0.284	350470	5.4E-12	46.461	0.0001
PDW	CES	rs2876139	C	A	0.033	0.002	0.638	350470	5.623E-40	172.027	0.0005
PDW	CES	rs34834842	G	A	0.103	0.006	0.038	350470	6.417E-62	270.379	0.0008
PDW	CES	rs3748136	A	G	0.087	0.003	0.211	350470	3.73E-198	883.945	0.0025
PDW	CES	rs3761260	A	G	0.158	0.003	0.138	350470	1E-200	2080.684	0.0059
PDW	CES	rs3843751	T	C	0.017	0.003	0.664	350470	3.175E-11	43.181	0.0001
PDW	CES	rs415895	G	C	0.031	0.002	0.645	350470	3.207E-36	154.656	0.0004
PDW	CES	rs4775609	G	T	-0.023	0.002	0.509	350470	3.357E-22	91.992	0.0003
PDW	CES	rs4783186	C	T	0.057	0.004	0.876	350470	3.44E-57	247.929	0.0007
PDW	CES	rs4790718	A	G	-0.089	0.005	0.064	350470	2.11E-75	331.072	0.0009
PDW	CES	rs4904924	A	G	-0.022	0.003	0.797	350470	1.925E-13	52.907	0.0002
PDW	CES	rs4952073	C	T	-0.116	0.003	0.703	350470	1E-200	1980.074	0.0056
PDW	CES	rs540909	C	T	0.052	0.003	0.754	350470	1.395E-80	352.189	0.001
PDW	CES	rs56050846	A	G	-0.024	0.003	0.242	350470	3.135E-18	74.585	0.0002
PDW	CES	rs565176201	G	A	0.031	0.003	0.710	350470	4.335E-31	137.726	0.0004
PDW	CES	rs56956502	A	G	-0.034	0.003	0.248	350470	1.997E-35	151.198	0.0004
PDW	CES	rs5758968	C	T	0.089	0.002	0.492	350470	1E-200	1401.958	0.004
PDW	CES	rs606552	G	A	0.060	0.003	0.247	350470	7.4E-107	476.631	0.0014
PDW	CES	rs60696641	A	G	0.126	0.004	0.079	350470	5.62E-182	812.314	0.0023
PDW	CES	rs6070629	T	G	0.033	0.003	0.763	350470	1.276E-32	140.511	0.0004

Table S1. SNPs information of platelet indices (PLT, PDW, MPV and PCT) with Stroke and stroke subtypes group (ALS, LAS, SVS and CES)

Exposure	Outcome	SNP	Effect allele	Other allele	Beta	SE	Eaf	N	*P* val	Fval	R2
PDW	CES	rs609311	A	C	-0.031	0.003	0.157	350470	4.241E-21	90.895	0.0003
PDW	CES	rs61751937	C	G	0.082	0.007	0.029	350470	1.712E-31	133.043	0.0004
PDW	CES	rs61838222	A	G	-0.025	0.002	0.622	350470	7.343E-25	104.345	0.0003
PDW	CES	rs6533483	A	G	0.020	0.002	0.402	350470	6.009E-16	64.631	0.0002
PDW	CES	rs6558407	T	C	-0.048	0.002	0.424	350470	1.953E-91	402.604	0.0011
PDW	CES	rs671339	G	A	-0.039	0.003	0.737	350470	8.226E-48	206.804	0.0006
PDW	CES	rs6772399	T	C	0.023	0.003	0.225	350470	2.179E-16	66.920	0.0002
PDW	CES	rs6900762	G	A	0.036	0.003	0.781	350470	1.219E-36	156.813	0.0004
PDW	CES	rs6953107	T	C	-0.073	0.003	0.197	350470	3.67E-134	598.817	0.0017
PDW	CES	rs7192755	G	T	0.021	0.003	0.253	350470	8.722E-15	58.850	0.0002
PDW	CES	rs7235010	A	G	0.030	0.003	0.784	350470	2.027E-25	105.988	0.0003
PDW	CES	rs72699866	A	G	0.026	0.003	0.191	350470	2.053E-17	70.830	0.0002
PDW	CES	rs727088	A	G	0.053	0.002	0.525	350470	1.56E-109	484.161	0.0014
PDW	CES	rs7503168	G	A	-0.104	0.003	0.172	350470	1E-200	1083.408	0.0031
PDW	CES	rs752806	T	A	0.020	0.002	0.522	350470	2.053E-17	71.610	0.0002
PDW	CES	rs7574937	T	C	0.069	0.004	0.097	350470	1.889E-67	294.818	0.0008
PDW	CES	rs7640885	C	A	-0.023	0.003	0.668	350470	5.961E-20	83.067	0.0002
PDW	CES	rs7649970	T	C	-0.026	0.004	0.121	350470	8.927E-13	49.746	0.0001
PDW	CES	rs7696969	G	T	-0.037	0.002	0.619	350470	2.276E-51	222.657	0.0006
PDW	CES	rs7701346	G	A	-0.041	0.002	0.573	350470	1.549E-64	286.478	0.0008
PDW	CES	rs7711939	C	T	0.018	0.002	0.645	350470	1.666E-13	53.157	0.0002
PDW	CES	rs7719425	C	T	0.027	0.003	0.203	350470	5.276E-20	82.969	0.0002
PDW	CES	rs7768878	A	G	0.046	0.007	0.028	350470	2.209E-10	39.398	0.0001
PDW	CES	rs7769393	G	A	-0.048	0.003	0.216	350470	1.697E-63	276.686	0.0008
PDW	CES	rs7830388	G	T	0.034	0.002	0.454	350470	2.102E-46	201.148	0.0006
PDW	CES	rs7847758	T	C	0.019	0.003	0.784	350470	1.4E-10	41.817	0.0001
PDW	CES	rs78895525	T	C	-0.036	0.003	0.220	350470	1.149E-35	155.416	0.0004
PDW	CES	rs7936105	C	T	-0.017	0.002	0.417	350470	2.023E-12	48.596	0.0001
PDW	CES	rs7958679	T	C	0.157	0.005	0.074	350470	1E-200	1188.629	0.0034
PDW	CES	rs892090	G	T	0.081	0.003	0.835	350470	1.66E-142	632.072	0.0018
PDW	CES	rs941207	G	C	-0.061	0.003	0.268	350470	1.25E-115	510.930	0.0015
PDW	CES	rs9534458	A	G	0.061	0.002	0.652	350470	3.37E-132	585.345	0.0017
PDW	CES	rs9549753	A	C	0.045	0.002	0.532	350470	1.534E-79	350.291	0.001
PDW	CES	rs9636612	G	A	0.026	0.002	0.373	350470	2.306E-26	111.208	0.0003
PDW	CES	rs9890401	G	T	0.021	0.002	0.629	350470	2.754E-17	71.474	0.0002

Data of Effect allele, Other allele, Beta, SE, Eaf, Sample size and *P* value are came from exposure group. Fval = (N-2)*R2/(1-R2). R2=Beta*(1-Eaf)*2*Eaf. Abbreviation: AIS:Acute ischemic stroke; LAS:large artery atherosclerosis stroke; SVS:small vessel occlusion stroke; CES: cardioembolic ischemic stroke; PLT:Platelet count; PCT:Platelet crit; MPV:Mean platelet volume; PDW: Platelet distribution width; SE: Standard Error; N: Sample size; Pval: *P* Value. Fval: F Value.
