# Supplementary material for: The impact of platelet indices on ischemic stroke: a Mendelian randomization study and mediation analysis
Source: Front Neurol. 2023 Dec 8;14:1302008. doi: 10.3389/fneur.2023.1302008 (PMC10741650; doi:10.3389/fneur.2023.1302008)
Supplement: Supplementary file 2 [file Table_2.pdf]

Supplementary Table S2. Heterogeneity tests of platelet indices on Stroke and stroke subtypes group

Outcome	Exposure	Method	Q	Q *P* val
Stroke	PLT	MR Egger	120.856	0.017
	PLT	IVW	121.491	0.018
	PCT	MR Egger	103.329	0.075
	PCT	IVW	103.394	0.085
	MPV	MR Egger	131.092	0.005
	MPV	IVW	131.101	0.006
	PDW	MR Egger	100.368	0.045
	PDW	IVW	102.767	0.037
SVS	PLT	MR Egger	120.423	0.015
	PLT	IVW	120.554	0.017
	PCT	MR Egger	83.227	0.503
	PCT	IVW	83.634	0.522
	MPV	MR Egger	94.183	0.361
	MPV	IVW	94.216	0.388
	PDW	MR Egger	90.969	0.210
	PDW	IVW	91.196	0.228
LAS	PLT	MR Egger	112.564	0.047
	PLT	IVW	112.654	0.053
	PCT	MR Egger	90.658	0.240
	PCT	IVW	90.910	0.259
	MPV	MR Egger	123.072	0.017
	MPV	IVW	123.858	0.018
	PDW	MR Egger	89.529	0.137
	PDW	IVW	91.776	0.120
AIS	PLT	MR Egger	111.589	0.061
	PLT	IVW	111.710	0.069
	PCT	MR Egger	100.895	0.101
	PCT	IVW	101.206	0.111
	MPV	MR Egger	151.083	0.000
	MPV	IVW	151.084	0.000
	PDW	MR Egger	85.846	0.206
	PDW	IVW	91.641	0.122
CES	PLT	MR Egger	74.290	0.851
	PLT	IVW	75.308	0.849
	PCT	MR Egger	121.514	0.006
	PCT	IVW	122.376	0.006
	MPV	MR Egger	98.778	0.271
	MPV	IVW	99.004	0.290
	PDW	MR Egger	102.327	0.055
	PDW	IVW	103.600	0.054

AIS:Acute ischemic stroke; LAS:large artery atherosclerosis stroke; SVS:small vessel occlusion stroke; CES: cardioembolic ischemic stroke; PLT:Platelet count; PCT:Platelet crit; MPV:Mean platelet volume; PDW: Platelet distribution width; IVW: Inverse variance weighted.
