# Supplementary material for: The impact of platelet indices on ischemic stroke: a Mendelian randomization study and mediation analysis
Source: Front Neurol. 2023 Dec 8;14:1302008. doi: 10.3389/fneur.2023.1302008 (PMC10741650; doi:10.3389/fneur.2023.1302008)
Supplement: Supplementary file 3 [file Table_3.pdf]

Supplementary Table S3. Horizontal pleiotropy tests of platelet indices on stroke and stroke subtypes group

Outcome	Exposure	Egger intercept	SE	Pval
Stroke	PLT	0.002	0.003	0.493
	PCT	0.001	0.002	0.818
	MPV	0.000	0.002	0.940
	PDW	-0.003	0.002	0.176
AIS	PLT	0.001	0.003	0.755
	PCT	0.001	0.003	0.612
	MPV	0.000	0.003	0.982
	PDW	-0.006	0.003	0.026
LAS	PLT	-0.002	0.007	0.790
	PCT	0.003	0.007	0.634
	MPV	-0.005	0.006	0.445
	PDW	-0.009	0.007	0.171
SVS	PLT	0.002	0.006	0.757
	PCT	0.004	0.006	0.526
	MPV	-0.001	0.005	0.859
	PDW	-0.003	0.006	0.654
CES	PLT	-0.005	0.005	0.316
	PCT	-0.004	0.005	0.439
	MPV	0.002	0.004	0.649
	PDW	0.005	0.005	0.318
