# Supplementary figures and images for: The impact of platelet indices on ischemic stroke: a Mendelian randomization study and mediation analysis
Source: Front Neurol. 2023 Dec 8;14:1302008. doi: 10.3389/fneur.2023.1302008 (PMC10741650; doi:10.3389/fneur.2023.1302008)

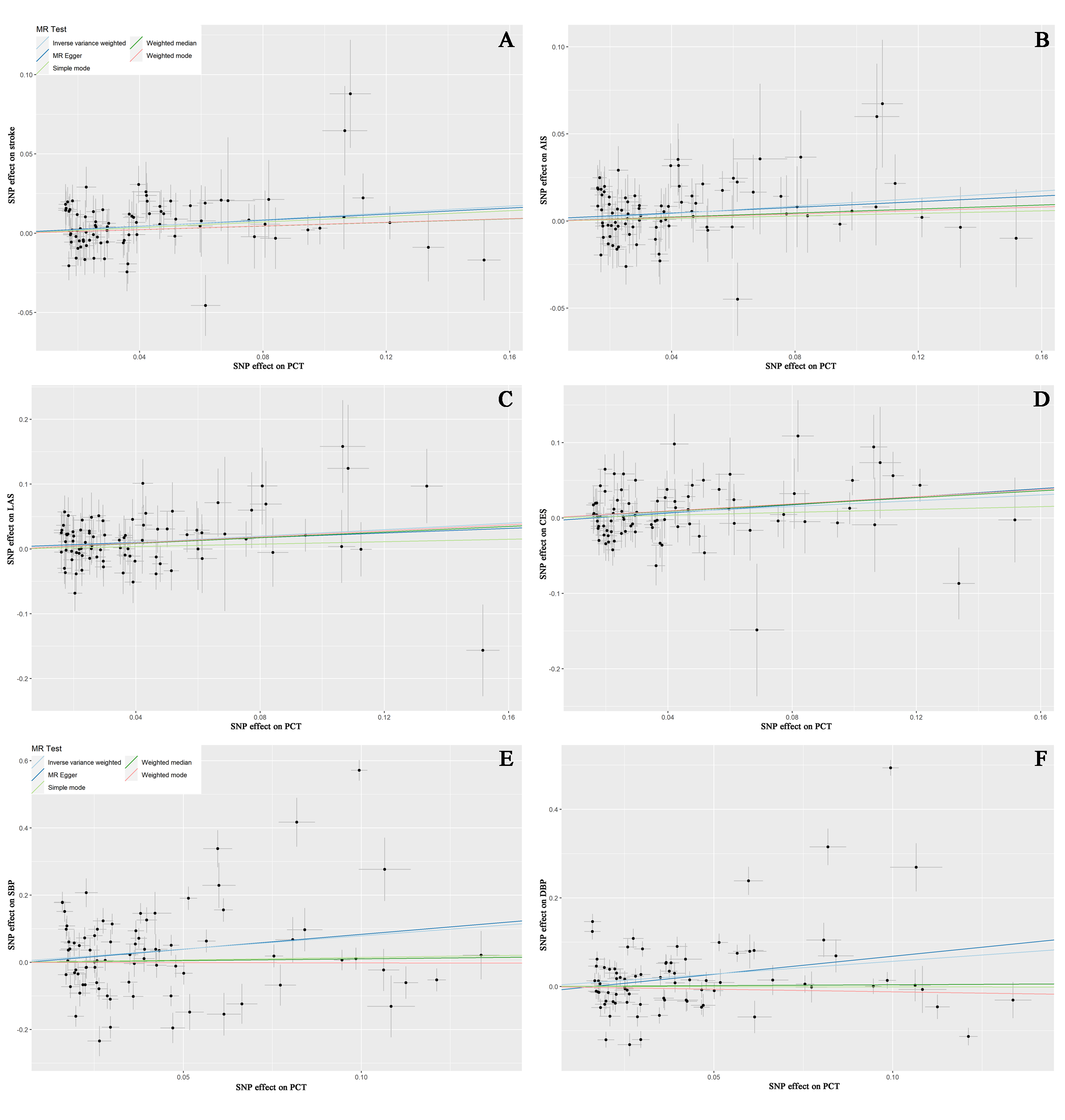

Supplement: Supplementary file 4 [file Image_1.TIF]

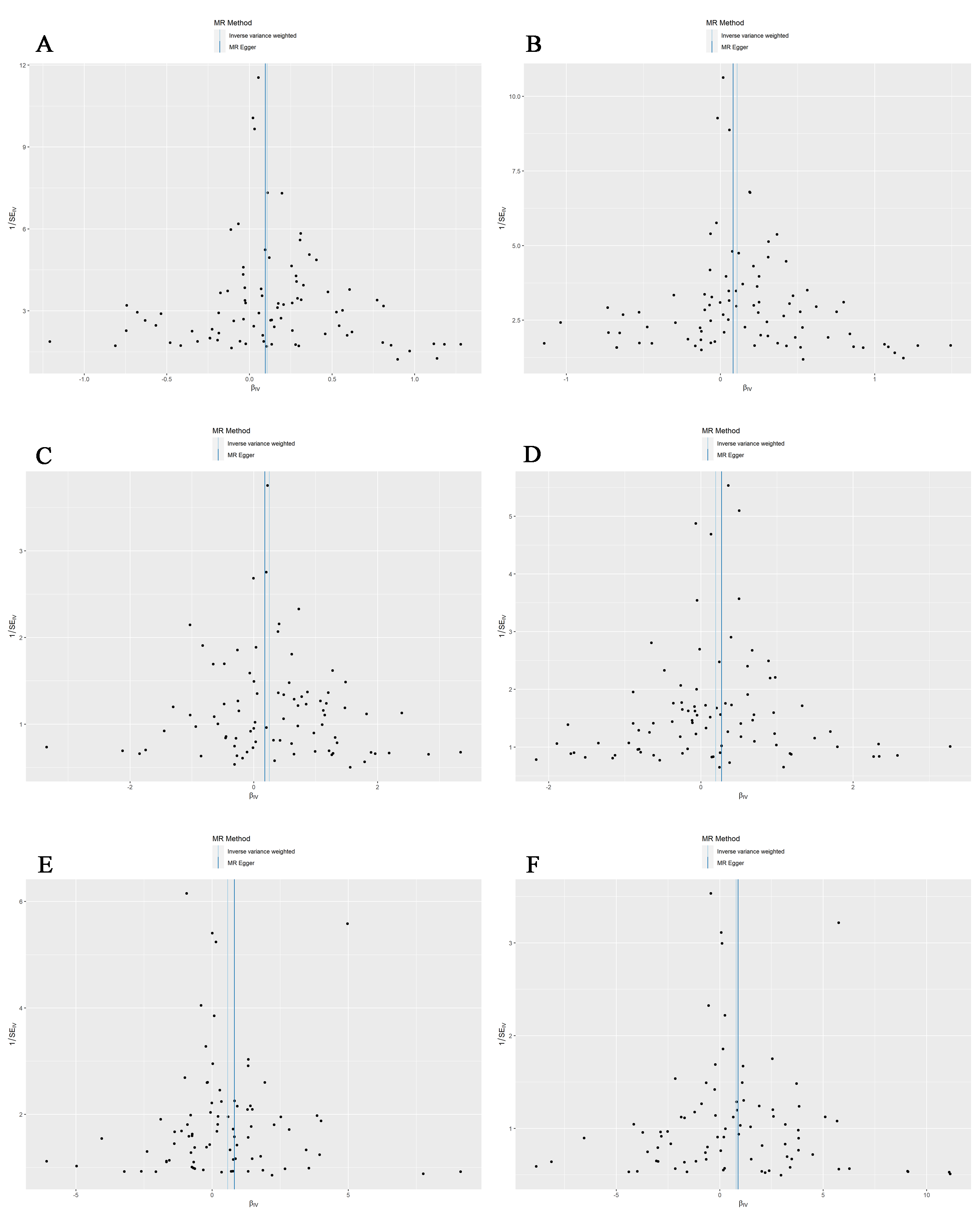

Supplement: Supplementary file 5 [file Image_2.TIF]
